# How do multiple meanings affect word learning and remapping?

**DOI:** 10.3758/s13421-025-01706-z

**Published:** 2025-03-24

**Authors:** Matilde E. Simonetti, Iring Koch, Tanja C. Roembke

**Affiliations:** Institute of Psychology, RWTH Aachen University, Jaegerstr. 17/19, 52066 Aachen, Germany

**Keywords:** Bilingualism, Cross-situational statistical learning, Statistical learning, Language acquisition, Remapping

## Abstract

Many words have multiple meanings. It is currently unclear how a word with more than one meaning is acquired or stored long-term. One possibility is that each word’s meaning is less robustly encoded; this, in turn, may make it easier to acquire additional meanings. We investigated this hypothesis across three cross-situational statistical learning experiments (N_1_ = 62 (in-lab), N_2_ = 61 (online), N_3_ = 60 (online)), using the same general procedure: During Learning Phase 1 (LP1), participants acquired both 1:1 (each word has one meaning) and 1:2 (each word has two meanings) mappings. In Learning Phase 2 (LP2), each word received one new meaning. Across experiments, we manipulated the frequency of 1:1 and 1:2 mappings. As expected, 1:2 mappings were always harder to acquire in LP1. However, in the LP2 of Experiment 1, former 1:1 mappings were remapped more easily than former 1:2 ones, while 1:2 mappings were remapped more easily in Experiments 2 and 3. These data suggest that words with multiple meanings are more easily remapped, indicating that each meaning is less robustly associated with the word, which in turn may facilitate the acquisition of an additional meaning. However, the observed inconsistencies in the remapping results between in-lab and online experiments suggest that CSWL may differ across these two settings.

**Supplementary Information:**

The online version contains supplementary material available at 10.3758/s13421-025-01706-z.

## Introduction

To learn what a word means, one needs to encode a referent (e.g., an animal with four legs and a puffy tail) and its label (e.g., “rabbit”) and create an association between the two (this is called a "mapping"; e.g., McMurray et al., 2012). However, word learning is not straightforward. One reason is that mappings are not always one-to-one (one word maps onto a single meaning; 1:1 mapping). Instead, it is not uncommon for words to have several meanings. For example, (interlingual) homographs, like the word "bat" in English, can refer to the nocturnal flying mammal and the equipment used in baseball to hit the ball (1:2 mapping). Otherwise, the same meaning can be mapped onto multiple words, for example, synonyms and translation-equivalent words in a different language (2:1 mapping). How multiple mappings per word or meaning are acquired has been specifically of interest in the context of bilingualism: While both monolinguals and bilinguals encounter multiple mappings (through, e.g., synonyms), it is nonetheless true that bilinguals are statistically more exposed to them (through translations and interlingual homographs; see, e.g., Martin et al., 2024).

Learning multiple mappings for a word or object can be particularly challenging in situations of referential ambiguity (Quine, 1960), where a word can be linked to many possible referents, making it theoretically difficult to determine its meaning. For instance, if a traveller hears the word "gavagai" while seeing a rabbit, they might struggle to understand its meaning due to multiple possible referents. However, repeated exposure to the word in different contexts would eventually allow the traveller to correctly identify "gavagai" as "rabbit," demonstrating that referential ambiguity can be overcome through repeated learning experiences. Crucially, the traveller is able to learn “gavagai” even if they already have a word attached to the concept (“rabbit”).

As the fictional traveller in this example, people can extract the baseline probability of co-occurring words and referents and use this property to learn mappings correctly in the absence of feedback. Yu and Smith (2007) tested this mechanism to acquire (single meaning) words with monolingual adult participants in a paradigm called cross-situational word learning (CSWL; sometimes also called cross-situational statistical learning). As predicted, participants were able to learn the words across trials, even as every single situation was ambiguous (see Roembke et al., 2023, for a focused review). As the name suggests, CSWL is often considered a form of statistical learning (e.g., Roembke & McMurray, 2016; Weiss et al., 2020). That is, the mechanism underlying learning is thought to be the gradual accumulation of co-occurrence statistics, where mappings between words and meanings are strengthened over time.

Most studies with CSWL were implemented only with one meaning for each word (1:1 mappings; e.g., Escudero et al., 2016; Roembke & McMurray, 2016; Wang, 2020; Yu & Smith, 2007). Fewer studies have used different combinations of words and objects (e.g., Aguasvivas et al., 2024; Poepsel & Weiss, 2016), the so-called multiple mappings. In particular, 1:2 or 2:1 mappings are primarily used. One common finding in these studies is that multiple mappings are more complex to acquire than single ones (e.g., Benitez & Li, 2024; Benitez et al., 2016; Kachergis et al., 2012). While this makes intuitive sense, how multiple mappings are learned precisely remains a matter of discussion. In this paper, we will argue that, compared to single mappings, multiple mappings are more flexibly stored overall. Thus, it is easier for learners to remap them when needed.

### Learning multiple mappings: The role of competition and frequency

There are several different, non-mutually exclusive reasons why multiple mappings may be harder to learn than single ones: (1) Different meanings (for 1:2 mappings) or words (for 2:1 mappings) may compete with each other; such competition may make it harder to encode a new meaning/word as well as reactivate it as necessary (see, e.g., Benitez et al., 2016). Competition may be especially high in statistical word learning, where words and meanings are not explicitly paired. (2) Multiple mappings may be overall less frequent than single mappings and are therefore less expected (Kachergis et al., 2009). This may be the case both due to a person’s language background (e.g., a monolingual person may be less likely to encounter multiple mappings than a bilingual one; we will term this *long-term frequency*) and/or due to the specific learning situation a person is in (e.g., the frequency of multiple mappings within an experimental session; *short-term frequency*). As a result, each association within the multiple mapping may end up weaker.

The role of competition in the statistical word learning of multiple mappings was investigated by Benitez and colleagues (2016). In their study, the second word of 2:1 mappings was blocked through time. That is, monolingual and bilingual participants completed a first learning block with only 1:1 mappings and a second block in which some objects were mapped onto a second name (creating both 1:1 and 2:1 mappings) in a CSWL experiment. Benitez and colleagues (2016) found that the competition between the two referents was reduced but did not disappear in both groups compared to when 1:1 and 2:1 mappings were mixed throughout learning. It seems that separating words in time made it easier to learn both meanings and reduced item-level competition, even if some competition remained (see also Yurovsky et al., 2013). These findings suggest that competition within multiple mappings slows down their learning.

Evidence for the role of long-term frequency in the acquisition ease of multiple mappings comes from the finding that bilingual children are less likely than their monolingual peers to use the mutual exclusivity bias – the tendency to assume that there is just one label for every object (Merriman et al., 1989). In fact, bilinguals are more likely than monolinguals to map a second meaning on a word that already has one (e.g., Byers-Heinlein & Werker, 2009; Houston‐Price et al., 2010), suggesting that bilinguals may adapt their learning biases and strategies to accommodate better the type of word mappings they encounter regularly. Consistent with this, in a CSWL study by Poepsel and Weiss (2016), bilinguals were more accurate than monolinguals, but only with 1:2 mappings and not with 1:1 mappings. However, it should be noted that Aguasvivas et al. (2024) could not replicate this finding using both 2:1 and 1:2 mappings. Poepsel and Weiss (2016) explained their result by saying that bilinguals learn and retain words more flexibly than monolinguals. That is, bilinguals may be more open to remapping a word’s meaning because they are accustomed to words having multiple meanings and to objects or concepts being described by different words (see also McMurray et al., 2012, for computational results). In line with this way of thinking, Wang and Mintz (2018) found that monolinguals expected a 1:1 mapping in a CSWL experiment when they did not receive information about the type of mapping they are being exposed to. Most of these findings are consistent with the idea that long-term exposure to words with multiple meanings – for example as a result of bilingualism – may impact how new words are acquired. Such longer-term adaptations could be implemented through a tuning of the mutual exclusivity bias, as described previously.

In addition, there is also evidence that the short-term frequency of multiple mappings impacts how easily they can be learnt: Kachergis and colleagues (2017) found that having different frequencies for different words (in their case, only for single mappings) can boost learning. Regarding multiple mappings, as described previously, it has been reported that they are usually more challenging to acquire than single mapping words in CSWL experiments (e.g., Benitez & Li, 2024; Benitez et al., 2016; Kachergis et al., 2012). One exception is a CSWL study by Chan and Monaghan (2019), where all participants, regardless of being monolingual or bilingual, learned 2:1 mappings better than 1:1 ones. The authors reasoned that this finding resulted from their particular experimental design, as participants had to acquire more 2:1 than 1:1 mappings. Given that language background did not impact how well different mapping types were acquired, this suggests that the higher local short-term frequency of multiple mappings within the experiment determined the ease of acquisition. All together, these results indicate that the frequency by which one is exposed to multiple mappings (or not) may impact how easily they can be acquired. However, it is currently unclear whether the acquisition of these mappings is more determined by a long-term exposure to them (e.g., as part of someone’s specific language background) or can also be determined by their short-term frequency.

**Fig. 1:**
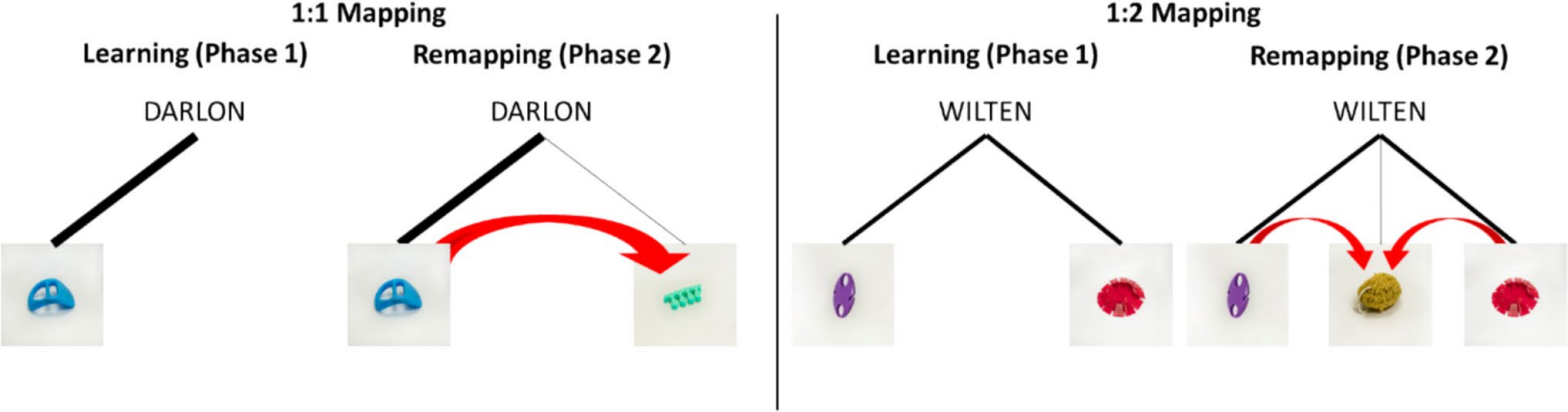
Competition between objects for both mapping types. A word connected to one object (like DARLON; 1:1 mapping) is expected to produce more proactive interference (depicted as an arrow) than a word connected to two objects (like WILTEN; 1:2 mapping). This is because the single association in a 1:1 mapping is stronger than each individual association in a 1:2 mapping, making it more likely to compete with new associations during remapping. In contrast, the associations in a 1:2 mapping are weaker, which may make them less likely to compete with any novel referentThe current study

An interesting prediction derived from the hypothesis that multiple mappings are encoded less robustly (either due to increased competition or lower frequency) is that they should be overall more flexible and thus easier to remap (which in turn may be advantageous in some learning contexts). That is, for example, even as it is harder to learn a word with two meanings, it may at the same time also be easier to add a third (or fourth, etc.) meaning to it, either because a person initially encoded an incorrect meaning or because the word has additional true meanings (e.g., when learning a new language). At a mechanistic level, such a *remapping advantage* for 1:2 mappings relative to 1:1 mappings could derive from increased competition and proactive interference for occurring 1:1 mappings. That is, the single original association that is part of the 1:1 mapping may be stronger than each individual association in a 1:2 mapping, which in turn may increase interference when learning another meaning. In turn, the weaker connections that are part of a 1:2 mapping may result in less interference (Anderson, 1992; Shiffrin, 1999). Reduced proactive interference for 1:2 mappings could then also lead to lower interference for a new meaning and, therefore, reduced associative blocking of the new meaning by the existing connections (see Fig. 1 for a schematic representation; Yurovsky et al., 2013). Alternatively, it is also possible that not the strength of the existing connections determines the level of interference, but their number – if this were the case, we would expect easier remapping for 1:1 than 1:2 mappings.

The current study aims to investigate the remapping of acquired 1:1 and 1:2 word-object mappings as part of statistical word learning, thus asking whether the two types of mappings differ in their robustness, where less rigid mappings may allow for more flexible learning. We conducted three CSWL experiments that were each divided into two phases (Learning Phase 1, LP1, and Learning Phase 2, LP2). During LP1, participants were always exposed to both 1:1 and 1:2 mappings. In LP2, participants were again exposed to the same words, but each word received a new meaning (a similar procedure was used in Bulgarelli et al., 2021). All mappings in LP2 were 1:1. Participants were always German-English unbalanced bilinguals. We decided to test the remapping of single and multiple mappings in bilinguals for several reasons. First, at least half of the population is bilingual (Grosjean, 2010; i.e., being proficient in more than one language), and the number is even higher in Europe. Despite this, there is a monolingual bias in language research, where monolinguals are studied more frequently and assumed to be the baseline (Auer, 2007; De Houwer, 2023; Genesee, 2022). Second, it would be impossible to recruit monolinguals in Germany, and monolinguals from other countries would likely not be matched to our sample in many variables other than language history. Third, our focus was on the representational nature of multiple mappings, which should be more the norm in bilinguals than in monolinguals, as well as the role of their short-term frequency (rather than the long-term one) during the learning and remapping of words with multiple meanings. The three experiments in this study were as follows:

1. Experiment 1 is an in-lab experiment (pre-registered: https://doi.org/10.23668/psycharchives.12909)
2. Experiment 2 is an online replication of Experiment 1 (pre-registered: https://doi.org/10.23668/psycharchives.14033)
3. Experiment 3 was again conducted online; we changed the frequencies of the two mapping types in LP1 in comparison to Experiments 1 and 2 (pre-registered: https://doi.org/10.23668/psycharchives.14479).

The general procedure of the three experiments was identical and will only be described in detail as part of the Methdo of Experiment 1. Minor methodological differences between experiments will be highlighted when introducing Experiments 2 and 3. For LP1, following the literature (e.g., Benitez et al., 2016; Poepsel & Weiss, 2016), we hypothesised that accuracy for 1:2 mappings would be higher than for 1:1 mappings (H1). Regarding the remapping, in LP2, we hypothesised that we would not find an overall difference in the remapping of 1:1 and 1:2 mappings (H2a) based on the results of two pilot experiments^1^ (conducted online). Instead, we predicted that it would be easier to remap 1:2 than 1:1 mappings at the beginning (operationalised as Block 1) of LP2 (H2b). We also hypothesised that this difference would hold even if accuracy at the end of LP1 (Block 5) is controlled for (H2c).

If 1:2 mappings are indeed learned worse than 1:1 mappings in LP1, this would show that multiple mappings are harder to encode, which is in line with previous studies (e.g., Benitez et al., 2016; Poepsel & Weiss, 2016). Most importantly, if we find evidence in favour of easier remapping of 1:2 mappings than 1:1 mappings in LP2, this would suggest that multiple mappings are indeed encoded less robustly, but that this also comes with an increased flexibility for future word learning. Finally, manipulating the frequency of the different mapping types across experiments allowed us to examine to what extent any observed learning and remapping advantages may be due to how many multiple mappings were recently experienced (i.e., their short-term frequency).

While using participants’ reaction times (RTs) as a measure of processing and acquisition ease is extremely common in cognitive psychology, RTs have been rarely used in the context of CSWL (for an exception, see Escudero et al., 2016). Measuring RTs would provide us an interesting measure to judge the speed of retrieval. Since the literature on RTs in CSWL is sparse, we only conducted exploratory analyses regarding RTs. However, we speculated based on increased competition for 1:2 mappings that they should overall be more difficult to retrieve, thus causing higher RTs. We will explore if there is a difference between 1:2 and 1:1 mappings in LP1 (E1), all of LP2 (E2a), only at the beginning (Block 1) of LP2 (E2b), and when accuracy at the end of LP1 (Block 5) is controlled for (E2c).

## Experiment 1 (In-lab)

### Methods

#### Participants

Sixty-six unbalanced German-English bilinguals participated in the experiment, and 62 were included in the final sample. They were recruited through the university (RWTH Aachen University) mailing list. Participant demographics for all experiments are summarised in Table 1. Participants were native speakers of German but considered themselves fluent in English and had at least 7 years of English schooling. Language proficiency for bilinguals was assessed through the German and English LexTALE (Lemhöfer & Broersma, 2012) and language questionnaires (Kaushanskaya et al., 2020). Participants were compensated through course credit or reimbursement (10€/h).

**Table 1:** Demographics from the three experiments for the final samples

	General			
Characteristic	Exp. 1	Exp. 2	Exp. 3	Comparisons –p-value^a^
N	62	61	60	
Age, y (M (SD))	23.92 (4.66)	22.51 (2.61)	23.07 (3.84)	0.119^c^
Gender				0.233^d^
Female	49 (79%)	50 (82%)	40 (67%)	
Male	13 (21%)	9 (15%)	17 (28%)	
Non-binary	0 (0%)	1 (1.6%)	1 (1.7%)	
I prefer not to answer	0 (0%)	1 (1.6%)	2 (3.3%)	
Education				0.119^d^
High school	37 (60%)	46 (75%)	47 (78%)	
Training qualification	3 (4.8%)	4 (6.6%)	4 (6.7%)	
University degree	21 (34%)	11 (18%)	9 (15%)	
PhD	1 (1.6%)	0 (0%)	0 (0%)	
English LexTALE, M (SD)	0.75 (0.11)	0.76 (0.11)	0.72 (0.12)	0.785^c^
German LexTALE, M (SD)	0.87 (0.06)	0.87 (0.07)	0.85 (0.07)	0.248^c^
	L2			
Characteristic	Exp. 1	Exp. 2	Exp. 3	
n (% of N)	43 (69%)	58 (95%)	56 (93%)	
AoA, M (SD)^b^	7.13 (2.11)	7.55 (1.92)	7.86 (2.15)	0.227^c^
Proficiency speaking L2, M (SD)	7.33 (1.07)	7.21 (0.91)	7.30 (1.03)	0.795^c^
Proficiency understanding L2, M (SD)	8.35 (0.72)	7.81 (1.10)	7.98 (0.98)	0.022*^e^
Proficiency reading L2, M (SD)	8.35 (1.00)	7.72 (1.12)	7.89 (0.93)	0.010**^e^

^a^ The full results of the comparison tests can be found in the Online Supplementary Materials

^b^ L2 AoA: Age at which the second language was acquired

^c^ *p*-value from a between-subjects ANOVA

^d^ *p*-value from a Fisher’s exact test

^e^ Participants in Experiment 1 are significantly different from Experiment 2, but not from Experiment 3, for Understanding (*p* = 0.017) and for Reading (*p* = 0.008)

For each characteristic, the mean response and standard deviation (in brackets) are provided. We do not have information on all participants’ language backgrounds because they answered for languages other than L1 German and L2 English. Based on the LexTALE Test, we can conclude that they are nevertheless German-English bilinguals

*Power analyses.* We performed several power analyses using simulation-based power estimation (Kumle et al., 2021), one for each non-exploratory hypothesis. We used one of the pilot experiments (*N* = 42; conducted online) as a reference. To obtain a power of over 80%, we needed:

- H1: 60 participants (82% for interaction between mapping and block in LP1).
- H2b: 50 participants (87.1% for the main effect of former mapping in Block 1 of LP2).
- H2c: 50 participants (84.4% for the main effect of former mapping in Block 1 of LP2 after controlling for accuracy at the end of LP1).

Based on these power analyses, we pre-registered a sample size of 60 participants for each experiment.

*Inclusion criteria.* Participants had to be between 18 and 35 years old, self-report normal or corrected-to-normal vision, and have never been diagnosed with a learning or language disorder. In addition, participants were required to score above 70% in the German LexTALE (Lemhöfer & Broersma, 2012) and above 50% in the English LexTALE (Lemhöfer & Broersma, 2012). Lastly, participants needed to reach 40% accuracy in the whole experiment (chance level: 33%) to guarantee that they engaged with the learning task. In this experiment, we excluded one participant who did not reach 70% in the German LexTALE and three participants due to at-chance learning performance. All inclusion criteria were pre-registered.

#### Materials

Twelve bisyllabic CVCV nonwords were selected: BERNAL, DARLON, GLANKE, GRINTER, MALFEN, MURLER, RAUPLET, STAUNKER, THERNUS, VARTION, WILTEN, and SUMPER. These nonwords were plausible in both English and German, following both languages' phonotactic and orthographic rules. The selection process of the words involved a survey where ten English monolinguals and ten unbalanced German-English bilinguals evaluated 29 bisyllabic nonwords using a 5-point Likert scale ranging from “definitely not English/German” to “definitely English/German.” The average score for each word for both groups was calculated along with the difference between the two. Words with a difference greater than one point were excluded, and stimuli with the greatest average acceptability score were selected. On average, the selected stimuli were rated as 3.11 (*SD* = 0.22). Additionally, 27 coloured photographs of unusual objects were used in the experiment. While the objects are not part of a normative database, they have been used in other word-learning experiments before (e.g., in Roembke & McMurray, 2021).

Two-thirds of the words (eight) were randomly assigned to only one target referent (1:1 mapping), whilst the other third (four) had two targets (1:2 mapping, similar to Poepsel & Weiss, 2016). Five different randomisation lists were created using Matlab; each participant saw just one list. Each list's order of words was randomised within the set of 12 words, with the constraint that no direct repetitions could occur across sets. For 1:2 mappings, there was an additional constraint: The two meanings were taking turns (i.e., if Word 1 was presented together with Target 1 the last time, in the current trial, Target 2 will be present).

#### Procedure

Participants were tested one by one in a lab room with a computer. They first signed the informed consent and a data protection form. They then performed the German and English LexTALE (Lemhöfer & Broersma, 2012) using the online platform (https://www.lextale.com/takethetest.html). Subsequently, they completed the experiment that was programmed in SR Research Experiment Builder 2.4.77 (SR Research Experiment Builder, 2020). Lastly, they filled out a shortened version of the LEAP-Q (Kaushanskaya et al., 2020). Due to a technical error, the last 40 participants filled out a slightly different version of the questionnaire that contained additional questions.

The experiment was divided into two phases (LP1 and LP2), both with five blocks (overall number of trials = 480, equally divided between blocks and phases). In an individual trial, participants first saw three objects (one at the top and two at the bottom, or vice versa) together with a blue dot for 1,000 ms. One of the three objects was always the target; the target's position was counterbalanced across trials. When the dot turned red, participants had to click on it to make the word (written in capital letters) appear in the centre of the screen. To go to the next trial, participants had to click on the object they thought the word mapped onto. They never received any feedback. Participants were instructed to choose randomly at the beginning, but their responses should become more informed over time. They were also told to answer as accurately and fast as possible. At the beginning of LP2, participants were told that they would now learn new meanings for the previously seen words.

#### Design

The dependent variable (DV) for the main hypotheses was always accuracy, while for the exploratory ones, it was RT. For both DVs in LP1, there were two within-subject independent variables: mapping type (with two levels: 1:1, 1:2) and block (1–5), as well as their interaction. The same model structure was applied in LP2, but here, the independent variables were the former mapping type and block with the same levels (and their interaction).

### Results

We conducted all the analyses in R (R Core Team, 2024) using linear mixed models for our hypotheses in all experiments. We contrast coded (former) mapping type (1:1, 1:2; contrast coded as + 0.5/- 0.5) and centred block (1–5). As random effects, we always considered participant, word, and target object. To choose the best random effect structure, we used the function buildmer in the buildmer package (v. 2.8, Voeten & Voeten, 2021). This function uses a backwards-fitting model selection procedure that starts from the maximal model and gives us a model that converges by systematically ruling out random slopes. The specific models for each analysis are in the supplementary materials. We tested all our hypotheses using general linear mixed models with the package lme4 (Bates et al., 2015), with accuracy (1/0) as the DV. If a significant interaction was found, we conducted post hoc tests, splitting the data for one of the variables.

**Fig. 2:**
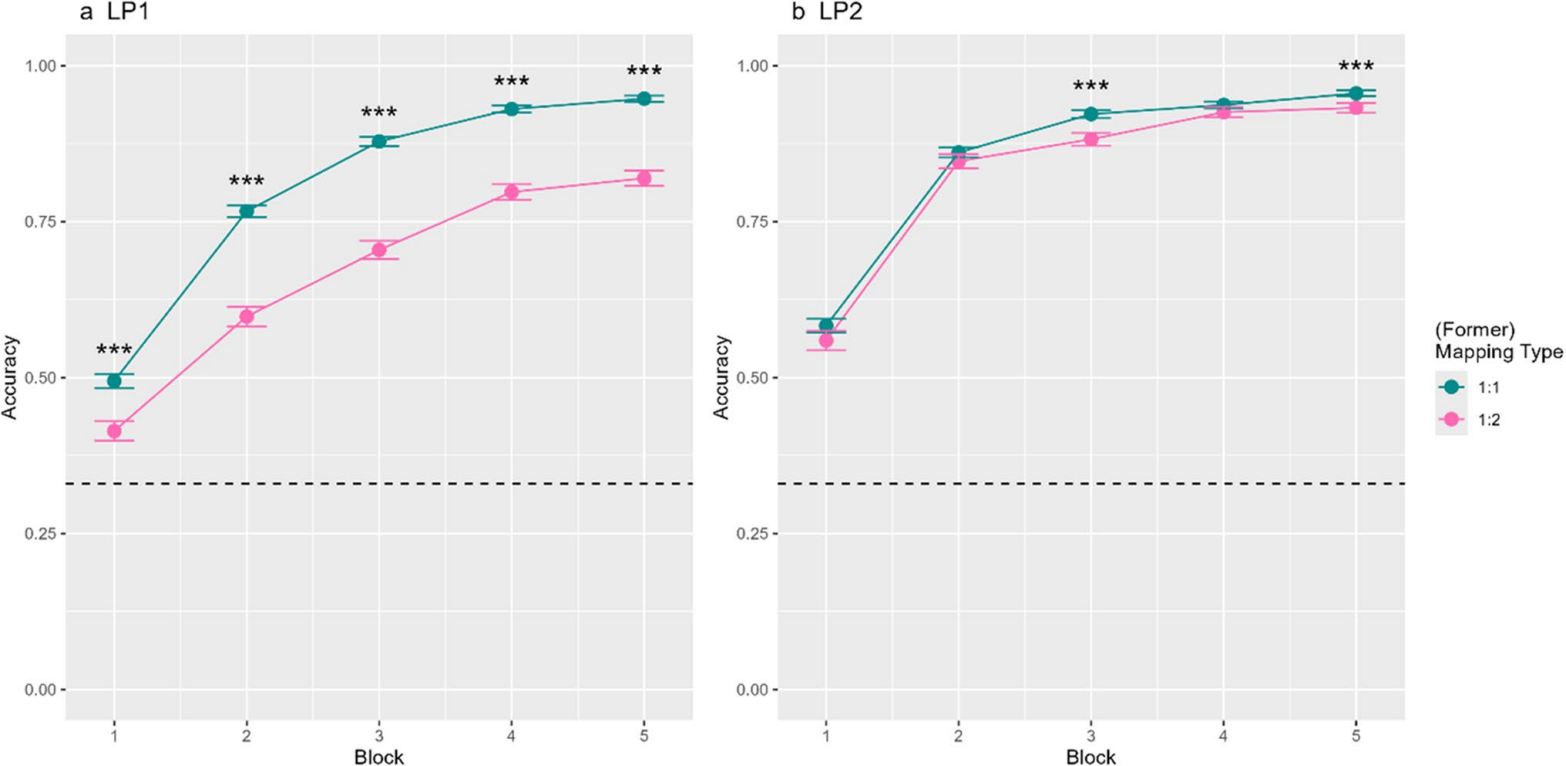
Accuracy performance in both phases of Experiment 1.2a. The left figure depicts accuracy performance in LP1 of Experiment 1 per mapping type (1:1, 1:2) and block (1–5). 2b. The right figure depicts accuracy performance in LP2 of Experiment 1 per former mapping type (1:1, 1:2) and block (1–5). The dotted line indicates chance performance. Errors bars are the standard error of the mean. Asterisks (***) indicate significant differences between mean accuracy per (former) mapping type and block at p < 0.001

*Main analyses.* We first examined accuracy in LP1 and performance differences across mapping type and block (H1). Participants learnt words quite well in the first phase (*M* = 0.76, *SD* = 0.43) and scored significantly above chance (chance level = 0.333, *t*(61) = 34.21, *p* < 0.001). We found a significant main effect of mapping (β = −1.25, *SE* = 0.10, *z* = −12.41, *p* < 0.001) as well as of block (β = 0.86, *SE* = 0.06, *z* = 15.39, *p* < 0.001). As expected, participants were more accurate with 1:1 (*M*_*1:1*_ = 0.80) than with 1:2 mappings (*M*_*1:2*_ = 0.67). We also found a significant interaction between mapping type and block (β = −0.44, *SE* = 0.04, *z* = −11.75, *p* < 0.001; see Fig. 2a). While there was a significant difference between the two mapping types in every block (*p* always < 0.002 and *z* below −3.21), Fig. 2 suggests that the significant interaction stems from a steeper learning rate for 1:1 than 1:2 mappings at the beginning of the experiment. Together, these results are consistent with H1 (1:2 mappings are harder to acquire than 1:1 mappings).

We then examined accuracy in LP2 and performance differences across mapping type and block (H2a). Participants also learnt words well in the second phase (*M* = 0.84, *SD* = 0.36) and scored significantly above chance (chance level = 0.333, *t*(61) = 33.43, *p* < 0.001). We observed a significant main effect of former mapping type (β = −0.39, *SE* = 0.07, *z* = −5.59, *p* < 0.001) and block (β = 1.50, *SE* = 0.12, *z* = 13.04, *p* < 0.001). Contrary to our hypothesis, participants were generally more accurate in remapping former 1:1 mappings (*M*_*1:1*_ = 0.85) than former 1:2 mappings (*M*_*1:2*_ = 0.83). Additionally, there was a significant interaction between former mapping type and block (β = −0.15, *SE* = 0.04, *z* = −3.37, *p* < 0.001; see Fig. 2b). Specifically, a significant difference between the two mapping types was found in Blocks 3 and 5 (*p* < 0.001). Contrary to our prediction H2a, 1:1 mappings were easier to remap than 1:2 ones at the end of LP2.

As pre-registered, we examined differences in remapping in Block 1 of LP2 (H2b and H2c; dropping block as a fixed factor). We did not find a significant main effect of former mapping type (*p* = 0.206). Contrary to our prediction (H2b), participants were not more accurate with former 1:2 mappings (*M*_*1:2*_ = 0.41) than with former 1:1 ones (*M*_*1:1*_ = 0.49). To test H2c, we included the accuracy of the last block of LP1 as a covariate to our model. Contrary to our prediction (H2c), we again did not find a significant main effect of the former mapping type (*p* = 0.375).

*Exploratory analyses.* We first examined RTs (log-transformed, only for correct trials and below 10,000 ms) in LP1 and performance differences across mapping type and block (E1; means and SDs are summarised in Table 2). We found a significant main effect of mapping (β = 0.36, *SE* = 0.02, *t* = 14.42, *p* < 0.001) and block (β = −0.14, *SE* = 0.01, *t* = −23.35, *p* < 0.001). Participants were faster with 1:1 (*M*_*1:1*_ = 1,934 ms) than with 1:2 (*M*_*1:2*_ = 2,779 ms) mappings. We also found a significant interaction between mapping and block (β = 0.05, *SE* = 0.01, *t* = 7.24, *p* < 0.001). As for accuracy, there was a significant difference between the two mapping types in every block (*p* always < 0.001) and the difference grows larger in later blocks.

**Table 2:** Reaction time (RT; ms) performance in both phases of the three experiments

			**BLOCK**				
			1	2	3	4	5
**EXPERIMENT**	**Phase**	**Mapping**	Mean (SD)	Mean (SD)	Mean (SD)	Mean (SD)	Mean (SD)
**1**	1	1:1	3,179 (1,827)	2,238 (1,461)	1,784 (1,156)	1,611 (1,001)	1,512 (917)
		1:2	3,605 (1,801)	3,114 (1,744)	2,689 (1,632)	2,597 (1,636)	2,386 (1,464)
	2	1:1	2,626 (1,555)	1,898 (1,231)	1,505 (865)	1,411 (758)	1,339 (750)
		1:2	2,733 (1,607)	1,948 (1,249)	1,587 (983)	1,408 (728)	1,330 (670)
**2**	1	1:1	2,563 (1,341)	2,088 (1,279)	1,692 (1,003)	1,520 (818)	1,480 (731)
		1:2	2,874 (1,529)	2,743 (1,486)	2,546 (1,445)	2,479 (1,507)	2,250 (1,363)
	2	1:1	2,410 (1,450)	1,900 (1,140)	1,654 (1,013)	1,474 (829)	1,419 (718)
		1:2	2,276 (1,377)	1,821 (1,095)	1,574 (903)	1,498 (951)	1,382 (740)
**3**	1	1:1	2,756 (1,356)	2,217 (1,326)	1,943 (1,351)	1,709 (1,101)	1,630 (1,017)
		1:2	2,876 (1,412)	2,765 (1,631)	2,856 (1,820)	2,644 (1,642)	2,485 (1,642)
	2	1:1	2,464 (1,675)	1,901 (1,202)	1,677 (922)	1,581 (953)	1,463 (789)
		1:2	2,390 (1,481)	1,956 (1,223)	1,670 (997)	1,563 (931)	1,464 (824)

The means and SDs of the three experiments are divided into blocks, mapping, and phases.

For LP2 (E2a), we found only a significant main effect of block (β = −0.15, *SE* = 0.01, *z* = −16.20, *p* < 0.001). Participants were indeed faster as time passed by. Lastly, we examined RTs at the beginning of LP2 (E2b and E2c). However, we did not find a significant main effect of former mapping type in both analyses (complete analyses can be found in the supplementary material).

### Discussion

In Experiment 1, we could confirm our hypothesis for LP1: Participants were indeed more accurate with 1:1 mappings than with 1:2 ones (and RTs corroborated this finding), consistent with previous studies (e.g., Benitez et al., 2016; Poepsel & Weiss, 2016). However, we unexpectedly found that 1:1 mappings were generally easier to remap than 1:2 ones in LP2 (especially in later blocks). This result was against our predictions and thinking that multiple mappings would be easier to remap due to weaker connections between a word and each potential referent. Due to these surprising findings inconsistent with our two (online) pilots, we decided to replicate the experiment. Since both of the pilots in which we found the expected results were conducted online, we decided to conduct Experiment 2 online as well. Apart from the platform on which the experiment was conducted, Experiment 2 is an exact replication of Experiment 1.

## Experiment 2

### Methods

#### Participants

Sixty-two unbalanced German-English bilinguals participated in the online experiment, and 61 were included in the final sample (one participant failed to reach an overall 40% accuracy and was therefore excluded). The recruitment criteria and rationale were the same as in Experiment 1.

#### Materials and Procedure

The materials were the same as in Experiment 1. The procedure of this experiment was identical to Experiment 1, but Experiment 2 was conducted online via Gorilla Experiment Builder (Anwyl-Irvine et al., 2020). Additionally, in the online version of the LexTALE test (Lemhöfer & Broersma, 2012), participants only had 5 s to respond instead of unlimited time (to avoid the possibility of looking up words on the internet). Importantly, this time limit was only applied to LexTALE, not other parts of the experiment.

### Results

**Fig. 3:**
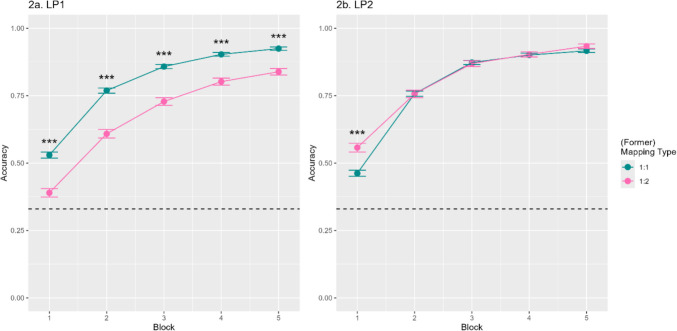
Accuracy performance in both phases of Experiment 2. 3a. The left figure depicts accuracy performance in LP1 of Experiment 2 per mapping type (1:1, 1:2) and block (1–5). 3b. The right figure depicts accuracy performance in LP2 of Experiment 2 per former mapping type (1:1, 1:2) and block (1–5). The dotted line indicates chance performance. Errors bars are the standard error of the mean. Asterisks (***) indicate significant differences between mean accuracy per (former) mapping type and block at p < 0.001

*Main analyses.* As in Experiment 1, we first examined accuracy in LP1 and performance differences across mapping type and block (H1). Learning rate was again high (*M* = 0.76, *SD* = 0.43), and performance was significantly above chance (chance level = 0.333, *t*(59) = 27.41, *p* < 0.001). We observed a significant main effect of mapping type (β = −1.12, *SE* = 0.14, *z* = −7.77, *p* < 0.001) as well as a significant effect of block (β = 0.86, *SE* = 0.06, *z* = 14.12, *p* < 0.001). Participants were again more accurate with 1:1 mappings (*M*_*1:1*_ = 0.80) compared to 1:2 mappings (*M*_*1:2*_ = 0.67). Additionally, there was a significant interaction between mapping type and block (β = −0.24, *SE* = 0.04, *z* = −6.34, *p* < 0.001; see Fig. 3a). Specifically, there was again a significant difference between the two mapping types in every block (*p* < 0.001 for all comparisons). Consistent with H1, 1:2 mappings were more difficult to acquire than 1:1 mappings.

We then analysed accuracy in LP2 and examined performance differences across mapping type and block (H2a). Participants remapped words quite well (*M* = 0.79, *SD* = 0.41) and scored significantly above chance (chance level = 0.333, *t*(59) = 27.41, *p* < 0.001). We observed a significant main effect of former mapping type (β = 0.16, *SE* = 0.08, *z* = 1.97, *p* = 0.049) as well as a significant effect of block (β = 1.37, *SE* = 0.11,* z* = 12.98, *p* < 0.001). In line with our prediction,^2^ participants were generally more accurate with former 1:2 mappings (*M*_*1:2*_ = 0.80) compared to former 1:1 mappings (*M*_*1:1*_ = 0.78; see Fig. 3b). The interaction between former mapping type and block was not significant (*p* = 0.180).

Finally, we assessed accuracy in Block 1 of LP2 (H2b and H2c), where we found a significant main effect of former mapping type (β = 0.51, *SE* = 0.08, *z* = 4.96, *p* < 0.001). Consistent with our prediction (H2b), participants were more accurate in remapping former 1:2 mappings (*M*_*1:2*_ = 0.56) compared to former 1:1 mappings (*M*_*1:1*_ = 0.46). Additionally, as anticipated (H2c), the effect of former mapping type remained significant when adding accuracy in Block 5 of LP1 as a covariate (β = 0.41, *SE* = 0.17, *z* = 2.44, *p* = 0.015).

*Exploratory analyses.* We then examined RTs in LP1 and performance differences across mapping type and block (E1; means and SDs are summarised in Table 2). We found a significant main effect of mapping type (β = 0.33, *SE* = 0.02, *t* = 13.32, *p* < 0.001) as well as of block (β = −0.10, *SE* = 0.01, *t* = −12.14, *p* < 0.001). Participants were faster with 1:1 (*M*_*1:1*_ = 1795 ms) than with 1:2 (*M*_*1:2*_ = 2529 ms) mappings. We also found a significant interaction between mapping type and block (β = 0.05, *SE* = 0.01, *t* = 7.37, *p* < 0.001). Post hoc tests revealed a significant difference between the two mapping types in every block (*p* always < 0.001).

In LP2, we found a significant main effect of block (β = −0.04, *SE* = 0.01, *t* = −3.66, *p* < 0.001) and of former mapping type (β = −0.10, *SE* = 0.01, *t* =—8.01, *p* < 0.001). Participants were faster with former 1:2 (*M*_*1:2*_ = 1655 ms) than with former 1:1 (*M*_*1:1*_ = 1693 ms) mappings. Lastly, we examined RTs at the beginning of LP2 (E2b) and again found a main effect of former mapping type (β = −0.07, *SE* = 0.03, *t* = −2.49, *p* = 0.013). The effect remained significant when considering accuracy in Block 5 of phase 1 (E2c; β = −0.07, *SE* = 0.03, *t* = −2.53, *p* = 0.012). Participants were again faster with 1:2 (*M*_*1:2*_ = 2276 ms) than with 1:1 (*M*_*1:1*_ = 2410 ms) mappings.

*Between experiments analyses.* We conducted some additional, not pre-registered, analyses to understand differences across experiments better. Following the original analysis strategy, we added to the mixed model experiment as a factor (Experiments 1 and 2, contrast coded as + 0.5/−0.5). For accuracy, in LP1, we observed a significant three-way interaction between mapping type, block and experiment (β = 0.18, *SE* = 0.05, *z* = 3.76, *p* < 0.001). In particular, when the data were analysed separately for each experiment, we found a significant interaction of mapping type and block (*p* < 0.001). Participants were, in fact, always better with 1:1 than 1:2 mappings, and the difference between the two mapping types grows larger in later blocks, especially in Experiment 1. We also found significant main effects of mapping and block and interaction between the two, which were in line with the findings of the two experiments (the full results are reported in the between analyses part of the Online Supplementary Material).

In LP2, we found a significant interaction between former mapping type and experiment (β = 0.35, *SE* = 0.12, *z* = 2.85, *p* = 0.004), indicating a significant difference between former mapping types only in Experiment 2 (*p* = 0.035, *M*_*1:1*_ = 0.77, M_1:2_ = 0.79) and not in Experiment 1 (*p* = 0.390, *M*_*1:1*_ = 0.83, M_1:2_ = 0.81). The interaction between block and experiment was also significant (β = −0.07, *SE* = 0.03, *z* = −2.35, *p* = 0.019): In particular, there was a significant difference between the two experiments in the first two blocks (*p* < 0.05), indicating that participants were more accurate in the in-lab Experiment 1 (*M*_*exp1*_ = 0.56) than in the online Experiment 2 (*M*_*exp2*_ = 0.49). The interaction between former mapping type and block was also significant (β = 0.07, *SE* = 0.03, *z* = 2.04, *p* = 0.042). There was a significant difference between the two former mapping types only in Block 3 (*p* = 0.004), indicating that participants were more accurate with former 1:1 mappings (M_1:1_ = 0.88) than with former 1:2 (M_1:2_ = 0.86) mappings.

Lastly, for accuracy, we found a significant interaction between former mapping type and experiment (β = 0.44, *SE* = 0.15, *z* = 3.01, *p* = 0.003) in Block 1 of LP2. The two experiments were, in fact, not different for former 1:2 mappings (*p* = 0.844), but for former 1:1 ones (*p* < 0.001): In Experiment 1, participants remapped 1:1 mappings indeed better (*M*_*1:1*_ = 0.58) than in Experiment 2 (*M*_*1:1*_ = 0.46). A main effect of experiment was also found, indicating that participants were more accurate in Experiment 1 (*M*_*exp1*_ = 0.58) than in Experiment 2 (*M*_*exp2*_ = 0.49). The main effect of mapping was not significant (*p* = 0.105). However, interestingly, participants were generally more accurate with former 1:2 mappings (*M*_*1:2*_ = 0.55) than with former 1:1 ones (*M*_*1:1*_ = 0.51).

We then conducted the same analyses using RTs as DV. In LP1, we found a significant interaction of block and experiment (β = −0.04, *SE* = 0.01, *t* = −3.58, *p* < 0.001). In particular, participants were, in fact, significantly slower (*p* < 0.001) in Experiment 1 (*M*_*exp1*_ = 3,317 ms) than in Experiment 2 (*M*_*exp2*_ = 2,642 ms). We also found the main effects of mapping, block, and their interaction to align with the results of the two experiments.

In LP2, we found a significant interaction between former mapping type and experiment (β = −0.05, *SE* = 0.01, *t* = −4.07, *p* < 0.001). While in Experiment 2, there was a significant difference between the two former mapping types (*p* = 0.003, *M*_*1:1*_ = 1,698 ms, *M*_*1:2*_ = 1,657 ms), the difference was not significant in Experiment 1 (*p* = 0.090; and numerically in the opposite direction; *M*_*1:1*_ = 1,667 ms, *M*_*1:2*_ = 1,701 ms). Also, the interaction between block and experiment was significant (β = −0.04, *SE* = 0.02, *t* = −2.62, *p* = 0.001). In contrast to LP1, participants were slower in Experiment 2 (*M*_*exp2*_ = 2,638 ms) than in Experiment 1 (*M*_*exp1*_ = 2,628 ms), but only in the first block (*p* < 0.020). The main effect of block was also significant.

Regarding Block 1 of LP2, the interaction between experiment and former mapping type was significant (β = −0.08, *SE* = 0.04, *t* = −2.22, *p* = 0.026). While in Experiment 2, participants were faster with former 1:2 than with former 1:1 mappings (*p* = 0.003, *M*_*1:1*_ = 2425 ms, *M*_*1:2*_ = 2,271 ms), the difference between the two mapping types was not significant for Experiment 1 (*p* = 0.053).

### Discussion

In Experiment 2, we replicated better performance for 1:1 than 1:2 mappings for both accuracy and RTs. In LP2, in contrast, we found opposite results to those observed in Experiment 1 (but in the same line as the two pilots): 1:2 mappings were remapped more easily (and faster) not only in the first block but in all of LP2.

We compared the results of the two experiments in a between-experiment analysis. In LP1, we observed that the difference between 1:1 and 1:2 mappings grew larger at the end of this phase for Experiment 1 (compared to Experiment 2). In LP2, we found that in Experiment 2, the two mapping types were remapped differently (while in Experiment 1, they were not). Regardless of the mapping type, participants were more accurate in Experiment 1 than in Experiment 2. However, they were also slower. This finding could be explained by the different platforms on which the two experiments were conducted; that is, participants may be more motivated and/or less distracted in the in-lab setting (Experiment 1) than at home (Experiment 2). Similarly, Escudero and colleagues (2023) found better performance in one condition in the laboratory group compared to the online one in a CSWL experiment.

The performance difference across experiments could be due to a speed-accuracy trade-off (see Heitz, 2014 for a review). As described, participants were always more accurate but also slower in Experiment 1 compared to Experiment 2. Thus, it could be that in the in-lab experiment, for the completion of the experiment itself, participants were more interested in being accurate (on the expense of velocity) in contrast to participants online who were more interested in being fast (and therefore less accurate; Findlater et al., 2017, for similar findings; but see, Vékony et al., 2020, for evidence that statistical knowledge, when focusing on being fast vs. accurate, may not differ much).

Moreover, we found some interesting differences across experiments in Block 1 of LP2: Surprisingly, the two experiments differed in how *single* (and not multiple) mappings were remapped. Participants in Experiment 2 remapped 1:1 mappings worse than participants in Experiment 1 did. This finding could also help to explain why the results in Experiment 1 are so different from what we found in Experiment 2 (and in the pilots). In Experiment 1, participants seemed to be generally “too good” at remapping 1:1. This could be again due to the in-lab setting of this experiment; it seems that participants were paying “too” much attention and may have reached some type of ceiling performance.

Overall, the results of Experiment 2 suggest that multiple mappings are easier to remap, consistent with our predictions. However, the effects found in this experiment could be explained not only by the type of mappings but also by their frequency ( for examples of the effect of frequency on CSWL, see Chan & Monaghan, 2019; Kachergis et al., 2017;). That is, 1:2 mappings could be remapped more easily, not because they are intrinsically less robust but because they had less opportunity to be learnt. In Experiments 1 and 2, participants were exposed to eight 1:1 and only four 1:2 mappings as part of LP1. To see if the frequency of mappings can explain the observed learning differences between mapping types found in Experiments 1 and 2, we designed Experiment 3 with the same frequency of 1:1 and 1:2 mappings in LP1.

## Experiment 3

### Methods

#### Participants

Sixty-eight unbalanced German-English bilinguals participated online in Experiment 3, and 60 were included in the final sample (six participants had to be excluded because they did not reach 40% accuracy, one participant because they were older than 35 years, and one because their mother tongue was not German).

#### Materials and Procedure

The materials were the same as in Experiments 1 and 2. This experiment's procedure was identical to the one used in Experiment 2, as it was also conducted online via Gorilla Experiment Builder (Anwyl-Irvine et al., 2020). The only difference between Experiments 2 and 3 was the frequency of the two mapping types during LP1: In Experiment 3, there were six 1:1 and six 1:2 mappings; in contrast, in Experiments 1 and 2, there were eight 1:1 and four 1:2 mappings.

### Results

**Fig. 4:**
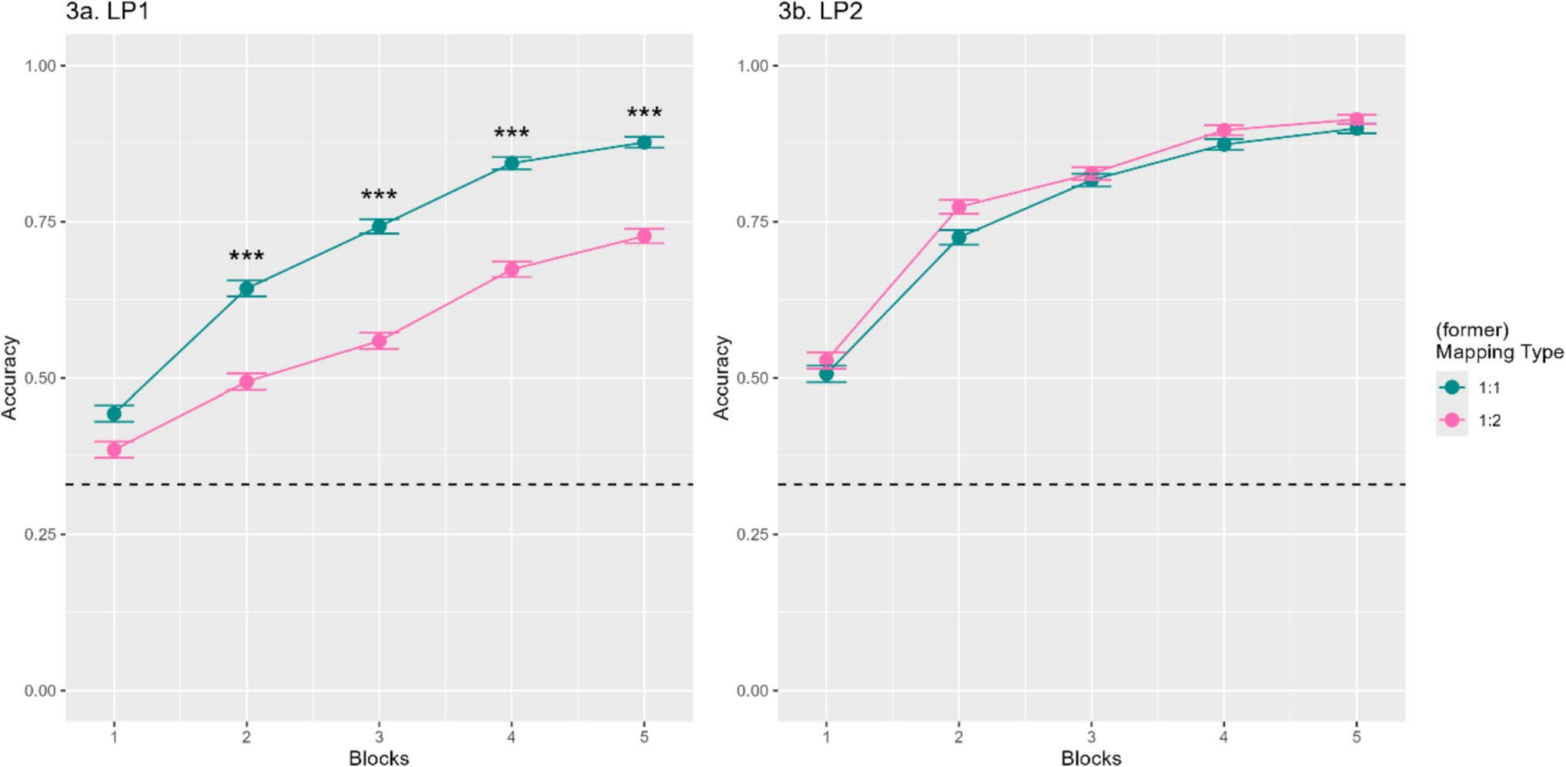
Accuracy performance in both phases of Experiment 3. **4a**. The left figure depicts accuracy performance in LP1 of Experiment 3 per mapping type (1:1, 1:2) and block (1–5). **4b**. The right figure depicts accuracy performance in LP2 of Experiment 2 per former mapping type (1:1, 1:2) and block (1–5). The dotted line indicates chance performance. Errors bars are the standard error of the mean. Asterisks (***) indicate significant differences between mean accuracy per (former) mapping type and block at *p* < 0.001

*Main analyses.* Again, we first examined accuracy in LP1 and performance differences across mapping types and blocks (H1). Participants’ learning was again quite good (*M* = 0.64, *SD* = 0.48), and they scored significantly above chance (chance level = 0.333, *t*(59) = 22.69, *p* < 0.001). We observed a significant main effect of mapping type (β = −1.08, *SE* = 0.23, *z* = −4.77, *p* < 0.001) and block (β = 0.59, *SE* = 0.04, *z* = 13.53, *p* < 0.001). As anticipated, participants exhibited higher accuracy with 1:1 mappings (*M*_*1:1*_ = 0.71) compared to 1:2 mappings (*M*_*1:2*_ = 0.57). Additionally, there was a significant interaction between mapping type and block (β = −0.32, *SE* = 0.03, *z* = −9.94, *p* < 0.001; see Fig. 4a). More specifically, there was a significant difference between the two mapping types in every block (*p* always < 0.001), except for Block 1 (*p* = 0.133). As before, 1:2 mappings were harder to acquire than 1:1 mappings consistent with H1.

We then analysed accuracy in LP2 and examined performance differences across former mapping type and block (H2a). Participants learned words quite effectively in the second phase (*M* = 0.78, *SD* = 0.42), scoring significantly above chance (chance level = 0.333, *t*(59) = 20.92, *p* < 0.001). We identified a significant main effect of former mapping type (β = 0.24, *SE* = 0.07, *z* = 3.46, *p* < 0.001) and block (β = 1.18, *SE* = 0.10, *z* = 11.73, *p* < 0.001). Overall, participants were more accurate with former 1:2 mappings (*M*_*1:2*_ = 0.79) compared to former 1:1 mappings (*M*_*1:1*_ = 0.76). The interaction between former mapping type and block was not significant (*p* = 0.212; see Fig. 4b). Against our pre-registered prediction (H2a) for Experiment 3 but consistent with the general idea that remapping may be facilitated for multiple mappings, 1:2 mappings were generally easier to remap than 1:1 ones.

Lastly, we examined accuracy in Block 1 of LP2 (H2b and H2c). We did not find a significant main effect of former mapping type (*p* = 0.214). Contrary to our prediction (H2b), while participants were numerically more accurate with former 1:2 mappings (*M*_*1:2*_ = 0.53) than with former 1:1 (*M*_*1:1*_ = 0.51) in Block 1 of LP2, this difference was not significantly different. Similarly, contrary to our prediction (H2c), we did not find a significant main effect of the former mapping type (*p* = 0.217) when the accuracy in Block 5 of LP1 was introduced as a covariate.

*Exploratory analyses.* As before, we first examined RTs in LP1 and performance differences across mapping type and block (E1; means and SDs are summarised in Table 2). We found a significant main effect of mapping type (β = 0.28, *SE* = 0.04, *t* = 7.62, *p* < 0.001) as well of block (β = −0.09, *SE* = 0.01, *t* = −10.78, *p* < 0.001). Participants were faster with 1:1 (*M*_*1:1*_ = 1,960 ms) than with 1:2 (*M*_*1:2*_ = 2,697 ms) mappings. We also found a significant interaction between mapping type and block (β = 0.08, *SE* = 0.01, *t* = 11.43, *p* < 0.001). There was a significant difference between the two mapping types in every block (*p* always < 0.020).

In LP2, we found only a significant main effect of block (E2a; β = −0.10, *SE* = 0.01, *t* = −10.91, *p* < 0.001). Lastly, we examined RTs in Block 1 of LP2 (E2b and E2c). We did not find a significant main effect of the former mapping type in both analyses.

*Between-experiments analyses.* We pre-registered to compare the results of Experiments 2 and 3, expecting to find no differences between experiments. We added to the mixed model experiment as a factor (Experiment 3 and 2, contrast coded as + 0.5/−0.5). Using accuracy as DV, against our prediction in LP1, we observed a significant main effect of experiment (β = −0.76, *SE* = 0.13, *z* = −5.86, *p* < 0.001); participants were, in fact, more accurate in Experiment 2 (*M*_*exp2*_ = 0.75) than in Experiment 3 (*M*_*exp3*_ = 0.62), consistent with a less difficulty of Experiment 2. We also found an interaction between block and experiment (β = −0.17, *SE* = 0.02, *z* = −8.04, *p* < 0.001). Specifically, the two experiments differed significantly in every block (*p* < 0.001); participants were always more accurate in Experiment 2 (from *M*_*1*_ = 0.48 to *M*_*5*_ = 0.89) than in Experiment 3 (from *M*_*1*_ = 0.41 to *M*_*5*_ = 0.77). Consistent with our hypothesis, the interaction of mapping type and experiment and the three-way interaction were not significant. Consistent with the findings of the two experiments, we also found a main effect of block and mapping type and their interaction (see Online Supplementary Materials for full results).

In LP2, we found a significant effect of block (β = 0.75, *SE* = 0.03, *z* = 28.83, *p* < 0.001) as well as an interaction between block and experiment (β = −0.23, *SE* = 0.03, *z* = −7.24, *p* < 0.001). Again, participants were generally more accurate in Experiment 2 than in Experiment 3 in every block but the first. However, the difference between the two experiments was significant only in the third block (*p* = 0.032; *M*_*exp2*_ = 0.86, *M*_*exp3*_ = 0.78).

Lastly, in Block 1 of LP2, we found a significant main effect of former mapping types (β = −0.23, *SE* = 0.11, *z* = −2.03, *p* = 0.043): Participants were generally more accurate with former 1:2 (*M*_*1:2*_ = 0.48) than with former 1:1 mappings (*M*_*1:1*_ = 0.53). We also found an interaction between former mapping type and experiment (β = 0.29, *SE* = 0.15, *z* = 1.99, *p* = 0.047). A significant difference between the two former mapping types was only present in Experiment 2 (*p* = 0.024, M_1:1_ = 0.46, M_1:2_ = 0.55) and not in Experiment 3 (*p* = 0.194).

Using RT as DV, in LP1, we did not find any significant main effect or interaction related to experiment, but again block, mapping type and their interaction were significant. In LP2, we instead found a significant main effect of block (β = −0.10, *SE* = 0.01, *t* = −13.35, *p* < 0.001), and an interaction of former mapping type and experiment (β = −0.03, *SE* = 0.01, *t* = −3.05, *p* = 0.002). Participants in Experiment 2 were, in fact, faster with former 1:1 (*p* = 0.003; *M*_*1:1*_ = 1,698 ms) than former 1:2 mappings (*M*_*1:2*_ = 1,657 ms), while they were equally fast for both former mapping types in Experiment 3 (*p* = 0.787). In block 1 of LP2, we did not find any significant effect.

### Discussion

As expected for LP1, 1:2 mappings were harder to learn than 1:1 mappings, replicating the observed pattern of Experiments 1 and 2. Notably, Experiment 3’s LP1 included an equal number of 1:1 and 1:2 mappings, suggesting that any observed performance pattern cannot be explained by base frequency solely. As such, our findings do not align with those of Chan and Monaghan (2019), where an increased number of multiple mappings made them easier to be learned than single ones. Instead, our results are consistent with Benitez et al. (2016) who found worse performance for multiple mappings than for single ones when those were presented at equal frequency. Regarding LP2, we could replicate Experiment 2’s finding that 1:2 mappings are easier to remap than 1:1 mappings. This suggests that the nature of 1:2 mappings, not their number, causes the difference in remapping ease across mapping types, consistent with our predictions. When we directly compared Experiments 2 and 3, we found that participants were generally less accurate in Experiment 3 than in Experiment 2 (in both phases). This finding aligned with the increased difficulty of Experiment 3 (but was against our pre-registered predictions), consistent with the higher number of multiple mappings present in this experiment. A generally better performance in Experiment 2 also aligns with the findings of Kachergis et al. (2017): In their experiment, participants learned better when there were different frequencies of words compared to the same (however, they only focused on single mappings).

Interestingly, we only found a significant effect of mapping when comparing the two experiments in Block 1 of LP2 (we did not find the difference in remapping 1:2 and 1:1 mappings in Experiment 3). Not finding any other interaction of experiment and mapping, neither in LP1 nor in LP2, indicates that while Experiment 3 was generally more complex, there was still a difference in how 1:1 and 1:2 mappings were learnt. Confirming that while multiple mappings were always more complicated to learn, they were still learnable and that the lower short-term frequency of multiple mappings could not explain the remapping effect found in Experiment 2. Finding a similar pattern in Experiment 3, even with a more difficult learning task, strengthens our confidence in the results of Experiment 2.

## General discussion

In a series of three experiments, we explored the flexibility of learning and remapping single and multiple word mappings (see Tables 3 and 4 for a summary of the results). In all three experiments, we confirmed that single mappings are learnt more easily than multiple mappings during initial acquisition, which aligns with the literature (e.g., Benitez et al., 2016; Poepsel & Weiss, 2016). In Experiment 3, we were able to rule out a possible influence of different local frequencies on this effect ( for a discussion on the effect of frequencies on CSWL, see Kachergis et al., 2017). However, we observed conflicting results for remapping: In Experiment 1 (conducted in the lab), single mappings appeared easier to remap, while the opposite was the case in Experiments 2 and 3 (conducted online).

**Table 3:** Summary of findings for accuracy of the three experiments

	Experiment 1				Experiment 2				Experiment 3			
	LP1		LP2		LP1		LP2		LP1		LP2	
Fixed Effects	β (SE)	z	β (SE)	z	β (SE)	z	β (SE)	z	β (SE)	z	β (SE)	z
Intercept	1.63 (0.14)	11.70***	3.39(0.25)	13.41***	1.66 (0.16)	10.43***	2.82 (0.27)	10.45***	0.87 (0.16)	5.36***	2.51 (0.27)	9.27***
(Former) mapping type	−1.25 (0.10)	−12.41***	−0.39 (0.07)	−5.59***	−1.12 (0.14)	−7.77***	0.16 (0.08)	1.97*	−1.08 (0.23)	−4.77***	0.24 (0.07)	3.46***
Block	0.86 (0.06)	15.39***	1.50 (0.12)	13.04***	0.86 (0.06)	14.12***	1.37 (0.11)	12.98***	0.59 (0.04)	13.53***	1.18 (0.10)	11.73***
Interaction	−0.44 (0.04)	−11.75***	−0.15 (0.04)	−3.37***	−0.24 (0.04)	−6.34***	−0.06 (0.04)	−1.34	−0.32 (0.03)	−9.94***	0.05 (0.04)	1.25
	Experiment 1				Experiment 2				Experiment 3			
	LP2 – Block 1				LP2 – Block 1				LP2 – Block 1			
Fixed Effects	β (SE)		z		β (SE)		z		β (SE)		z	
Intercept	0.30 (0.07)		4.205***		0.04 (0.08)		0.58		0.11 (0.13)		0.79	
Former mapping type	−0.10 (0.08)		−1.26		0.41 (0.08)		4.96***		0.15 (0.12)		1.24	

We used linear mixed models for our hypotheses in all experiments. We contrast coded (former) mapping type (1:1, 1:2; contrast coded as + 0.5/- 0.5) and centred block (1–5). As random effects, we always considered participant, word, and target object. The best model was chosen using the function buildmer in the buildmer package (v. 2.8, Voeten & Voeten, 2021). The specific models for each analysis are contained in the supplementary materials. We tested all our hypotheses using general linear mixed models using the package lme4 (Bates et al., 2015), with accuracy (1/0) as the DV.

* *p* < 0.05.

** *p* < 0.01.

*** *p* < 0.001.

**Table 4:** Summary of findings for reaction times (RTs) of the three experiments

	Experiment 1				Experiment 2				Experiment 3			
	LP1		LP2		LP1		LP2		LP1		LP2	
Fixed Effects	β (SE)	t	β (SE)	t	β (SE)	t	β (SE)	t	β (SE)	t	β (SE)	t
Intercept	7.63 (0.04)	214.70***	7.31 (0.03)	266.11***	7.55 (0.03)	261.01***	7.25 (0.05)	143.15***	7.63 (0.03)	223.18***	7.32 (0.27)	161.32***
(Former) mapping type	0.36 (0.02)	14.42***	0.02 (0.02)	1.14	0.33 (0.02)	13.33***	−0.04 (0.01)	−3.66***	0.28 (0.04)	7.62***	−0.03 (0.02)	−1.88
Block	−0.14 (0.01)	−23.35***	−0.15 (0.01)	−16.20***	−0.10 (0.01)	−12.14***	−0.10 (0.01)	−8.01***	−0.09 (0.01)	−10.78***	−0.10 (0.01)	−10.91***
Interaction	0.45 (0.01)	7.24***	−0.01 (0.01)	−1.48	0.05 (0.01)	7.37***	0.00 (0.01)	0.67	−0.08 (0.01)	11.43***	−0.01 (0.01)	0.91
	Experiment 1				Experiment 2				Experiment 3			
	LP2 – Block 1				LP2 – Block 1				LP2 – Block 1			
Fixed Effects	β (SE)		t		β (SE)		t		β (SE)		t	
Intercept	7.75 (0.03)		254.65***		7.54 (0.06)		132.37***		7.61 (0.05)		151.60***	
Former mapping type	0.02 (0.03)		0.69		−0.07 (0.03)		−2.49*		0.00 (0.02)		0.07	

We used linear mixed models for our hypotheses in all experiments. We contrast coded (former) mapping type (1:1, 1:2; contrast coded as + 0.5/- 0.5) and centred block (1–5). As random effects, we always considered participant, word, and target object. The best model was chosen using the function buildmer in the buildmer package (v. 2.8, Voeten & Voeten, 2021). The specific models for each analysis are contained in the supplementary materials. We tested all our hypotheses using linear mixed models using the package lme4 (Bates et al., 2015), with RTs (transformed via logarithmic scale) as the DV

* *p* < 0.05

** *p* < 0.01

*** *p* < 0.001

Concerning the learning phase, we replicated previous findings in a bilingual population. Despite the idea that bilinguals may be more open to acquiring multiple mappings than monolinguals due to being exposed to them more frequently long-term (Poepsel & Weiss, 2016; but see Aguasvivas et al., 2024), we were still able to see a clear performance benefit for single mappings compared with multiple ones. Thus, we can endorse the idea that a global frequency (i.e., being bilinguals) is not enough to make multiple mappings the learning rule (at least in the case of unbalanced bilinguals as tested here). Future experiments should try to further increase the frequency of multiple mappings compared to single ones (as done by Chan & Monaghan, 2019) to see if, in this case, it is possible to make them easier to learn. It is important to note that, even if we discuss them as multiple mappings, this series of experiments focused only on 1:2 mappings (similar to Poepsel & Weiss, 2016). Therefore, it would also be interesting to investigate if it is possible to replicate the same findings with different types of multiple mappings (like 2:1 or for example 1:3).

Regarding local differences in mapping frequencies, we could observe that while they do impact learning difficulty in CSWL, they do not explain how easily it is to remap a word’s meaning. In particular, by comparing Experiments 2 and 3, we could assess a more general difficulty of Experiment 3, but still observe a higher easiness for single mappings (in contrast with Chan and Monaghan’s (2019) findings). This again indicates that for our unbalanced bilinguals a higher local frequency of multiple mappings – which theoretically may be more in line with their experienced global frequency – did not facilitate learning. At this point, not many papers have devoted attention to different frequencies and multiple mappings (but see Vouloumanos, 2008). In future research, it could be interesting to follow a more naturalistic approach to frequency, like the Zipfian distribution (i.e., some words are far more likely to be encountered than others Piantadosi, 2014; Zipf, 1949). As Hendrickson and Perfors (2019) suggested, learning in CSWL is boosted in a Zipfian distribution. Following this logic, one could then recreate a distribution of mappings as close as possible to the one bilinguals experience in real life, though it is not clear how the exact frequency distribution of different mapping types for bilinguals could be estimated.

While the results were clear in the learning phase, we found conflicting ones in the remapping phase: In Experiment 1 (in-lab), single mappings seemed easier to remap. In contrast, the other two (online) experiments found that multiple mappings were easier to remap. Conducting between experiments analyses, we found some differences regarding the first block of the remapping phase, suggesting that participants in Experiment 1 remapped mappings with single meanings more easily than participants in Experiment 2 (while absolute performance for multiple mappings was comparable across experiments). Several factors could explain these differences, including the variation in platforms on which the experiments were conducted. More specifically, it is possible that participants learned better in the in-lab setting due to higher motivation or less distraction than when being tested online (e.g., Clifford & Jerit, 2014). Differences in learning strategy were also mirrored in the speed-accuracy trade-off, where in-lab participants were more accurate but slower, while online participants were less accurate but faster. Such a speed-accuracy trade-off may be an indication that attention is allocated differently during word learning across platforms, with a higher focus on accuracy leading to better learning and retention in LP1 (Rothwell et al., 2024).

Attention has been considered an important predictor of statistical learning (Aslin, 2017; see for example, Baker et al., 2004; and Toro et al., 2005), and differences in how attention is allocated have been found to affect CSWL in previous studies (e.g., Yu et al., 2012). Online experimentation has increased significantly in recent years (Gentili & Cristea, 2020), though this transition has raised concerns (Grootswagers, 2020). To our knowledge, only one other study has compared online and in-lab statistical word learning using a CSWL paradigm (Escudero et al., 2023), where online participants were monitored via an experimenter during participation (which, of course, was not the case in our study). In their case, they found better results in the lab compared to online in the condition where words were presented in written form (as in our study) but similar performance for the condition where words were presented auditorily. Together with ours, these findings suggest that specifically word learning may be harder online when many visual elements have to be attended to concurrently (e.g., potentially due to visual crowding on smaller laptop screens). Overall, the influence of platform differences on attention and (statistical) word learning patterns deserves further exploration. In this context, it must be noted that both pilot experiments were conducted online on which we based the pre-registrations. Since few studies have compared online and in-lab experiments, we did not account for the potential impact of changing platforms between the pilots and Experiment 1. However, future research should keep constant the modality between pilots and actual experiments.

Another possible contributor to the differences between Experiment 1 and 2 is the great variability in word learning studies, especially with CSWL. As Roembke et al. (2023) pointed out, there is significant variability at the individual level and very few studies on individual differences (e.g., Bhat et al., 2022; Smith & Yu, 2013; Yu & Smith, 2011). There are also very few cases of replication in this field, and most of the time with conflicting results (see, for example, Poepsel & Weiss, 2016, and the replications by Aguasvivas et al., 2024, and Tachakourt & Wang, 2023). Prior research with a different statistical learning paradigm has indicated that performance may vary based on participants’ academic backgrounds (Kim et al., 2023) and literacy (Zinszer et al., 2024). Even though our participants were matched across experiments on most of the variables we explored (e.g., gender, education, see Table 2), samples were not matched for variables such as intelligence (as done for instance in the CSWL study on children by Crespo et al., 2023), working memory capacity (when comparing CSWL in monolinguals and bilinguals: Neveu & Kaushanskaya, 2024), or socioeconomic status (again children: Crespo et al., 2023), as is typical for experimental studies in adults where no between-subject comparisons are the focus and where participants are generally drawn from the same demographic pool. However, participants were slightly mismatched in their self-reported L2 proficiency for reading and understanding; this was true for the direct comparison of Experiments 1 and 2 only (and it was not supported by the LexTALE scores). Thus, it is possible that differences in the participants’ characteristics of Experiments 1 and 2 led to the observed differences in remapping patterns. In CSWL, people’s language history has been investigated as a factor that may impact performance, with mixed results (e.g., research on whether the bilingual word learning advantage exists in CSWL: Aguasvivas et al., 2024; Benitez et al., 2016; Poepsel & Weiss, 2016). At this point, there is no evidence that language history within the broader category of bilinguals relates to how words or different mapping types are learned cross-situationally (Simonetti et al., Manuscript submitted for publication).

To the best of our knowledge, only one other study (Bulgarelli et al., 2021) has explored the remapping of word meanings in CSWL, testing remapping differences between younger and older adults. In Bulgarelli et al.’s (2021) Experiment 2, younger and older monolinguals first experienced 1:1 mappings. Then, after this familiarisation phase, some objects received a second label (thus creating 2:1 mappings). Monolinguals were found to struggle to remap objects in the second phase. These results contradict the findings of Benitez and colleagues (2016), who found that both monolinguals and bilinguals were able to acquire multiple mappings. Nevertheless, Bulgarelli et al.’s (2021) study shows the feasibility of using remapping in the context of CSWL to measure how mappings are encoded. Based on our data, we can conclude that multiple mappings are easier to remap than single ones (though how attention is allocated may change remapping processes; see Experiment 1). This finding is consistent with the idea that weaker connections are created when learning multiple mappings than those formed with single mappings. These weaker connections may cause less proactive interference (Anderson, 1992; Shiffrin, 1999), meaning that the strength and not the number of connections determine the level of interference. At the same time, the more robust representation that is part of a 1:1 mapping can then inhibit or block the competing weaker representation (Yurovsky et al., 2013). Remapping is especially relevant in the context of bilingualism, as each time a bilingual individual learns a word in a new language, they are effectively remapping existing associations. Our findings suggest a possible mechanism for why bilinguals may be more prone than monolinguals to learn a third language (Abu-Rabia & Sanitsky, 2010; Bartolotti & Marian, 2012; Cenoz, 2013; Singh, 2018).

Inconsistent with this interpretation, in Experiment 2, we could see a direct effect of accuracy at the end of LP1 on remapping (suggesting varying levels of robustness for the different mappings types). In contrast, this effect was not found in Experiment 3. Despite this, remapping was better for multiple mappings than single ones in both experiments. One possible reason for this data pattern is that accuracy at the end of LP1 was operationalised as the averaged overall accuracy during its final block; thus, this covariate was not word-specific and could have been too general to capture the relationship between accuracy at the end of LP1 and remapping performance in LP2. Thus, one open question refers to what would happen if participants had always reached the same level of accuracy for words with multiple meanings compared to words with single meanings (e.g., by using a set-up where participants are taught to criterion).

Our results also generally align with the idea that bilinguals make less use of the mutual exclusivity bias (Houston‐Price et al., 2010), and, interestingly, that they can do so specifically for some words: That is, the greater ease in remapping multiple mappings could be a hint that mutual exclusivity was reduced for these objects, making participants more prone to learning a third meaning. However, to make claims about differences in mutual exclusivity bias across populations, we would need a monolingual comparison group to assess monolinguals' greater use of mutual exclusivity bias. One could speculate that monolinguals, due to adhering more to mutual exclusivity during word learning (which again is a result of the lower long-term frequency of multiple mappings for this group; Kalashnikova et al., 2015), would struggle more with the acquisition of the multiple mappings (Poepsel & Weiss, 2016; but see Simonetti et al., Manuscript submitted for publication, for evidence against this hypothesis) and remapping overall. It is less clear whether remapping words with multiple meanings vs. words with only one meaning should differ across language groups: If what drives this remapping difference across mapping types for bilinguals is the strength of proactive interference between words and referents within a training session (c.f., Fig. 1), this specific cognitive process may not actually differ across language groups. Future research could investigate this question.

During these three experiments, we also explored RTs in the context of statistical word learning. Not many CSWL experiments have considered this measure (e.g., Escudero et al., 2016; Ge et al., 2024) in favour of the more straightforward accuracy. RTs can be used as indicators of how well a word can be learned and how quickly a meaning can be retrieved. As such, they are useful in corroborating our hypotheses. In the learning phase, RT analyses consistently aligned with accuracy ones, showing significant results in the expected direction: That is, 1:2 mappings were consistently more slowly selected than 1:1 mappings, even at the end of the learning phase where participants’ accuracy was generally high. This is consistent with the notion that the retrieval of the correct target was harder for multiple mappings, likely due to competition with the second (non-present) target. However, a more nuanced pattern emerged during the remapping phase. In Experiment 2, participants were more accurate and faster at remapping single mappings than multiple ones. In contrast, participants in Experiment 1 were more accurate in remapping single mappings but showed no corresponding increase in speed. A similar pattern appeared in Experiment 3, where participants were more accurate in remapping multiple mappings but did not show faster response times. The finding that RT did not consistently differ for former single and multiple mappings during remapping may indicate lower levels of competition during retrieval in LP2, potentially because participants had to always acquire 1:1 mappings at that point. Overall, the pattern of results indicates that RT could be used as an additional, complementary measure of retrieval ease in CSWL experiments.

## Conclusion

In conclusion, this series of pre-registered experiments explored the learning and remapping of words with single and multiple meanings. While single mappings were consistently easier to learn, we found conflicting results in the remapping phase: In-lab participants remapped single mappings more easily, while multiple mappings were easier to remap in the online experiments, independently of their local frequency. This suggests that while words with multiple meanings are less robustly encoded, they may remain more flexible when adding additional meanings at a later time point, at least under online testing conditions. Additionally, our exploration of RTs as a secondary performance measure in statistical word learning indicates that it may be sensitive to in-the-moment retrieval difficulty.

## Supplementary Information

Below is the link to the electronic supplementary material.

Supplementary file1 (DOCX 1327 KB)

## Data Availability

The raw, anonymised datasets are freely available on https://osf.io/7zw4s/?view_only=36a7ea26c81647d8b116efe72c921f74 All three experiments were pre-registered: - Experiment 1 https://doi.org/10.23668/psycharchives.12909 - Experiment 2 https://doi.org/10.23668/psycharchives.14033 - Experiment 3 https://doi.org/10.23668/psycharchives.14479.

## Footnotes

1 The pilot studies were conducted online with 42 and 25 participants, respectively. In both experiments, we found a significant main effect of mapping types in the first block of LP2, whilst we did not find a general main effect of mapping type during all of LP2. The main difference between the pilots and the current experiments is that in the former, the word was only shown very briefly (300 ms). In the pilots, we found many non-learners, which we speculated to be the result of the word’s short exposure time.

2 We did not pre-register a main effect of former mapping type, as we expected it not to be strong enough to reach significance. However, the main effect of mapping type, as observed, is consistent with our overall hypothesis that 1:2 mappings are easier to remap than 1:1 ones.

## Code availability

The custom R code used for the current study is freely available on https://osf.io/7zw4s/?view_only=36a7ea26c81647d8b116efe72c921f74.
