# Supplementary material for: How do multiple meanings affect word learning and remapping?
Source: Mem Cognit. 2025 Mar 24;53(7):2197–216. doi: 10.3758/s13421-025-01706-z (PMC12589246; doi:10.3758/s13421-025-01706-z)
Supplement: Supplementary file 1 — Supplementary file1 (DOCX 1327 KB) [file 13421_2025_1706_MOESM1_ESM.docx]

**Supplementary Materials**

**Demographics**

**ANOVAs**

**Age**

Formula: age ~ exp

|  | Df | Sum Sq | Mean Sq | F value | Pr(>F) |
| --- | --- | --- | --- | --- | --- |
| exp | 2 | 62.2 | 31.09 | 2.152 | 0.119 |
| Residuals | 180 | 2599.6 | 14.44 |  |  |

**English LexTALE**

Formula: lextale_E ~ exp

|  | Df | Sum Sq | Mean Sq | F value | Pr(>F) |
| --- | --- | --- | --- | --- | --- |
| exp | 2 | 0.0058 | 0.002925 | 0.243 | 0.785 |
| Residuals | 180 | 2.1696 | 0.012053 |  |  |

**German LexTALE**

Formula: lextale_G ~ exp

|  | Df | Sum Sq | Mean Sq | F value | Pr(>F) |
| --- | --- | --- | --- | --- | --- |
| exp | 2 | 0.0107 | 0.005345 | 1.404 | 0.248 |
| Residuals | 180 | 0.6855 | 0.003808 |  |  |

**Age of Acquisition**

Formula: L2_age_aquisition ~ exp

|  | Df | Sum Sq | Mean Sq | F value | Pr(>F) |
| --- | --- | --- | --- | --- | --- |
| exp | 2 | 12.7 | 6.345 | 1.497 | 0.227 |
| Residuals | 154 | 652.9 | 4.239 |  |  |

**Proficiency Speaking L2**

Formula: L2_proficiency_speaking~ exp

|  | Df | Sum Sq | Mean Sq | F value | Pr(>F) |
| --- | --- | --- | --- | --- | --- |
| exp | 2 | 0.46 | 0.2288 | 0.229 | 0.795 |
| Residuals | 153 | 152.69 | 0.998 |  |  |

**Proficiency Understanding L2**

Formula: L2_proficiency_understanding ~ exp

|  | Df | Sum Sq | Mean Sq | F value | Pr(>F) |
| --- | --- | --- | --- | --- | --- |
| exp | 2 | 7.28 | 3.64 | 3.902 | 0.0222* |
| Residuals | 154 | 143.66 | 0.933 |  |  |

Tukey’s HSD

| $exp | diff | lwr | upr | p adj |
| --- | --- | --- | --- | --- |
| Exp 2-Exp 1 | -0.53849 | -0.99848 | -0.0785 | 0.017197* |
| Exp 3-Exp 1 | -0.36669 | -0.83017 | 0.09678 | 0.150188 |
| Exp 3-Exp 2 | 0.171798 | -0.25644 | 0.600032 | 0.609912 |

**Proficiency Reading L2**

Formula: L2_proficiency_reading ~ exp

|  | Df | Sum Sq | Mean Sq | F value | Pr(>F) |
| --- | --- | --- | --- | --- | --- |
| exp | 2 | 9.98 | 4.989 | 4.78 | 0.00968** |
| Residuals | 154 | 160.71 | 1.044 |  |  |

Tukey’s HSD

| $exp | diff | lwr | upr | p adj |
| --- | --- | --- | --- | --- |
| Exp 2-Exp 1 | -0.6247 | -1.11122 | -0.13818 | 0.007819** |
| Exp 3-Exp 1 | -0.45598 | -0.94618 | 0.034222 | 0.074114 |
| Exp 3-Exp 2 | 0.168719 | -0.28421 | 0.621648 | 0.652731 |

**Fisher’s tests**

**Gender**

p-value = 0.2333

alternative hypothesis: two.sided

**Education**

p-value = 0.1192

alternative hypothesis: two.sided

**Experiment 1**

**RESULTS**

**H1**

Formula: accuracy ~ 1 + mapping_type_cc + block_s + mapping_type_cc:block_s +

(1 + block_s + mapping_type_cc | subject) + (1 + block_s | word) + (1 | target)

Data: phase1

| AIC | BIC | logLik | deviance | df.resid |
| --- | --- | --- | --- | --- |
| 12952.5 | 13059 | -6462.2 | 12924.5 | 14866 |

Scaled residuals:

| Min | 1Q | Median | 3Q | Max |
| --- | --- | --- | --- | --- |
| -19.4987 | 0.0281 | 0.2398 | 0.546 | 1.9063 |

Random effects:

| Groups | Name | Variance | Std.Dev. | Corr |  |
| --- | --- | --- | --- | --- | --- |
| subject | (Intercept) | 0.828766 | 0.9104 |  |  |
|  | block_s | 0.150626 | 0.3881 | 0.9 |  |
|  | mapping_type_cc | 0.433786 | 0.6586 | -0.42 | -0.26 |
| target | (Intercept) | 0.040543 | 0.2014 |  |  |
| word | (Intercept) | 0.041779 | 0.2044 |  |  |
|  | block_s | 0.002693 | 0.0519 | 0.98 |  |

Number of obs: 14880, groups: subject, 62; target, 28; word, 12

Fixed effects:

|  | Estimate | Std. Error | z value | Pr(>\|z\|) |  |
| --- | --- | --- | --- | --- | --- |
| (Intercept) | 1.63139 | 0.1395 | 11.7 | <2e-16 | *** |
| mapping_type_cc | -1.25486 | 0.10115 | -12.41 | <2e-16 | *** |
| block_s | 0.86461 | 0.05619 | 15.39 | <2e-16 | *** |
| mapping_type_cc:block_s | -0.44085 | 0.03753 | -11.75 | <2e-16 | *** |

---

Signif. codes: 0 ‘***’ 0.001 ‘**’ 0.01 ‘*’ 0.05 ‘.’ 0.1 ‘ ’ 1

Correlation of Fixed Effects:

|  | (Intr) | mppn__ | blck_s |
| --- | --- | --- | --- |
| mppng_typ_c | -0.314 |  |  |
| block_s | 0.829 | -0.239 |  |
| mppng_ty_:_ | -0.051 | 0.286 | -0.03 |

**Post – hoc (Mapping type * Block)**

**Block 1:**

Generalized linear mixed model fit by maximum likelihood (Laplace Approximation) ['glmerMod']

Family: binomial ( logit )

Formula: accuracy ~ 1 + mapping_type_cc + (1 + mapping_type_cc | subject) + (1 | target)

Data: get(lp_var)

| AIC | BIC | logLik | deviance | df.resid |
| --- | --- | --- | --- | --- |
| 4028.5 | 4064.5 | -2008.3 | 4016.5 | 2970 |

Scaled residuals:

| Min | 1Q | Median | 3Q | Max |
| --- | --- | --- | --- | --- |
| -1.8365 | -0.866 | -0.6809 | 0.9954 | 1.6447 |

Random effects:

| Groups | Name | Variance | Std.Dev. | Corr |  |  |  |
| --- | --- | --- | --- | --- | --- | --- | --- |
| subject | (Intercept) | 0.13689 | 0.37 |  |  |  |  |
|  | mapping_type_cc | 0.23915 | 0.489 | -0.66 |  |  |  |
| target | (Intercept) | 0.05582 | 0.2363 |  |  |  |  |

Number of obs: 2976, groups: subject, 62; target, 28

Fixed effects:

|  | Estimate | Std. Error | z value | Pr(>\|z\|) |  |
| --- | --- | --- | --- | --- | --- |
| (Intercept) | -0.1948 | 0.07644 | -2.549 | 0.01082 | * |
| mapping_type_cc | -0.32857 | 0.10238 | -3.209 | 0.00133 | ** |

---

Signif. codes: 0 ‘***’ 0.001 ‘**’ 0.01 ‘*’ 0.05 ‘.’ 0.1 ‘ ’ 1

Correlation of Fixed Effects:

(Intr)

mppng_typ_c -0.109

**Block 2:**

Generalized linear mixed model fit by maximum likelihood (Laplace Approximation) ['glmerMod']

Family: binomial ( logit )

Formula: accuracy ~ 1 + mapping_type_cc + (1 + mapping_type_cc | subject)

+ (1 + mapping_type_cc | word)

Data: get(lp_var)

| AIC | BIC | logLik | deviance | df.resid |
| --- | --- | --- | --- | --- |
| 3347.6 | 3395.6 | -1665.8 | 3331.6 | 2968 |

Scaled residuals:

| Min | 1Q | Median | 3Q | Max |
| --- | --- | --- | --- | --- |
| -4.3817 | -1.0037 | 0.4217 | 0.6779 | 1.4057 |

Random effects:

| Groups | Name | Variance | Std.Dev. | Corr |
| --- | --- | --- | --- | --- |
| subject | (Intercept) | 0.41103 | 0.6411 |  |
|  | mapping_type_cc | 0.38713 | 0.6222 | -0.83 |
| word | (Intercept) | 0.01079 | 0.1039 |  |
|  | mapping_type_cc | 0.17599 | 0.4195 | -0.69 |

Number of obs: 2976, groups: subject, 62; word, 12

Fixed effects:

|  | Estimate | Std. Error | z value | Pr(>\|z\|) |  |
| --- | --- | --- | --- | --- | --- |
| (Intercept) | 0.90794 | 0.09842 | 9.225 | < 2e-16 | *** |
| mapping_type_cc | -0.98972 | 0.17168 | -5.765 | 8.17E-09 | *** |

---

Signif. codes: 0 ‘***’ 0.001 ‘**’ 0.01 ‘*’ 0.05 ‘.’ 0.1 ‘ ’ 1

Correlation of Fixed Effects:

(Intr)

mppng_typ_c -0.455

**Block 3:**

Generalized linear mixed model fit by maximum likelihood (Laplace Approximation) ['glmerMod']

Family: binomial ( logit )

Formula: accuracy ~ 1 + mapping_type_cc + (1 + mapping_type_cc | subject)

Data: get(lp_var)

| AIC | BIC | logLik | deviance | df.resid |
| --- | --- | --- | --- | --- |
| 2525 | 2555 | -1257.5 | 2515 | 2971 |

Scaled residuals:

| Min | 1Q | Median | 3Q | Max |
| --- | --- | --- | --- | --- |
| -6.2902 | 0.159 | 0.3393 | 0.4723 | 1.3074 |

Random effects:

| Groups | Name | Variance | Std.Dev. | Corr |
| --- | --- | --- | --- | --- |
| subject | (Intercept) | 0.78 | 0.8832 |  |
|  | mapping_type_cc | 0.1165 | 0.3414 | -0.96 |

Number of obs: 2976, groups: subject, 62

Fixed effects:

|  | Estimate | Std. Error | z value | Pr(>\|z\|) |  |
| --- | --- | --- | --- | --- | --- |
| (Intercept) | 1.6566 | 0.1281 | 12.93 | <2e-16 | *** |
| mapping_type_cc | -1.3709 | 0.1285 | -10.67 | <2e-16 | *** |

---

Signif. codes: 0 ‘***’ 0.001 ‘**’ 0.01 ‘*’ 0.05 ‘.’ 0.1 ‘ ’ 1

Correlation of Fixed Effects:

(Intr)

mppng_typ_c -0.377

**Block 4:**

Generalized linear mixed model fit by maximum likelihood (Laplace Approximation) ['glmerMod']

Family: binomial ( logit )

Formula: accuracy ~ 1 + mapping_type_cc + (1 + mapping_type_cc | subject) + (1 | word)

Data: get(lp_var)

| AIC | BIC | logLik | deviance | df.resid |
| --- | --- | --- | --- | --- |
| 1773.8 | 1809.8 | -880.9 | 1761.8 | 2970 |

Scaled residuals:

| Min | 1Q | Median | 3Q | Max |
| --- | --- | --- | --- | --- |
| -7.327 | 0.0833 | 0.197 | 0.3601 | 1.2755 |

Random effects:

| Groups | Name | Variance | Std.Dev. | Corr |
| --- | --- | --- | --- | --- |
| subject | (Intercept) | 1.70662 | 1.3064 |  |
|  | mapping_type_cc | 1.68343 | 1.2975 | -0.65 |
| word | (Intercept) | 0.08162 | 0.2857 |  |

Number of obs: 2976, groups: subject, 62; word, 12

Fixed effects:

|  | Estimate | Std. Error | z value | Pr(>\|z\|) |  |
| --- | --- | --- | --- | --- | --- |
| (Intercept) | 2.7079 | 0.2256 | 12.003 | < 2e-16 | *** |
| mapping_type_cc | -2.0762 | 0.3003 | -6.914 | 4.72E-12 | *** |

---

Signif. codes: 0 ‘***’ 0.001 ‘**’ 0.01 ‘*’ 0.05 ‘.’ 0.1 ‘ ’ 1

Correlation of Fixed Effects:

(Intr)

mppng_typ_c -0.570

**Block 5:**

Generalized linear mixed model fit by maximum likelihood (Laplace Approximation) ['glmerMod']

Family: binomial ( logit )

Formula: accuracy ~ 1 + mapping_type_cc + (1 + mapping_type_cc | subject) +

(1 + mapping_type_cc | word)

Data: get(lp_var)

| AIC | BIC | logLik | deviance | df.resid |
| --- | --- | --- | --- | --- |
| 1540.7 | 1588.6 | -762.3 | 1524.7 | 2968 |

Scaled residuals:

| Min | 1Q | Median | 3Q | Max |
| --- | --- | --- | --- | --- |
| -8.5474 | 0.0584 | 0.1538 | 0.3218 | 1.1979 |

Random effects:

| Groups | Name | Variance | Std.Dev. | Corr |
| --- | --- | --- | --- | --- |
| subject | (Intercept) | 2.1707 | 1.4733 |  |
|  | mapping_type_cc | 1.32 | 1.1489 | -0.96 |
| word | (Intercept) | 0.1095 | 0.331 |  |
|  | mapping_type_cc | 0.4485 | 0.6697 | -0.64 |

Number of obs: 2976, groups: subject, 62; word, 12

Fixed effects:

|  | Estimate | Std. Error | z value | Pr(>\|z\|) |  |
| --- | --- | --- | --- | --- | --- |
| (Intercept) | 3.1076 | 0.2691 | 11.55 | < 2e-16 | *** |
| mapping_type_cc | -2.6523 | 0.4039 | -6.567 | 5.12E-11 | *** |

---

Signif. codes: 0 ‘***’ 0.001 ‘**’ 0.01 ‘*’ 0.05 ‘.’ 0.1 ‘ ’ 1

Correlation of Fixed Effects:

(Intr)

mppng_typ_c -0.737

**H2a:**

Formula: accuracy ~ 1 + mapping_type_cc + block_s + mapping_type_cc:block_s +

(1 + block_s | subject) + (1 | word)

Data: phase2

| AIC | BIC | logLik | deviance | df.resid |
| --- | --- | --- | --- | --- |
| 9130.8 | 9191.7 | -4557.4 | 9114.8 | 14872 |

Scaled residuals:

| Min | 1Q | Median | 3Q | Max |
| --- | --- | --- | --- | --- |
| -37.463 | 0.02 | 0.124 | 0.366 | 1.981 |

Random effects:

| Groups | Name | Variance | Std.Dev. | Corr |
| --- | --- | --- | --- | --- |
| subject | (Intercept) | 3.32952 | 1.8247 |  |
|  | block_s | 0.66157 | 0.8134 | 0.97 |
| word | (Intercept) | 0.02309 | 0.152 |  |

Number of obs: 14880, groups: subject, 62; word, 12

Fixed effects:

|  | Estimate | Std. Error | z value | Pr(>\|z\|) |  |
| --- | --- | --- | --- | --- | --- |
| (Intercept) | 3.38672 | 0.25252 | 13.412 | < 2e-16 | *** |
| mapping_type_cc | -0.39164 | 0.07001 | -5.594 | 2.21E-08 | *** |
| block_s | 1.49938 | 0.115 | 13.038 | < 2e-16 | *** |
| mapping_type_cc:block_s | -0.1509 | 0.04476 | -3.372 | 0.000748 | *** |

---

Signif. codes: 0 ‘***’ 0.001 ‘**’ 0.01 ‘*’ 0.05 ‘.’ 0.1 ‘ ’ 1

Correlation of Fixed Effects:

|  | (Intr) | mppn__ | blck_s |
| --- | --- | --- | --- |
| mppng_typ_c | 0.024 |  |  |
| block_s | 0.942 | 0.013 |  |
| mppng_ty_:_ | 0.014 | 0.614 | 0.046 |

**Post – hoc (Mapping type * Block)**

**Block 1:**

Generalized linear mixed model fit by maximum likelihood (Laplace Approximation) ['glmerMod']

Family: binomial ( logit )

Formula: accuracy ~ 1 + mapping_type_cc + (1 + mapping_type_cc | subject)

Data: get(lp_var)

| AIC | BIC | logLik | deviance | df.resid |
| --- | --- | --- | --- | --- |
| 3981.7 | 4011.7 | -1985.8 | 3971.7 | 2971 |

Scaled residuals:

| Min | 1Q | Median | 3Q | Max |
| --- | --- | --- | --- | --- |
| -1.8914 | -1.0434 | 0.6156 | 0.8343 | 1.4772 |

Random effects:

| Groups | Name | Variance | Std.Dev. | Corr |
| --- | --- | --- | --- | --- |
| subject | (Intercept) | 0.2444 | 0.4944 |  |
|  | mapping_type_cc | 0.3292 | 0.5737 | 0.26 |

Number of obs: 2976, groups: subject, 62

Fixed effects:

|  | Estimate | Std. Error | z value | Pr(>\|z\|) |  | | |
| --- | --- | --- | --- | --- | --- | --- | --- |
| (Intercept) | 0.30909 | 0.075 | 4.122 | 3.76E-05 | | *** |  |
| mapping_type_cc | -0.09454 | 0.1097 | -0.862 | 0.389 |  | | |

---

Signif. codes: 0 ‘***’ 0.001 ‘**’ 0.01 ‘*’ 0.05 ‘.’ 0.1 ‘ ’ 1

Correlation of Fixed Effects:

(Intr)

mppng_typ_c 0.286

**Block 2:**

Generalized linear mixed model fit by maximum likelihood (Laplace Approximation) ['glmerMod']

Family: binomial ( logit )

Formula: accuracy ~ 1 + mapping_type_cc + (1 + mapping_type_cc | subject) + (1 | word)

+ (1 + mapping_type_cc | target)

Data: get(lp_var)

| AIC | BIC | logLik | deviance | df.resid |
| --- | --- | --- | --- | --- |
| 2132 | 2185.9 | -1057 | 2114 | 2967 |

Scaled residuals:

| Min | 1Q | Median | 3Q | Max |
| --- | --- | --- | --- | --- |
| -7.213 | 0.1628 | 0.2497 | 0.3836 | 1.5435 |

Random effects:

| Groups | Name | Variance | Std.Dev. | Corr |
| --- | --- | --- | --- | --- |
| subject | (Intercept) | 1.71871 | 1.311 |  |
|  | mapping_type_cc | 0.39268 | 0.6266 | 0.49 |
| target | (Intercept) | 0.08459 | 0.2908 |  |
|  | mapping_type_cc | 0.37368 | 0.6113 | 1.00 |
| word | (Intercept) | 0.11548 | 0.3398 |  |

Number of obs: 2976, groups: subject, 62; target, 28; word, 12

Fixed effects:

|  | Estimate | Std. Error | z value | Pr(>\|z\|) |  |
| --- | --- | --- | --- | --- | --- |
| (Intercept) | 2.3749 | 0.2202 | 10.788 | <2e-16 | *** |
| mapping_type_cc | 0.1193 | 0.2213 | 0.539 | 0.59 |  |

---

Signif. codes: 0 ‘***’ 0.001 ‘**’ 0.01 ‘*’ 0.05 ‘.’ 0.1 ‘ ’ 1

Correlation of Fixed Effects:

(Intr)

mppng_typ_c 0.399

**Block 3:**

Generalized linear mixed model fit by maximum likelihood (Laplace Approximation) ['glmerMod']

Family: binomial ( logit )

Formula: accuracy ~ 1 + mapping_type_cc + (1 | subject) + (1 | word)

Data: get(lp_var)

| AIC | BIC | logLik | deviance | df.resid |
| --- | --- | --- | --- | --- |
| 1318.5 | 1342.5 | -655.3 | 1310.5 | 2972 |

Scaled residuals:

| Min | 1Q | Median | 3Q | Max |
| --- | --- | --- | --- | --- |
| -8.1788 | 0.0747 | 0.1231 | 0.2374 | 1.8843 |

Random effects:

| Groups | Name | Variance | Std.Dev. |
| --- | --- | --- | --- |
| subject | (Intercept) | 4.11505 | 2.0286 |
| word | (Intercept) | 0.07457 | 0.2731 |

Number of obs: 2976, groups: subject, 62; word, 12

Fixed effects:

|  | Estimate | Std. Error | z value | Pr(>\|z\|) |  |
| --- | --- | --- | --- | --- | --- |
| mapping_type_cc | -0.6685 | 0.1576 | -4.241 | 2.22E-05 | *** |

---

Signif. codes: 0 ‘***’ 0.001 ‘**’ 0.01 ‘*’ 0.05 ‘.’ 0.1 ‘ ’ 1

Correlation of Fixed Effects:

(Intr)

mppng_typ_c 0.003

**Block 4:**

Generalized linear mixed model fit by maximum likelihood (Laplace Approximation) ['glmerMod']

Family: binomial ( logit )

Formula: accuracy ~ 1 + mapping_type_cc + (1 | subject)

Data: get(lp_var)

| AIC | BIC | logLik | deviance | df.resid |
| --- | --- | --- | --- | --- |
| 1048.3 | 1066.3 | -521.1 | 1042.3 | 2973 |

Scaled residuals:

| Min | 1Q | Median | 3Q | Max |
| --- | --- | --- | --- | --- |
| -7.4678 | 0.0673 | 0.0763 | 0.1519 | 1.6039 |

Random effects:

| Groups | Name | Variance | Std.Dev. |
| --- | --- | --- | --- |
| subject | (Intercept) | 4.404 | 2.098 |

Number of obs: 2976, groups: subject, 62

Fixed effects:

|  | Estimate | Std. Error | z value | Pr(>\|z\|) |  |
| --- | --- | --- | --- | --- | --- |
| (Intercept) | 4.2279 | 0.3723 | 11.357 | <2e-16 | *** |
| mapping_type_cc | -0.2519 | 0.1798 | -1.401 | 0.161 |  |

---

Signif. codes: 0 ‘***’ 0.001 ‘**’ 0.01 ‘*’ 0.05 ‘.’ 0.1 ‘ ’ 1

Correlation of Fixed Effects:

(Intr)

mppng_typ_c 0.051

**Block 5:**

Generalized linear mixed model fit by maximum likelihood (Laplace Approximation) ['glmerMod']

Family: binomial ( logit )

Formula: accuracy ~ 1 + mapping_type_cc + (1 | subject) + (1 | word)

Data: get(lp_var)

| AIC | BIC | logLik | deviance | df.resid |
| --- | --- | --- | --- | --- |
| 809.9 | 833.9 | -401 | 801.9 | 2972 |

Scaled residuals:

| Min | 1Q | Median | 3Q | Max |
| --- | --- | --- | --- | --- |
| -10.048 | 0.0448 | 0.0596 | 0.154 | 1.6478 |

Random effects:

| Groups | Name | Variance | Std.Dev. |
| --- | --- | --- | --- |
| subject | (Intercept) | 6.3809 | 2.5261 |
| word | (Intercept) | 0.1403 | 0.3746 |

Number of obs: 2976, groups: subject, 62; word, 12

Fixed effects:

|  | Estimate | Std. Error | z value | Pr(>\|z\|) |  |
| --- | --- | --- | --- | --- | --- |
| (Intercept) | 5.136 | 0.549 | 9.355 | < 2e-16 | *** |
| mapping_type_cc | -0.7187 | 0.2132 | -3.371 | 0.00075 | *** |

---

Signif. codes: 0 ‘***’ 0.001 ‘**’ 0.01 ‘*’ 0.05 ‘.’ 0.1 ‘ ’ 1

Correlation of Fixed Effects:

(Intr)

mppng_typ_c -0.027

**H2b:**

Formula: accuracy ~ mapping_type_cc + (1 | subject)

Data: phase2_firstblock

| AIC | BIC | logLik | deviance | df.resid |
| --- | --- | --- | --- | --- |
| 3990.6 | 4008.6 | -1992.3 | 3984.6 | 2973 |

Scaled residuals:

| Min | 1Q | Median | 3Q | Max |
| --- | --- | --- | --- | --- |
| -1.7936 | -1.0627 | 0.6213 | 0.8588 | 1.4355 |

Random effects:

| Groups | Name | Variance | Std.Dev. |
| --- | --- | --- | --- |
| subject | (Intercept) | 0.2211 | 0.4702 |

Number of obs: 2976, groups: subject, 62

Fixed effects:

|  | Estimate | Std. Error | z value | Pr(>\|z\|) |  |
| --- | --- | --- | --- | --- | --- |
| (Intercept) | 0.30311 | 0.07209 | 4.205 | 2.61E-05 | *** |
| mapping_type_cc | -0.10175 | 0.08048 | -1.264 | 0.206 |  |

---

Signif. codes: 0 ‘***’ 0.001 ‘**’ 0.01 ‘*’ 0.05 ‘.’ 0.1 ‘ ’ 1

Correlation of Fixed Effects:

(Intr)

mppng_typ_c 0.182

**H2c:**

Formula: accuracy ~ 1 + mapping_type_cc + acc_1 + (1 + mapping_type_cc | subject)

Data: phase2_covariate

| AIC | BIC | logLik | deviance | df.resid |
| --- | --- | --- | --- | --- |
| 3979.4 | 4015.4 | -1983.7 | 3967.4 | 2970 |

Scaled residuals:

| Min | 1Q | Median | 3Q | Max |
| --- | --- | --- | --- | --- |
| -1.8984 | -1.0268 | 0.6095 | 0.8196 | 1.5955 |

Random effects:

| Groups | Name | Variance | Std.Dev. | Corr |
| --- | --- | --- | --- | --- |
| subject | (Intercept) | 0.2342 | 0.4839 |  |
|  | mapping_type_cc | 0.3316 | 0.5758 | 0.26 |

Number of obs: 2976, groups: subject, 62

Fixed effects:

|  | Estimate | Std. Error | z value | Pr(>\|z\|) |  |
| --- | --- | --- | --- | --- | --- |
| (Intercept) | 0.05258 | 0.14393 | 0.365 | 0.715 |  |
| mapping_type_cc | -0.09753 | 0.1099 | -0.887 | 0.375 |  |
| acc_1 | 0.28299 | 0.13636 | 2.075 | 0.038 | * |

---

Signif. codes: 0 ‘***’ 0.001 ‘**’ 0.01 ‘*’ 0.05 ‘.’ 0.1 ‘ ’ 1

Correlation of Fixed Effects:

|  | (Intr) | mppn__ |
| --- | --- | --- |
| mppng_typ_c | 0.159 |  |
| acc_1 | -0.858 | -0.014 |

**E1:**

Linear mixed model fit by REML. t-tests use Satterthwaite's method ['lmerModLmerTest']

Formula: log_rt ~ 1 + mapping_type_cc + block_s + mapping_type_cc:block_s +

(1 + mapping_type_cc | subject) + (1 + block_s | word) + (1 | target)

Data: phase1_correct

REML criterion at convergence: 14398

Scaled residuals:

Min 1Q Median 3Q Max

-3.2636 -0.6946 -0.1318 0.5889 4.8474

Random effects:

| Groups | Name | Variance | Std.Dev. | Corr |
| --- | --- | --- | --- | --- |
| subject | (Intercept) | 0.046577 | 0.21582 |  |
|  | mapping_type_cc | 0.032371 | 0.17992 | 0.45 |
| target | (Intercept) | 0.001813 | 0.04258 |  |
| word | (Intercept) | 0.005086 | 0.07132 |  |
|  | block_s | 0.000254 | 0.01592 | 0.13 |
| Residual |  | 0.204711 | 0.45245 |  |

Number of obs: 11146, groups: subject, 62; target, 28; word, 12

Fixed effects:

|  | Estimate | Std. Error | df | t value | Pr(>\|t\|) |  |
| --- | --- | --- | --- | --- | --- | --- |
| (Intercept) | 7.63E+00 | 3.56E-02 | 5.69E+01 | 214.7 | < 2e-16 | *** |
| mapping_type_cc | 3.60E-01 | 2.50E-02 | 6.12E+01 | 14.42 | < 2e-16 | *** |
| block_s | -1.36E-01 | 5.81E-03 | 1.25E+01 | -23.35 | 1.07E-11 | *** |
| mapping_type_cc:block_s | 5.16E-02 | 7.12E-03 | 1.09E+04 | 7.24 | 4.78E-13 | *** |

---

Signif. codes: 0 ‘***’ 0.001 ‘**’ 0.01 ‘*’ 0.05 ‘.’ 0.1 ‘ ’ 1

Correlation of Fixed Effects:

|  | (Intr) | mppn__ | blck_s |
| --- | --- | --- | --- |
| mppng_typ_c | 0.339 |  |  |
| block_s | 0.043 | -0.024 |  |
| mppng_ty_:_ | -0.014 | -0.088 | 0.253 |

**Post – hoc (Mapping type * Block)**

**Block 1:**

Linear mixed model fit by REML. t-tests use Satterthwaite's method ['lmerModLmerTest']

Formula: log_rt ~ 1 + mapping_type_cc + (1 | subject) + (1 | word)

Data: LP1_RT_1

REML criterion at convergence: 1887.8

Scaled residuals:

| Min | 1Q | Median | 3Q | Max |
| --- | --- | --- | --- | --- |
| -2.93065 | -0.64508 | -0.04556 | 0.69432 | 2.77615 |

Random effects:

| Groups | Name | Variance | Std.Dev. |
| --- | --- | --- | --- |
| subject | (Intercept) | 0.067543 | 0.25989 |
| word | (Intercept) | 0.003388 | 0.05821 |
| Residual |  | 0.212779 | 0.46128 |

Number of obs: 1350, groups: subject, 62; word, 12

Fixed effects:

|  | Estimate | Std. Error | df | t value | Pr(>\|t\|) |  |
| --- | --- | --- | --- | --- | --- | --- |
| (Intercept) | 8.00E+00 | 3.97E-02 | 5.67E+01 | 201.758 | < 2e-16 | *** |
| mapping_type_cc | 1.68E-01 | 2.83E-02 | 1.30E+03 | 5.939 | 3.68E-09 | *** |

---

Signif. codes: 0 ‘***’ 0.001 ‘**’ 0.01 ‘*’ 0.05 ‘.’ 0.1 ‘ ’ 1

Correlation of Fixed Effects:

(Intr)

mppng_typ_c 0.144

**Block 2:**

Linear mixed model fit by REML. t-tests use Satterthwaite's method ['lmerModLmerTest']

Formula: log_rt ~ 1 + mapping_type_cc + (1 + mapping_type_cc | subject) +

(1 | word) + (1 | target)

Data: LP1_RT_2

REML criterion at convergence: 2886.9

Scaled residuals:

| Min | 1Q | Median | 3Q | Max |
| --- | --- | --- | --- | --- |
| -2.7026 | -0.6953 | -0.1217 | 0.6154 | 3.3398 |

Random effects:

| Groups | Name | Variance | Std.Dev. | Corr |
| --- | --- | --- | --- | --- |
| subject | (Intercept) | 0.060899 | 0.24678 |  |
|  | mapping_type_cc | 0.050686 | 0.22513 | 0.34 |
| target | (Intercept) | 0.003534 | 0.05944 |  |
| word | (Intercept) | 0.010776 | 0.10381 |  |
| Residual |  | 0.208684 | 0.45682 |  |

Number of obs: 2074, groups: subject, 62; target, 28; word, 12

Fixed effects:

|  | Estimate | Std. Error | df | t value | Pr(>\|t\|) |  |
| --- | --- | --- | --- | --- | --- | --- |
| (Intercept) | 7.741 | 0.04629 | 39.69722 | 167.24 | < 2e-16 | *** |
| mapping_type_cc | 0.33362 | 0.03703 | 61.72459 | 9.01 | 7.42E-13 | *** |

---

Signif. codes: 0 ‘***’ 0.001 ‘**’ 0.01 ‘*’ 0.05 ‘.’ 0.1 ‘ ’ 1

Correlation of Fixed Effects:

(Intr)

mppng_typ_c 0.250

**Block 3:**

Linear mixed model fit by REML. t-tests use Satterthwaite's method ['lmerModLmerTest']

Formula: log_rt ~ 1 + mapping_type_cc + (1 + mapping_type_cc | subject) +

(1 | word) + (1 | target)

Data: LP1_RT_3

REML criterion at convergence: 3155.1

Scaled residuals:

| Min | 1Q | Median | 3Q | Max |
| --- | --- | --- | --- | --- |
| -2.4234 | -0.6781 | -0.1318 | 0.5674 | 4.3244 |

Random effects:

| Groups | Name | Variance | Std.Dev. | Corr |
| --- | --- | --- | --- | --- |
| subject | (Intercept) | 0.060639 | 0.24625 |  |
|  | mapping_type_cc | 0.030622 | 0.17499 | 0.22 |
| target | (Intercept) | 0.003384 | 0.05817 |  |
| word | (Intercept) | 0.006307 | 0.07942 |  |
| Residual |  | 0.193728 | 0.44015 |  |

Number of obs: 2420, groups: subject, 62; target, 28; word, 12

Fixed effects:

|  | Estimate | Std. Error | df | t value | Pr(>\|t\|) |  |
| --- | --- | --- | --- | --- | --- | --- |
| (Intercept) | 7.55625 | 0.04158 | 54.45329 | 181.7 | <2e-16 | *** |
| mapping_type_cc | 0.39856 | 0.03027 | 61.69276 | 13.17 | <2e-16 | *** |

---

Signif. codes: 0 ‘***’ 0.001 ‘**’ 0.01 ‘*’ 0.05 ‘.’ 0.1 ‘ ’ 1

Correlation of Fixed Effects:

(Intr)

mppng_typ_c 0.197

**Block 4:**

Linear mixed model fit by REML. t-tests use Satterthwaite's method ['lmerModLmerTest']

Formula: log_rt ~ 1 + mapping_type_cc + (1 + mapping_type_cc | subject) +

(1 | word) + (1 | target)

Data: LP1_RT_4

REML criterion at convergence: 3170.4

Scaled residuals:

| Min | 1Q | Median | 3Q | Max |
| --- | --- | --- | --- | --- |
| -3.3336 | -0.6617 | -0.16 | 0.5373 | 4.5573 |

Random effects:

| Groups | Name | Variance | Std.Dev. | Corr |
| --- | --- | --- | --- | --- |
| subject | (Intercept) | 0.053384 | 0.23105 |  |
|  | mapping_type_cc | 0.056783 | 0.23829 | 0.62 |
| target | (Intercept) | 0.001861 | 0.04314 |  |
| word | (Intercept) | 0.006491 | 0.08057 |  |
| Residual |  | 0.177038 | 0.42076 |  |

Number of obs: 2621, groups: subject, 62; target, 28; word, 12

Fixed effects:

|  | Estimate | Std. Error | df | t value | Pr(>\|t\|) |  |
| --- | --- | --- | --- | --- | --- | --- |
| (Intercept) | 7.48149 | 0.03941 | 48.359 | 189.84 | <2e-16 | *** |
| mapping_type_cc | 0.43205 | 0.03549 | 59.58041 | 12.17 | <2e-16 | *** |

---

Signif. codes: 0 ‘***’ 0.001 ‘**’ 0.01 ‘*’ 0.05 ‘.’ 0.1 ‘ ’ 1

Correlation of Fixed Effects:

(Intr)

mppng_typ_c 0.442

**Block 5:**

Linear mixed model fit by REML. t-tests use Satterthwaite's method ['lmerModLmerTest']

Formula: log_rt ~ 1 + mapping_type_cc + (1 + mapping_type_cc | subject) +

(1 | word)

Data: LP1_RT_5

REML criterion at convergence: 3006.6

Scaled residuals:

| Min | 1Q | Median | 3Q | Max |
| --- | --- | --- | --- | --- |
| -2.992 | -0.6391 | -0.1701 | 0.4854 | 5.2957 |

Random effects:

| Groups | Name | Variance | Std.Dev. | Corr |
| --- | --- | --- | --- | --- |
| subject | (Intercept) | 0.046079 | 0.21466 |  |
|  | mapping_type_cc | 0.052718 | 0.2296 | 0.45 |
| word | (Intercept) | 0.004187 | 0.06471 |  |
| Residual |  | 0.163049 | 0.40379 |  |

Number of obs: 2681, groups: subject, 62; word, 12

Fixed effects:

|  | Estimate | Std. Error | df | t value | Pr(>\|t\|) |  |
| --- | --- | --- | --- | --- | --- | --- |
| (Intercept) | 7.42101 | 0.03416 | 51.03709 | 217.24 | <2e-16 | *** |
| mapping_type_cc | 0.42306 | 0.03395 | 58.78464 | 12.46 | <2e-16 | *** |

---

Signif. codes: 0 ‘***’ 0.001 ‘**’ 0.01 ‘*’ 0.05 ‘.’ 0.1 ‘ ’ 1

Correlation of Fixed Effects:

(Intr)

mppng_typ_c 0.362

**E2a:**

Linear mixed model fit by REML. t-tests use Satterthwaite's method ['lmerModLmerTest']

Formula: log_rt ~ 1 + mapping_type_cc + block_s + mapping_type_cc:block_s +

(1 + block_s + mapping_type_cc | subject) + (1 + mapping_type_cc |word) +

(1 + mapping_type_cc | target)

Data: phase2_correct

REML criterion at convergence: 14499.8

Scaled residuals:

| Min | 1Q | Median | 3Q | Max |
| --- | --- | --- | --- | --- |
| -4.4549 | -0.6578 | -0.125 | 0.5468 | 4.6596 |

Random effects:

| Groups | Name | Variance | Std.Dev. | Corr |  |
| --- | --- | --- | --- | --- | --- |
| subject | (Intercept) | 0.027738 | 0.16655 |  |  |
|  | block_s | 0.004508 | 0.06714 | 0.06 |  |
|  | mapping_type_cc | 0.008504 | 0.09222 | 0.08 | 0.42 |
| target | (Intercept) | 0.001525 | 0.03906 |  |  |
|  | mapping_type_cc | 0.0034 | 0.05831 | 0.06 |  |
| word | (Intercept) | 0.002825 | 0.05315 |  |  |
|  | mapping_type_cc | 0.001754 | 0.04188 | -0.37 |  |
| Residual |  | 0.178742 | 0.42278 |  |  |

Number of obs: 12498, groups: subject, 62; target, 28; word, 12

Fixed effects:

|  | Estimate | Std. Error | | df | | t value | | Pr(>\|t\|) | |  | |
| --- | --- | --- | --- | --- | --- | --- | --- | --- | --- | --- | --- |
| (Intercept) | 7.32E+00 | 2.75E-02 | | 5.83E+01 | | 266.113 | | <2e-16 | | *** | |
| mapping_type_cc | 2.48E-02 | 2.19E-02 | | 2.99E+01 | | 1.135 | | 0.265 | |  | |
| block_s | -1.47E-01 | 9.05E-03 | 5.94E+01 | | -16.203 | | <2e-16 | | *** | |  |
| mapping_type_cc:block_s | -8.88E-03 | 6.00E-03 | | 1.23E+04 | | -1.48 | | 0.139 | |  | |

---

Signif. codes: 0 ‘***’ 0.001 ‘**’ 0.01 ‘*’ 0.05 ‘.’ 0.1 ‘ ’ 1

Correlation of Fixed Effects:

|  | (Intr) | mppn__ | blck_s |
| --- | --- | --- | --- |
| mppng_typ_c | -0.053 |  |  |
| block_s | 0.035 | 0.207 |  |
| mppng_ty_:_ | -0.008 | -0.058 | 0.115 |

**E2b:**

Linear mixed model fit by REML. t-tests use Satterthwaite's method ['lmerModLmerTest']

Formula: log_rt ~ 1 + mapping_type_cc + (1 | subject)

Data: phase2_firstblock_correct

REML criterion at convergence: 2487.4

Scaled residuals:

| Min | 1Q | Median | 3Q | Max |
| --- | --- | --- | --- | --- |
| -3.11705 | -0.70893 | -0.04106 | 0.67685 | 2.72474 |

Random effects:

| Groups | Name | Variance | Std.Dev. |
| --- | --- | --- | --- |
| subject | (Intercept) | 0.0466 | 0.2159 |
| Residual |  | 0.2424 | 0.4924 |

Number of obs: 1666, groups: subject, 62

Fixed effects:

|  | Estimate | Std. Error | df | t value | Pr(>\|t\|) |  |
| --- | --- | --- | --- | --- | --- | --- |
| (Intercept) | 7.75E+00 | 3.04E-02 | 6.08E+01 | 254.654 | <2e-16 | *** |
| mapping_type_cc | 1.81E-02 | 2.60E-02 | 1.62E+03 | 0.694 | 0.488 |  |

---

Signif. codes: 0 ‘***’ 0.001 ‘**’ 0.01 ‘*’ 0.05 ‘.’ 0.1 ‘ ’ 1

Correlation of Fixed Effects:

(Intr)

mppng_typ_c 0.149

**E2c:**

Linear mixed model fit by REML. t-tests use Satterthwaite's method ['lmerModLmerTest']

Formula: log_rt ~ 1 + mapping_type_cc + acc_1 + (1 | subject)

Data: phase2_covariateRT

REML criterion at convergence: 2491.2

Scaled residuals:

| Min | 1Q | Median | 3Q | Max |
| --- | --- | --- | --- | --- |
| -3.11711 | -0.70999 | -0.03581 | 0.68496 | 2.71607 |

Random effects:

| Groups | Name | Variance | Std.Dev. |
| --- | --- | --- | --- |
| subject | (Intercept) | 0.04622 | 0.215 |
| Residual |  | 0.24258 | 0.4925 |

Number of obs: 1666, groups: subject, 62

Fixed effects:

|  | Estimate | Std. Error | df | t value | Pr(>\|t\|) |  |
| --- | --- | --- | --- | --- | --- | --- |
| (Intercept) | 7.72E+00 | 5.27E-02 | 4.46E+02 | 146.337 | <2e-16 | *** |
| mapping_type_cc | 1.73E-02 | 2.61E-02 | 1.62E+03 | 0.665 | 0.506 |  |
| acc_1 | 3.28E-02 | 4.69E-02 | 1.66E+03 | 0.7 | 0.484 |  |

---

Signif. codes: 0 ‘***’ 0.001 ‘**’ 0.01 ‘*’ 0.05 ‘.’ 0.1 ‘ ’ 1

Correlation of Fixed Effects:

|  | (Intr) | mppn__ |
| --- | --- | --- |
| mppng_typ_c | 0.12 |  |
| acc_1 | -0.818 | -0.042 |

**Experiment 1**

**MEANS**

*Phase 1 Accuracy per mapping type*

| Mapping type | Mean accuracy | SD accuracy |
| --- | --- | --- |
| 1:1 | 0.80 | 0.40 |
| 1:2 | 0.67 | 0.47 |

*Phase 1 Accuracy per block*

| Block | Mean accuracy | SD accuracy |
| --- | --- | --- |
| 1 | 0.47 | 0.50 |
| 2 | 0.71 | 0.46 |
| 3 | 0.82 | 0.38 |
| 4 | 0.89 | 0.32 |
| 5 | 0.91 | 0.29 |

*Phase 1 Accuracy per mapping type and block*

| Mapping | 1:1 | | 1:2 | |
| --- | --- | --- | --- | --- |
| Block | Mean accuracy | SD accuracy | Mean accuracy | SD accuracy |
| 1 | 0.49 | 0.50 | 0.41 | 0.49 |
| 2 | 0.77 | 0.42 | 0.60 | 0.49 |
| 3 | 0.88 | 0.33 | 0.71 | 0.46 |
| 4 | 0.93 | 0.25 | 0.80 | 0.40 |
| 5 | 0.95 | 0.22 | 0.82 | 0.39 |

*Phase 2 Accuracy per mapping type*

| Mapping type | Mean accuracy | SD accuracy |
| --- | --- | --- |
| 1:1 | 0.85 | 0.36 |
| 1:2 | 0.83 | 0.38 |

*Phase 2 Accuracy per block*

| Block | Mean accuracy | SD accuracy |
| --- | --- | --- |
| 1 | 0.58 | 0.49 |
| 2 | 0.86 | 0.35 |
| 3 | 0.91 | 0.29 |
| 4 | 0.93 | 0.25 |
| 5 | 0.95 | 0.22 |

*Phase 2 Accuracy per mapping type and block*

| Mapping | 1:1 | | 1:2 | |
| --- | --- | --- | --- | --- |
| Block | Mean accuracy | SD accuracy | Mean accuracy | SD accuracy |
| 1 | 0.58 | 0.49 | 0.56 | 0.50 |
| 2 | 0.86 | 0.35 | 0.85 | 0.36 |
| 3 | 0.92 | 0.27 | 0.88 | 0.32 |
| 4 | 0.94 | 0.24 | 0.93 | 0.26 |
| 5 | 0.96 | 0.21 | 0.93 | 0.25 |

*Phase 1 RTs per mapping type*

| Mapping type | Mean accuracy (ms) | SD accuracy (ms) |
| --- | --- | --- |
| 1:1 | 1934 | 1345 |
| 1:2 | 2779 | 1680 |

*Phase 1 RTs per block*

| Block | Mean accuracy (ms) | SD accuracy (ms) |
| --- | --- | --- |
| 1 | 3303 | 1829 |
| 2 | 2481 | 1594 |
| 3 | 2041 | 1371 |
| 4 | 1904 | 1304 |
| 5 | 1774 | 1180 |

*Phase 1 RTs per mapping type and block*

| Mapping | 1:1 | | 1:2 | |
| --- | --- | --- | --- | --- |
| Block | Mean accuracy (ms) | SD accuracy (ms) | Mean accuracy (ms) | SD accuracy (ms) |
| 1 | 3179 | 1827 | 3605 | 1801 |
| 2 | 2238 | 1461 | 3114 | 1744 |
| 3 | 1784 | 1156 | 2689 | 1632 |
| 4 | 1611 | 1001 | 2597 | 1636 |
| 5 | 1512 | 917 | 2386 | 1464 |

*Phase 2 RTs per mapping type*

| Mapping type | Mean accuracy (ms) | SD accuracy (ms) |
| --- | --- | --- |
| 1:1 | 1676 | 1106 |
| 1:2 | 1715 | 1138 |

*Phase 2 RTs per block*

| Block | Mean accuracy (ms) | SD accuracy (ms) |
| --- | --- | --- |
| 1 | 2661 | 1573 |
| 2 | 1914 | 1237 |
| 3 | 1531 | 906 |
| 4 | 1410 | 748 |
| 5 | 1336 | 725 |

*Phase 2 RTs per mapping type and block*

| Mapping | 1:1 | | 1:2 | |
| --- | --- | --- | --- | --- |
| Block | Mean accuracy (ms) | SD accuracy (ms) | Mean accuracy (ms) | SD accuracy (ms) |
| 1 | 2626 | 1555 | 2733 | 1607 |
| 2 | 1898 | 1231 | 1948 | 1249 |
| 3 | 1505 | 865 | 1587 | 983 |
| 4 | 1411 | 758 | 1408 | 728 |
| 5 | 1339 | 750 | 1330 | 670 |

**GRAPHS**

**RTs – LP1**

**
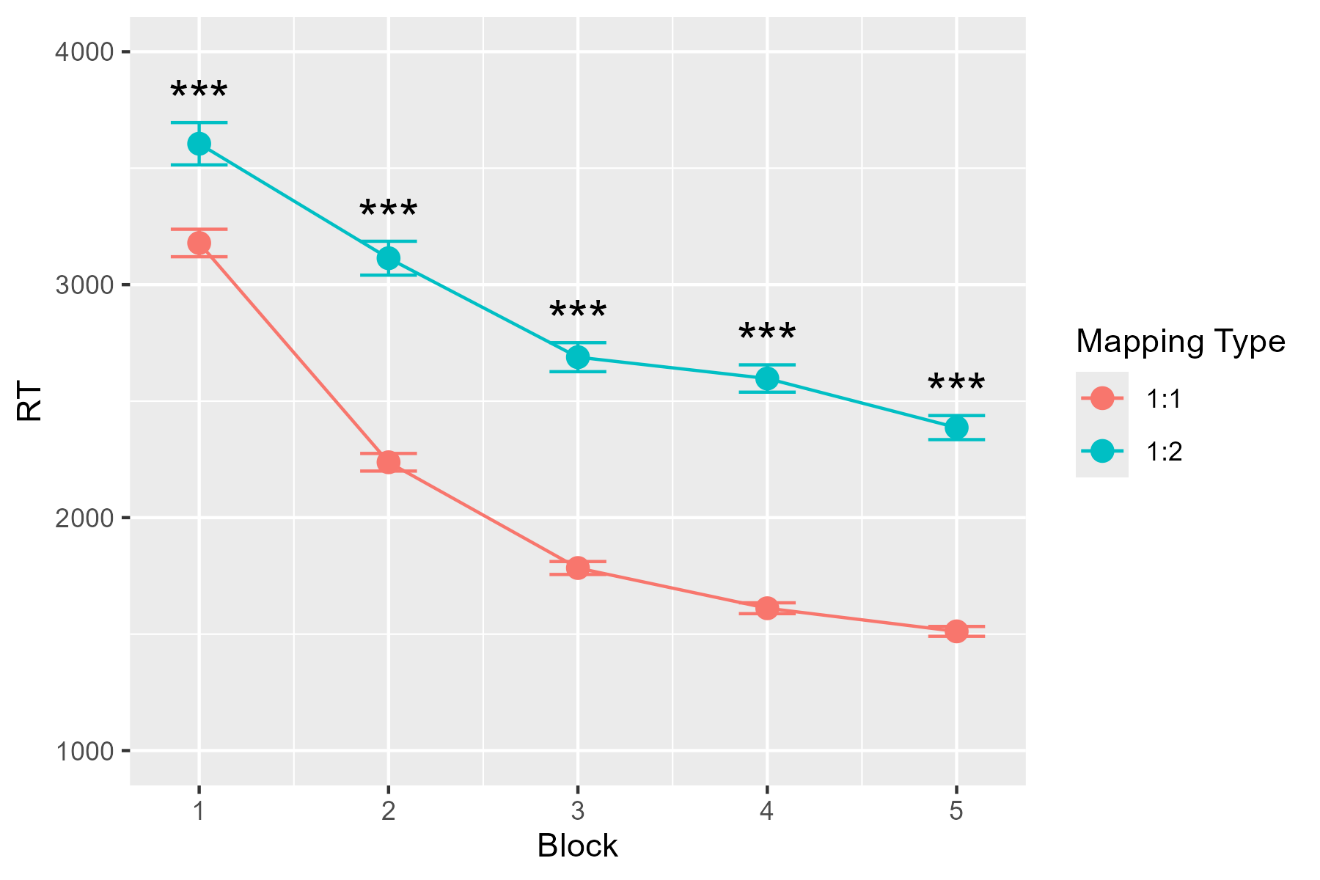
**

**RTs – LP2**

**
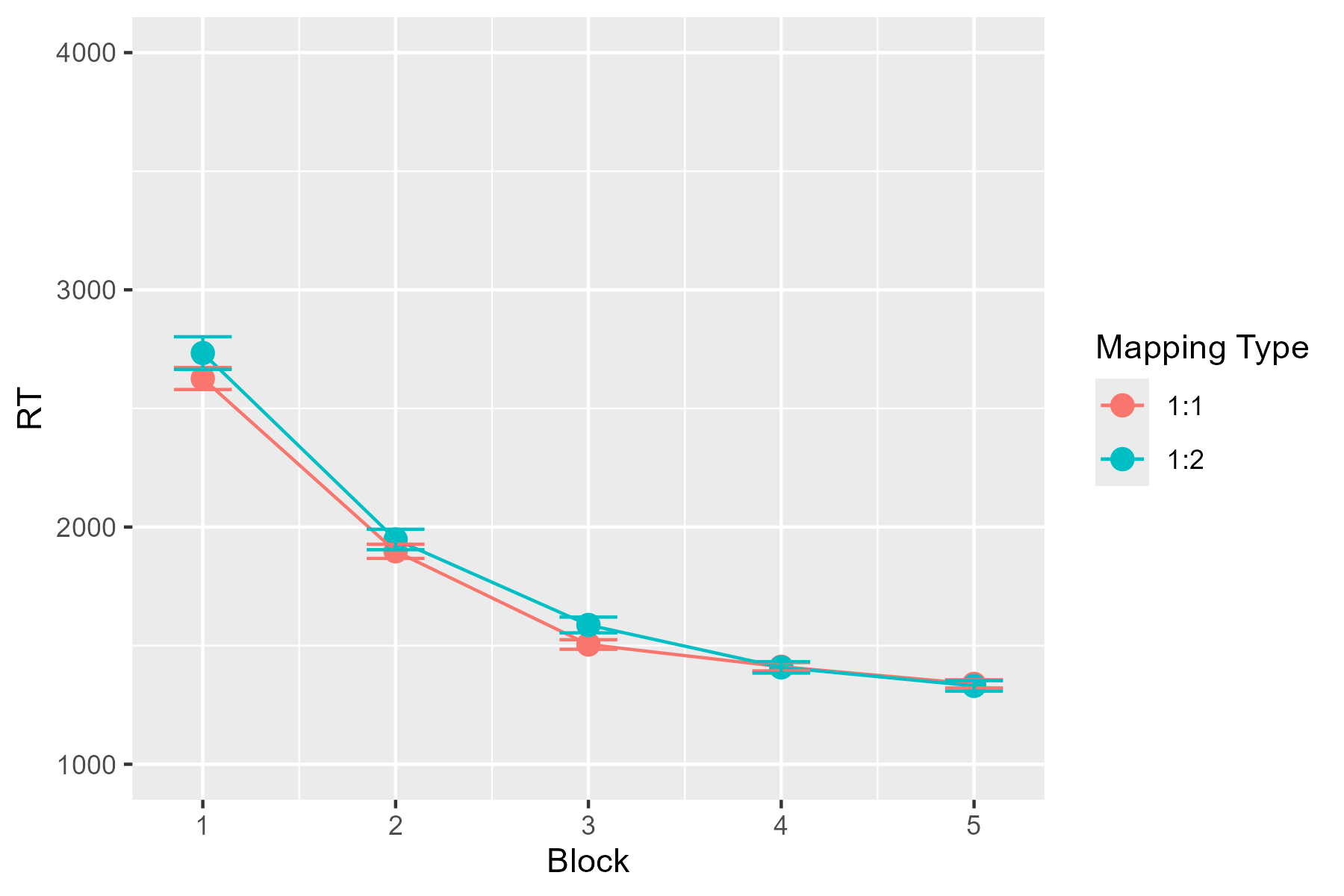
**

**Experiment 2**

**RESULTS**

**H1**

Full results:

Formula: accuracy ~ 1 + mapping_type_cc + block_s + mapping_type_cc:block_s +

(1 + block_s + mapping_type_cc | subject) + (1 + mapping_type_cc | target) + (1 | word)

Data: phase1

| AIC | BIC | logLik | deviance | df.resid |
| --- | --- | --- | --- | --- |
| 12518.3 | 12624.3 | -6245.1 | 12490.3 | 14386 |

Scaled residuals:

| Min | 1Q | Median | 3Q | Max |
| --- | --- | --- | --- | --- |
| -47.751 | 0.026 | 0.239 | 0.541 | 2.218 |

Random effects:

| Groups | Name | Variance | Std.Dev. | Corr |  |
| --- | --- | --- | --- | --- | --- |
| subject | (Intercept) | 1.07159 | 1.0352 |  |  |
|  | block_s | 0.19108 | 0.4371 | 0.95 |  |
|  | mapping_type_cc | 0.32036 | 0.566 | -0.58 | -0.53 |
| target | (Intercept) | 0.08375 | 0.2894 |  |  |
|  | mapping_type_cc | 0.2425 | 0.4924 | -0.34 |  |
| word | (Intercept) | 0.02966 | 0.1722 |  |  |

Number of obs: 14400, groups: subject, 60; target, 27; word, 12

Fixed effects:

|  | Estimate | Std. Error | z value | Pr(>\|z\|) |  |
| --- | --- | --- | --- | --- | --- |
| (Intercept) | 1.65899 | 0.15914 | 10.425 | < 2e-16 | *** |
| mapping_type_cc | -1.12275 | 0.14446 | -7.772 | 7.72E-15 | *** |
| block_s | 0.86235 | 0.06107 | 14.121 | < 2e-16 | *** |
| mapping_type_cc:block_s | -0.23946 | 0.03778 | -6.338 | 2.32E-10 | *** |

---

Signif. codes: 0 ‘***’ 0.001 ‘**’ 0.01 ‘*’ 0.05 ‘.’ 0.1 ‘ ’ 1

Correlation of Fixed Effects:

|  | (Intr) | mppn__ | blck_s |
| --- | --- | --- | --- |
| mppng_typ_c | -0.339 |  |  |
| block_s | 0.795 | -0.273 |  |
| mppng_ty_:_ | -0.029 | 0.199 | 0.005 |

**Post – hoc (Mapping type * Block)**

**Block 1:**

Generalized linear mixed model fit by maximum likelihood (Laplace Approximation) ['glmerMod']

Family: binomial ( logit )

Formula: accuracy ~ 1 + mapping_type_cc + (1 + mapping_type_cc | target) + (1 | subject) +

(1 | word)

Data: get(lp_var)

| AIC | BIC | logLik | deviance | df.resid |
| --- | --- | --- | --- | --- |
| 3906 | 3947.8 | -1946 | 3892 | 2873 |

Scaled residuals:

| Min | 1Q | Median | 3Q | Max |
| --- | --- | --- | --- | --- |
| -1.8502 | -0.9176 | -0.6723 | 0.9537 | 1.5989 |

Random effects:

| Groups | Name | Variance | Std.Dev. | Corr |
| --- | --- | --- | --- | --- |
| subject | (Intercept) | 0.07074 | 0.266 |  |
| target | (Intercept) | 0.0702 | 0.2649 |  |
|  | mapping_type_cc | 0.10662 | 0.3265 | -0.93 |
| word | (Intercept) | 0.02814 | 0.1677 |  |

Number of obs: 2880, groups: subject, 60; target, 27; word, 12

Fixed effects:

|  | Estimate | Std. Error | z value | Pr(>\|z\|) |  |
| --- | --- | --- | --- | --- | --- |
| (Intercept) | -0.1819 | 0.09161 | -1.985 | 0.0471 | * |
| mapping_type_cc | -0.66161 | 0.12261 | -5.396 | 6.81E-08 | *** |

---

Signif. codes: 0 ‘***’ 0.001 ‘**’ 0.01 ‘*’ 0.05 ‘.’ 0.1 ‘ ’ 1

Correlation of Fixed Effects:

(Intr)

mppng_typ_c -0.158

**Block 2:**

Generalized linear mixed model fit by maximum likelihood (Laplace Approximation) ['glmerMod']

Family: binomial ( logit )

Formula: accuracy ~ 1 + mapping_type_cc + (1 + mapping_type_cc | subject) + (1 | target) +

(1 + mapping_type_cc | word)

Data: get(lp_var)

| AIC | BIC | logLik | deviance | df.resid |
| --- | --- | --- | --- | --- |
| 3076.9 | 3130.6 | -1529.4 | 3058.9 | 2871 |

Scaled residuals:

| Min | 1Q | Median | 3Q | Max |
| --- | --- | --- | --- | --- |
| -3.8696 | -0.7947 | 0.3647 | 0.6071 | 2.0879 |

Random effects:

| Groups | Name | Variance | Std.Dev. | Corr |
| --- | --- | --- | --- | --- |
| subject | (Intercept) | 0.79998 | 0.8944 |  |
|  | mapping_type_cc | 0.41433 | 0.6437 | -0.81 |
| target | (Intercept) | 0.18116 | 0.4256 |  |
| word | (Intercept) | 0.02207 | 0.1485 |  |
|  | mapping_type_cc | 0.23082 | 0.4804 | 0.74 |

Number of obs: 2880, groups: subject, 60; target, 27; word, 12

Fixed effects:

|  | Estimate | Std. Error | z value | Pr(>\|z\|) |  |
| --- | --- | --- | --- | --- | --- |
| (Intercept) | 0.9846 | 0.1608 | 6.123 | 9.18E-10 | *** |
| mapping_type_cc | -1.0224 | 0.2061 | -4.96 | 7.04E-07 | *** |

---

Signif. codes: 0 ‘***’ 0.001 ‘**’ 0.01 ‘*’ 0.05 ‘.’ 0.1 ‘ ’ 1

Correlation of Fixed Effects:

(Intr)

mppng_typ_c -0.044

**Block 3:**

Generalized linear mixed model fit by maximum likelihood (Laplace Approximation) ['glmerMod']

Family: binomial ( logit )

Formula: accuracy ~ 1 + mapping_type_cc + (1 + mapping_type_cc | subject) + (1 | word) +

(1 + mapping_type_cc | target)

Data: get(lp_var)

| AIC | BIC | logLik | deviance | df.resid |
| --- | --- | --- | --- | --- |
| 2419 | 2472.7 | -1200.5 | 2401 | 2871 |

Scaled residuals:

| Min | 1Q | Median | 3Q | Max |
| --- | --- | --- | --- | --- |
| -6.9936 | 0.1317 | 0.2856 | 0.4885 | 1.8995 |

Random effects:

| Groups | Name | Variance | Std.Dev. | Corr |
| --- | --- | --- | --- | --- |
| subject | (Intercept) | 1.13802 | 1.0668 |  |
|  | mapping_type_cc | 0.47262 | 0.6875 | -1 |
| target | (Intercept) | 0.09655 | 0.3107 |  |
|  | mapping_type_cc | 0.62103 | 0.7881 | 0.41 |
| word | (Intercept) | 0.09809 | 0.3132 |  |

Number of obs: 2880, groups: subject, 60; target, 27; word, 12

Fixed effects:

|  | Estimate | Std. Error | z value | Pr(>\|z\|) |  |
| --- | --- | --- | --- | --- | --- |
| (Intercept) | 1.8165 | 0.193 | 9.414 | < 2e-16 | *** |
| mapping_type_cc | -1.3927 | 0.2405 | -5.79 | 7.04E-09 | *** |

---

Signif. codes: 0 ‘***’ 0.001 ‘**’ 0.01 ‘*’ 0.05 ‘.’ 0.1 ‘ ’ 1

Correlation of Fixed Effects:

(Intr)

mppng_typ_c -0.216

**Block 4:**

Generalized linear mixed model fit by maximum likelihood (Laplace Approximation) ['glmerMod']

Family: binomial ( logit )

Formula: accuracy ~ 1 + mapping_type_cc + (1 + mapping_type_cc | subject) + (1 | word) +

(1 | target)

Data: get(lp_var)

| AIC | BIC | logLik | deviance | df.resid |
| --- | --- | --- | --- | --- |
| 1853.9 | 1895.7 | -920 | 1839.9 | 2873 |

Scaled residuals:

| Min | 1Q | Median | 3Q | Max |
| --- | --- | --- | --- | --- |
| -8.6487 | 0.1148 | 0.2121 | 0.3659 | 1.79 |

Random effects:

| Groups | Name | Variance | Std.Dev. | Corr |
| --- | --- | --- | --- | --- |
| subject | (Intercept) | 1.8467 | 1.3589 |  |
|  | mapping_type_cc | 0.1617 | 0.4021 | -1 |
| target | (Intercept) | 0.1841 | 0.4291 |  |
| word | (Intercept) | 0.135 | 0.3675 |  |

Number of obs: 2880, groups: subject, 60; target, 27; word, 12

Fixed effects:

|  | Estimate | Std. Error | z value | Pr(>\|z\|) |  |
| --- | --- | --- | --- | --- | --- |
| (Intercept) | 2.4794 | 0.2415 | 10.267 | < 2e-16 | *** |
| mapping_type_cc | -1.2316 | 0.2012 | -6.123 | 9.20E-10 | *** |

---

Signif. codes: 0 ‘***’ 0.001 ‘**’ 0.01 ‘*’ 0.05 ‘.’ 0.1 ‘ ’ 1

Correlation of Fixed Effects:

(Intr)

mppng_typ_c -0.239

**Block 5:**

Generalized linear mixed model fit by maximum likelihood (Laplace Approximation) ['glmerMod']

Family: binomial ( logit )

Formula: accuracy ~ 1 + mapping_type_cc + (1 | subject) + (1 + mapping_type_cc | target)

Data: get(lp_var)

| AIC | BIC | logLik | deviance | df.resid |
| --- | --- | --- | --- | --- |
| 1498.3 | 1534.1 | -743.1 | 1486.3 | 2874 |

Scaled residuals:

| Min | 1Q | Median | 3Q | Max |
| --- | --- | --- | --- | --- |
| -13.5193 | 0.0947 | 0.1686 | 0.282 | 2.6054 |

Random effects:

| Groups | Name | Variance | Std.Dev. | Corr |
| --- | --- | --- | --- | --- |
| subject | (Intercept) | 2.5292 | 1.5904 |  |
| target | (Intercept) | 0.1143 | 0.3381 |  |
|  | mapping_type_cc | 1.3194 | 1.1486 | -0.69 |

Number of obs: 2880, groups: subject, 60; target, 27

Fixed effects:

|  | Estimate | Std. Error | z value | Pr(>\|z\|) |  |
| --- | --- | --- | --- | --- | --- |
| (Intercept) | 3.0607 | 0.2521 | 12.143 | < 2e-16 | *** |
| mapping_type_cc | -1.3403 | 0.2865 | -4.677 | 2.90E-06 | *** |

---

Signif. codes: 0 ‘***’ 0.001 ‘**’ 0.01 ‘*’ 0.05 ‘.’ 0.1 ‘ ’ 1

Correlation of Fixed Effects:

(Intr)

mppng_typ_c -0.228

**H2a:**

Formula: accuracy ~ 1 + mapping_type_cc + block_s + mapping_type_cc:block_s +

(1 + block_s | subject) + (1 | target) + (1 | word)

Data: phase2

| AIC | BIC | logLik | deviance | df.resid |
| --- | --- | --- | --- | --- |
| 10099.5 | 10167.7 | -5040.8 | 10081.5 | 14391 |

Scaled residuals:

| Min | 1Q | Median | 3Q | Max |
| --- | --- | --- | --- | --- |
| -36.959 | 0.012 | 0.137 | 0.411 | 2.222 |

Random effects:

| Groups | Name | Variance | Std.Dev. | Corr |
| --- | --- | --- | --- | --- |
| subject | (Intercept) | 3.5226 | 1.8769 |  |
|  | block_s | 0.54883 | 0.7408 | 0.94 |
| target | (Intercept) | 0.08834 | 0.2972 |  |
| word | (Intercept) | 0.05256 | 0.2293 |  |

Number of obs: 14400, groups: subject, 60; target, 26; word, 12

Fixed effects:

|  | Estimate | Std. Error | z value | Pr(>\|z\|) |  |
| --- | --- | --- | --- | --- | --- |
| (Intercept) | 2.81597 | 0.26943 | 10.452 | <2e-16 | *** |
| mapping_type_cc | 0.15534 | 0.07898 | 1.967 | 0.0492 | * |
| block_s | 1.36513 | 0.10514 | 12.984 | <2e-16 | *** |
| mapping_type_cc:block_s | -0.05795 | 0.04321 | -1.341 | 0.1799 |  |

---

Signif. codes: 0 ‘***’ 0.001 ‘**’ 0.01 ‘*’ 0.05 ‘.’ 0.1 ‘ ’ 1

Correlation of Fixed Effects:

|  | (Intr) | mppn__ | blck_s |
| --- | --- | --- | --- |
| mppng_typ_c | 0.057 |  |  |
| block_s | 0.879 | 0.041 |  |
| mppng_ty_:_ | 0.026 | 0.477 | 0.074 |

**H2b:**

Formula: accuracy ~ mapping_type_cc + (1 | subject)

Data: phase2_firstblock

| AIC | BIC | logLik | deviance | df.resid |
| --- | --- | --- | --- | --- |
| 3880.4 | 3898.3 | -1937.2 | 3874.4 | 2877 |

Scaled residuals:

| Min | 1Q | Median | 3Q | Max |
| --- | --- | --- | --- | --- |
| -1.9788 | -0.9098 | -0.6244 | 0.9434 | 1.6015 |

Random effects:

| Groups | Name | Variance | Std.Dev. |
| --- | --- | --- | --- |
| subject | (Intercept) | 0.2707 | 0.5203 |

Number of obs: 2880, groups: subject, 60

Fixed effects:

|  | Estimate | Std. Error | z value | Pr(>\|z\|) |  |
| --- | --- | --- | --- | --- | --- |
| (Intercept) | 0.04353 | 0.07872 | 0.553 | 0.58 |  |
| mapping_type_cc | 0.40723 | 0.08204 | 4.964 | 6.90E-07 | *** |

---

Signif. codes: 0 ‘***’ 0.001 ‘**’ 0.01 ‘*’ 0.05 ‘.’ 0.1 ‘ ’ 1

Correlation of Fixed Effects:

(Intr)

mppng_typ_c 0.175

**H2c:**

Formula: accuracy ~ 1 + mapping_type_cc + acc_1 + (1 | subject) + (1 + mapping_type_cc | target) + (1 | word)

Data: phase2_covariate

AIC BIC logLik deviance df.resid

3852.2 3899.9 -1918.1 3836.2 2872

Scaled residuals:

| Min | 1Q | Median | 3Q | Max |
| --- | --- | --- | --- | --- |
| -2.1561 | -0.8872 | -0.5201 | 0.9129 | 2.0476 |

Random effects:

| Groups | Name | Variance | Std.Dev. | Corr |
| --- | --- | --- | --- | --- |
| subject | (Intercept) | 0.30788 | 0.5549 |  |
| target | (Intercept) | 0.10779 | 0.3283 |  |
|  | mapping_type_cc | 0.32623 | 0.5712 | 0.69 |
| word | (Intercept) | 0.03173 | 0.1781 |  |

Number of obs: 2880, groups: subject, 60; target, 26; word, 12

Fixed effects:

|  | Estimate | Std. Error | z value | Pr(>\|z\|) |  |
| --- | --- | --- | --- | --- | --- |
| (Intercept) | 0.1205 | 0.1797 | 0.671 | 0.5024 |  |
| mapping_type_cc | 0.4135 | 0.1694 | 2.442 | 0.0146 | * |
| acc_1 | -0.0901 | 0.1435 | -0.628 | 0.5301 |  |

---

Signif. codes: 0 ‘***’ 0.001 ‘**’ 0.01 ‘*’ 0.05 ‘.’ 0.1 ‘ ’ 1

Correlation of Fixed Effects:

|  | (Intr) | mppn__ |
| --- | --- | --- |
| mppng_typ_c | 0.328 |  |
| acc_1 | -0.717 | -0.008 |

**E1:**

Linear mixed model fit by REML. t-tests use Satterthwaite's method ['lmerModLmerTest']

Formula: log_rt ~ 1 + mapping_type_cc + block_s + mapping_type_cc:block_s +

(1 + mapping_type_cc + block_s | subject)

Data: phase1_correct

REML criterion at convergence: 12191.1

Scaled residuals:

| Min | 1Q | Median | 3Q | Max |
| --- | --- | --- | --- | --- |
| -3.4229 | -0.6859 | -0.1438 | 0.6005 | 4.0819 |

Random effects:

| Groups | Name | Variance | Std.Dev. | Corr |  |
| --- | --- | --- | --- | --- | --- |
| subject | (Intercept) | 0.048949 | 0.22124 |  |  |
|  | mapping_type_cc | 0.03063 | 0.17501 | 0.55 |  |
|  | block_s | 0.003476 | 0.05896 | -0.06 | -0.29 |
| Residual |  | 0.172902 | 0.41581 |  |  |

Number of obs: 10818, groups: subject, 60

Fixed effects:

|  | Estimate | Std. Error | df | t value | Pr(>\|t\|) |  |
| --- | --- | --- | --- | --- | --- | --- |
| (Intercept) | 7.55E+00 | 2.89E-02 | 5.86E+01 | 261.011 | < 2e-16 | *** |
| mapping_type_cc | 3.25E-01 | 2.44E-02 | 5.86E+01 | 13.332 | < 2e-16 | *** |
| block_s | -1.01E-01 | 8.31E-03 | 6.15E+01 | -12.143 | < 2e-16 | *** |
| mapping_type_cc:block_s | 4.86E-02 | 6.60E-03 | 1.07E+04 | 7.369 | 1.85E-13 | *** |

---

Signif. codes: 0 ‘***’ 0.001 ‘**’ 0.01 ‘*’ 0.05 ‘.’ 0.1 ‘ ’ 1

Correlation of Fixed Effects:

|  | (Intr) | mppn__ | blck_s |
| --- | --- | --- | --- |
| mppng_typ_c | 0.528 |  |  |
| block_s | -0.064 | -0.267 |  |
| mppng_ty_:_ | -0.019 | -0.082 | 0.169 |

**Post – hoc (Mapping type * Block)**

**Block 1:**

Linear mixed model fit by REML. t-tests use Satterthwaite's method ['lmerModLmerTest']

Formula: log_rt ~ 1 + mapping_type_cc + (1 + mapping_type_cc | subject) +

(1 | word) + (1 | target)

Data: LP1_RT_1

REML criterion at convergence: 1661.3

Scaled residuals:

| Min | 1Q | Median | 3Q | Max |
| --- | --- | --- | --- | --- |
| -2.90535 | -0.70564 | -0.05843 | 0.60649 | 2.89116 |

Random effects:

| Groups | Name | Variance | Std.Dev. | Corr |
| --- | --- | --- | --- | --- |
| subject | (Intercept) | 0.066212 | 0.25732 |  |
|  | mapping_type_cc | 0.015963 | 0.12635 | 0.61 |
| target | (Intercept) | 0.002629 | 0.05128 |  |
| word | (Intercept) | 0.00246 | 0.0496 |  |
| Residual |  | 0.173054 | 0.416 |  |

Number of obs: 1372, groups: subject, 60; target, 27; word, 12

Fixed effects:

|  | Estimate | Std. Error | df | t value | Pr(>\|t\|) |  |
| --- | --- | --- | --- | --- | --- | --- |
| (Intercept) | 7.79219 | 0.03994 | 57.93883 | 195.11 | < 2e-16 | *** |
| mapping_type_cc | 0.11689 | 0.03392 | 70.15778 | 3.446 | 0.000965 | *** |

---

Signif. codes: 0 ‘***’ 0.001 ‘**’ 0.01 ‘*’ 0.05 ‘.’ 0.1 ‘ ’ 1

Correlation of Fixed Effects:

(Intr)

mppng_typ_c 0.389

**Block 2:**

Linear mixed model fit by REML. t-tests use Satterthwaite's method ['lmerModLmerTest']

Formula: log_rt ~ 1 + mapping_type_cc + (1 + mapping_type_cc | subject) +

(1 | target) + (1 | word)

Data: LP1_RT_1

REML criterion at convergence: 2590

Scaled residuals:

| Min | 1Q | Median | 3Q | Max |
| --- | --- | --- | --- | --- |
| -2.6328 | -0.7052 | -0.1158 | 0.5894 | 3.3155 |

Random effects:

| Groups | Name | Variance | Std.Dev. | Corr |
| --- | --- | --- | --- | --- |
| subject | (Intercept) | 0.059851 | 0.24464 |  |
|  | mapping_type_cc | 0.023304 | 0.15266 | 0.16 |
| target | (Intercept) | 0.007092 | 0.08421 |  |
| word | (Intercept) | 0.002755 | 0.05249 |  |
| Residual |  | 0.185447 | 0.43064 |  |

Number of obs: 2044, groups: subject, 60; target, 27; word, 12

Fixed effects:

|  | Estimate | Std. Error | df | t value | Pr(>\|t\|) |  |
| --- | --- | --- | --- | --- | --- | --- |
| (Intercept) | 7.65421 | 0.0404 | 69.49695 | 189.446 | < 2e-16 | *** |
| mapping_type_cc | 0.29919 | 0.03293 | 82.52075 | 9.086 | 4.60E-14 | *** |

---

Signif. codes: 0 ‘***’ 0.001 ‘**’ 0.01 ‘*’ 0.05 ‘.’ 0.1 ‘ ’ 1

Correlation of Fixed Effects:

(Intr)

mppng_typ_c 0.173

**Block 3:**

Linear mixed model fit by REML. t-tests use Satterthwaite's method ['lmerModLmerTest']

Formula: log_rt ~ 1 + mapping_type_cc + (1 + mapping_type_cc | subject) +

(1 + mapping_type_cc | target) + (1 | word)

Data: LP_RT_3

REML criterion at convergence: 2638.7

Scaled residuals:

| Min | 1Q | Median | 3Q | Max |
| --- | --- | --- | --- | --- |
| -2.78 | -0.6715 | -0.1283 | 0.5361 | 4.473 |

Random effects:

| Groups | Name | Variance | Std.Dev. | Corr |
| --- | --- | --- | --- | --- |
| subject | (Intercept) | 0.051903 | 0.22782 |  |
|  | mapping_type_cc | 0.025912 | 0.16097 | 0.44 |
| target | (Intercept) | 0.010225 | 0.10112 |  |
|  | mapping_type_cc | 0.011343 | 0.1065 | -0.68 |
| word | (Intercept) | 0.004053 | 0.06366 |  |
| Residual |  | 0.16117 | 0.40146 |  |

Number of obs: 2334, groups: subject, 60; target, 27; word, 12

Fixed effects:

|  | Estimate | Std. Error | df | t value | Pr(>\|t\|) |  |
| --- | --- | --- | --- | --- | --- | --- |
| (Intercept) | 7.51971 | 0.04136 | 60.23108 | 181.807 | < 2e-16 | *** |
| mapping_type_cc | 0.38777 | 0.03898 | 37.50795 | 9.948 | 4.55E-12 | *** |

---

Signif. codes: 0 ‘***’ 0.001 ‘**’ 0.01 ‘*’ 0.05 ‘.’ 0.1 ‘ ’ 1

Correlation of Fixed Effects:

(Intr)

mppng_typ_c 0.039

**Block 4:**

Linear mixed model fit by REML. t-tests use Satterthwaite's method ['lmerModLmerTest']

Formula: log_rt ~ 1 + mapping_type_cc + (1 + mapping_type_cc | subject) +

(1 | word) + (1 + mapping_type_cc | target)

Data: LP_RT_4

REML criterion at convergence: 2538.7

Scaled residuals:

| Min | 1Q | Median | 3Q | Max |
| --- | --- | --- | --- | --- |
| -3.842 | -0.62 | -0.1762 | 0.5388 | 4.4066 |

Random effects:

| Groups | Name | Variance | Std.Dev. | Corr |
| --- | --- | --- | --- | --- |
| subject | (Intercept) | 0.058017 | 0.24087 |  |
|  | mapping_type_cc | 0.064028 | 0.25304 | 0.49 |
| target | (Intercept) | 0.005042 | 0.07101 |  |
|  | mapping_type_cc | 0.012174 | 0.11034 | 0.61 |
| word | (Intercept) | 0.00557 | 0.07463 |  |
| Residual |  | 0.142149 | 0.37703 |  |

Number of obs: 2494, groups: subject, 60; target, 27; word, 12

Fixed effects:

|  | Estimate | Std. Error | df | t value | Pr(>\|t\|) |  |
| --- | --- | --- | --- | --- | --- | --- |
| (Intercept) | 7.44054 | 0.04154 | 52.38162 | 179.126 | < 2e-16 | *** |
| mapping_type_cc | 0.44576 | 0.04508 | 50.07963 | 9.889 | 2.30E-13 | *** |

---

Signif. codes: 0 ‘***’ 0.001 ‘**’ 0.01 ‘*’ 0.05 ‘.’ 0.1 ‘ ’ 1

Correlation of Fixed Effects:

(Intr)

mppng_typ_c 0.423

**Block 5:**

Linear mixed model fit by REML. t-tests use Satterthwaite's method ['lmerModLmerTest']

Formula: log_rt ~ 1 + mapping_type_cc + (1 + mapping_type_cc | subject) +

(1 | target) + (1 | word)

Data: LP1_RT_5

REML criterion at convergence: 2336

Scaled residuals:

| Min | 1Q | Median | 3Q | Max |
| --- | --- | --- | --- | --- |
| -3.0199 | -0.6293 | -0.1552 | 0.5083 | 4.2813 |

Random effects:

| Groups | Name | Variance | Std.Dev. | Corr |
| --- | --- | --- | --- | --- |
| subject | (Intercept) | 0.064605 | 0.25418 |  |
|  | mapping_type_cc | 0.041791 | 0.20443 | 0.59 |
| target | (Intercept) | 0.005571 | 0.07464 |  |
| word | (Intercept) | 0.003623 | 0.06019 |  |
| Residual |  | 0.128542 | 0.35853 |  |

Number of obs: 2574, groups: subject, 60; target, 27; word, 12

Fixed effects:

|  | Estimate | Std. Error | df | t value | Pr(>\|t\|) |  |
| --- | --- | --- | --- | --- | --- | --- |
| (Intercept) | 7.38439 | 0.04074 | 68.15291 | 181.28 | <2e-16 | *** |
| mapping_type_cc | 0.36613 | 0.03315 | 76.1868 | 11.04 | <2e-16 | *** |

---

Signif. codes: 0 ‘***’ 0.001 ‘**’ 0.01 ‘*’ 0.05 ‘.’ 0.1 ‘ ’ 1

Correlation of Fixed Effects:

(Intr)

mppng_typ_c 0.429

**E2a:**

Linear mixed model fit by REML. t-tests use Satterthwaite's method ['lmerModLmerTest']

Formula: log_rt ~ 1 + mapping_type_cc + (1 | subject) + (1 | target)

Data: phase2_correct

REML criterion at convergence: 11132.9

Scaled residuals:

| Min | 1Q | Median | 3Q | Max |
| --- | --- | --- | --- | --- |
| -3.5343 | -0.6543 | -0.1274 | 0.5335 | 5.0034 |

Random effects:

| Groups | Name | Variance | Std.Dev. | Corr |
| --- | --- | --- | --- | --- |
| subject | (Intercept) | 0.128916 | 0.35905 |  |
|  | block_s | 0.009769 | 0.09884 | 0.1 |
| target | (Intercept) | 0.002615 | 0.05114 |  |
| word | (Intercept) | 0.003547 | 0.05956 |  |
| Residual |  | 0.148451 | 0.38529 |  |

Number of obs: 11329, groups: subject, 60; target, 26; word, 12

Fixed effects:

|  | Estimate | Std. Error | df | t value | Pr(>\|t\|) |  |
| --- | --- | --- | --- | --- | --- | --- |
| (Intercept) | 7.25E+00 | 5.07E-02 | 7.29E+01 | 143.145 | < 2e-16 | *** |
| mapping_type_cc | -3.74E-02 | 1.02E-02 | 2.38E+03 | -3.658 | 0.00026 | *** |
| block_s | -1.05E-01 | 1.31E-02 | 5.83E+01 | -8.007 | 5.74E-11 | *** |
| mapping_type_cc:block_s | 3.79E-03 | 5.70E-03 | 1.12E+04 | 0.665 | 0.50617 |  |

---

Signif. codes: 0 ‘***’ 0.001 ‘**’ 0.01 ‘*’ 0.05 ‘.’ 0.1 ‘ ’ 1

Correlation of Fixed Effects:

|  | (Intr) | mppn__ | blck_s |
| --- | --- | --- | --- |
| mppng_typ_c | 0.036 |  |  |
| block_s | 0.09 | -0.007 |  |
| mppng_ty_:_ | -0.003 | -0.142 | 0.064 |

**E2b:**

Linear mixed model fit by REML. t-tests use Satterthwaite's method ['lmerModLmerTest']

Formula: log_rt ~ 1 + mapping_type_cc + (1 | subject) + (1 | target)

Data: phase2_firstblock_correct

REML criterion at convergence: 1829.6

Scaled residuals:

| Min | 1Q | Median | 3Q | Max |
| --- | --- | --- | --- | --- |
| -2.9602 | -0.6578 | -0.0743 | 0.6227 | 3.9552 |

Random effects:

| Groups | Name | Variance | Std.Dev. |
| --- | --- | --- | --- |
| subject | (Intercept) | 0.176294 | 0.41987 |
| target | (Intercept) | 0.003463 | 0.05885 |
| Residual |  | 0.18596 | 0.43123 |

Number of obs: 1402, groups: subject, 60; target, 26

Fixed effects:

|  | Estimate | Std. Error | df | t value | Pr(>\|t\|) |  |
| --- | --- | --- | --- | --- | --- | --- |
| (Intercept) | 7.53783 | 0.05695 | 62.56894 | 132.37 | <2e-16 | *** |
| mapping_type_cc | -0.06624 | 0.02663 | 431.7548 | -2.488 | 0.0132 | * |

---

Signif. codes: 0 ‘***’ 0.001 ‘**’ 0.01 ‘*’ 0.05 ‘.’ 0.1 ‘ ’ 1

Correlation of Fixed Effects:

(Intr)

mppng_typ_c 0.061

**E2c:**

Linear mixed model fit by REML. t-tests use Satterthwaite's method ['lmerModLmerTest']

Formula: log_rt ~ 1 + mapping_type_cc + acc_1 + (1 | subject) + (1 | target)

Data: phase2_covariateRT

REML criterion at convergence: 1832.4

Scaled residuals:

| Min | 1Q | Median | 3Q | Max |
| --- | --- | --- | --- | --- |
| -2.9793 | -0.66 | -0.0688 | 0.6271 | 3.9135 |

Random effects:

| Groups | Name | Variance | Std.Dev. |
| --- | --- | --- | --- |
| subject | (Intercept) | 0.174716 | 0.41799 |
| target | (Intercept) | 0.003558 | 0.05965 |
| Residual |  | 0.185883 | 0.43114 |

Number of obs: 1402, groups: subject, 60; target, 26

Fixed effects:

|  | Estimate | Std. Error | df | t value | Pr(>\|t\|) |  |
| --- | --- | --- | --- | --- | --- | --- |
| (Intercept) | 7.4886 | 0.06783 | 125.5627 | 110.4 | <2e-16 | *** |
| mapping_type_cc | -0.0676 | 0.02668 | 435.2466 | -2.534 | 0.0116 | * |
| acc_1 | 0.05532 | 0.04172 | 1367.774 | 1.326 | 0.185 |  |

---

Signif. codes: 0 ‘***’ 0.001 ‘**’ 0.01 ‘*’ 0.05 ‘.’ 0.1 ‘ ’ 1

Correlation of Fixed Effects:

|  | (Intr) | mppn__ |
| --- | --- | --- |
| mppng_typ_c | 0.069 |  |
| acc_1 | -0.548 | -0.032 |

**Experiment 2**

**MEANS**

*Phase 1 Accuracy per mapping type*

| Mapping type | Mean accuracy | SD accuracy |
| --- | --- | --- |
| 1:1 | 0.80 | 0.40 |
| 1:2 | 0.67 | 0.47 |

*Phase 1 Accuracy per block*

| Block | Mean accuracy | SD accuracy |
| --- | --- | --- |
| 1 | 0.48 | 0.50 |
| 2 | 0.72 | 0.45 |
| 3 | 0.82 | 0.39 |
| 4 | 0.87 | 0.34 |
| 5 | 0.90 | 0.31 |

*Phase 1 Accuracy per mapping type and block*

| Mapping | 1:1 | | 1:2 | |
| --- | --- | --- | --- | --- |
| Block | Mean accuracy | SD accuracy | Mean accuracy | SD accuracy |
| 1 | 0.53 | 0.50 | 0.39 | 0.49 |
| 2 | 0.77 | 0.42 | 0.61 | 0.49 |
| 3 | 0.86 | 0.35 | 0.73 | 0.45 |
| 4 | 0.90 | 0.30 | 0.80 | 0.40 |
| 5 | 0.92 | 0.26 | 0.84 | 0.37 |

*Phase 2 Accuracy per mapping type*

| Mapping type | Mean accuracy | SD accuracy |
| --- | --- | --- |
| 1:1 | 0.78 | 0.41 |
| 1:2 | 0.80 | 0.40 |

*Phase 2 Accuracy per block*

| Block | Mean accuracy | SD accuracy |
| --- | --- | --- |
| 1 | 0.49 | 0.50 |
| 2 | 0.76 | 0.43 |
| 3 | 0.87 | 0.34 |
| 4 | 0.90 | 0.30 |
| 5 | 0.92 | 0.27 |

*Phase 2 Accuracy per mapping type and block*

| Mapping | 1:1 | | 1:2 | |
| --- | --- | --- | --- | --- |
| Block | Mean accuracy | SD accuracy | Mean accuracy | SD accuracy |
| 1 | 0.46 | 0.50 | 0.56 | 0.50 |
| 2 | 0.76 | 0.43 | 0.76 | 0.43 |
| 3 | 0.87 | 0.33 | 0.87 | 0.34 |
| 4 | 0.90 | 0.30 | 0.90 | 0.30 |
| 5 | 0.92 | 0.28 | 0.93 | 0.25 |

*Phase 1 RTs per mapping type*

| Mapping type | Mean accuracy (ms) | SD accuracy (ms) |
| --- | --- | --- |
| 1:1 | 1765 | 1087 |
| 1:2 | 2529 | 1471 |

*Phase 1 RTs per block*

| Block | Mean accuracy (ms) | SD accuracy (ms) |
| --- | --- | --- |
| 1 | 2646 | 1400 |
| 2 | 2273 | 1373 |
| 3 | 1944 | 1214 |
| 4 | 1813 | 1163 |
| 5 | 1720 | 1036 |

*Phase 1 RTs per mapping type and block*

| Mapping | 1:1 | | 1:2 | |
| --- | --- | --- | --- | --- |
| Block | Mean accuracy (ms) | SD accuracy (ms) | Mean accuracy (ms) | SD accuracy (ms) |
| 1 | 2563 | 1341 | 2874 | 1529 |
| 2 | 2088 | 1279 | 2743 | 1486 |
| 3 | 1692 | 1003 | 2546 | 1445 |
| 4 | 1520 | 818 | 2479 | 1507 |
| 5 | 1480 | 731 | 2250 | 1363 |

*Phase 2 RTs per mapping type*

| Mapping type | Mean accuracy (ms) | SD accuracy (ms) |
| --- | --- | --- |
| 1:1 | 1693 | 1050 |
| 1:2 | 1655 | 1037 |

*Phase 2 RTs per block*

| Block | Mean accuracy (ms) | SD accuracy (ms) |
| --- | --- | --- |
| 1 | 2359 | 1424 |
| 2 | 1874 | 1126 |
| 3 | 1627 | 978 |
| 4 | 1482 | 871 |
| 5 | 1407 | 725 |

*Phase 2 RTs per mapping type and block*

| Mapping | 1:1 | | 1:2 | |
| --- | --- | --- | --- | --- |
| Block | Mean accuracy (ms) | SD accuracy (ms) | Mean accuracy (ms) | SD accuracy (ms) |
| 1 | 2410 | 1450 | 2276 | 1377 |
| 2 | 1900 | 1140 | 1821 | 1095 |
| 3 | 1654 | 1013 | 1574 | 903 |
| 4 | 1474 | 829 | 1498 | 951 |
| 5 | 1419 | 718 | 1382 | 740 |

**Experiment 2:**

**GRAPHS:**

**RTs - LP1:**

**
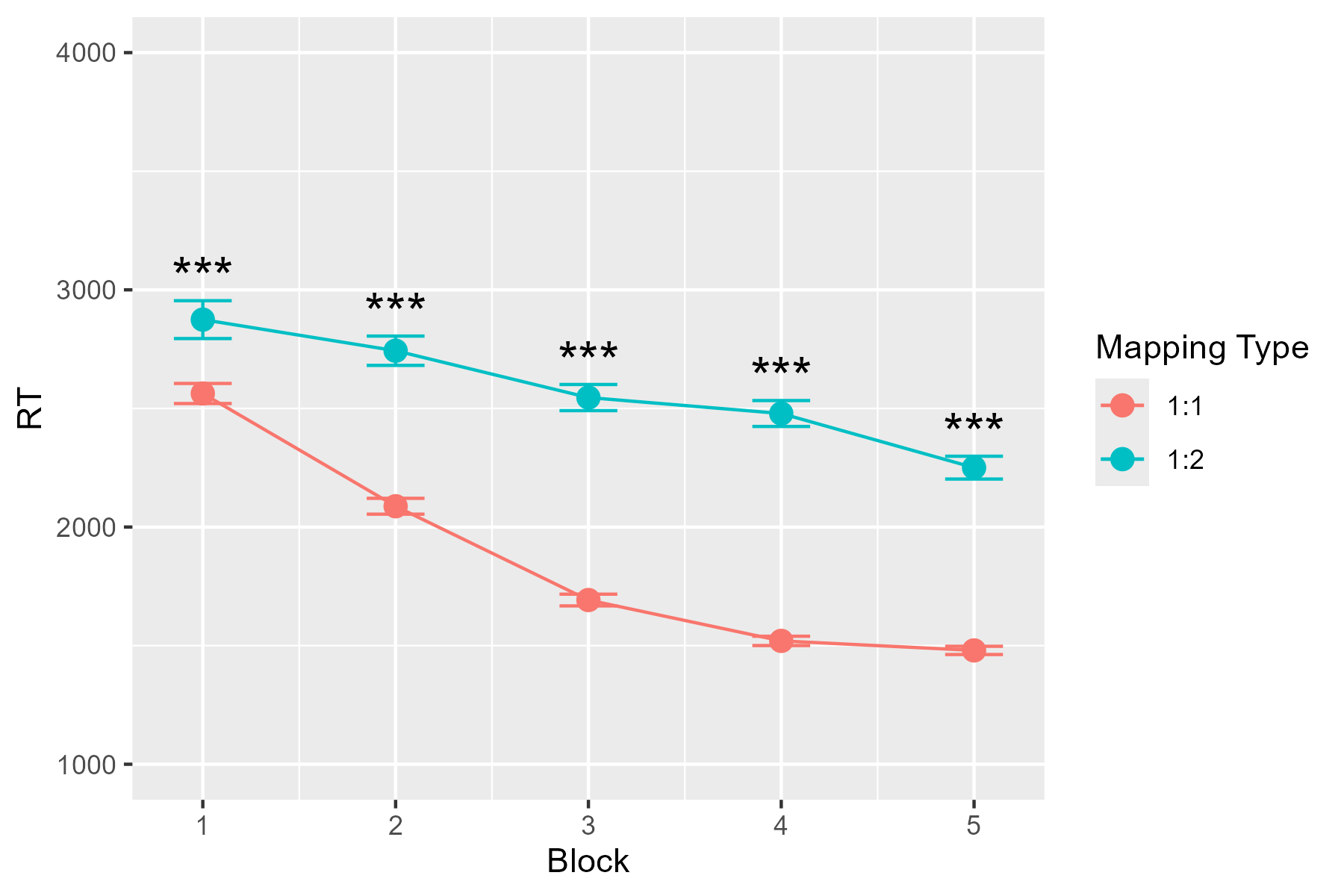
**

**RTs -LP2:**

**
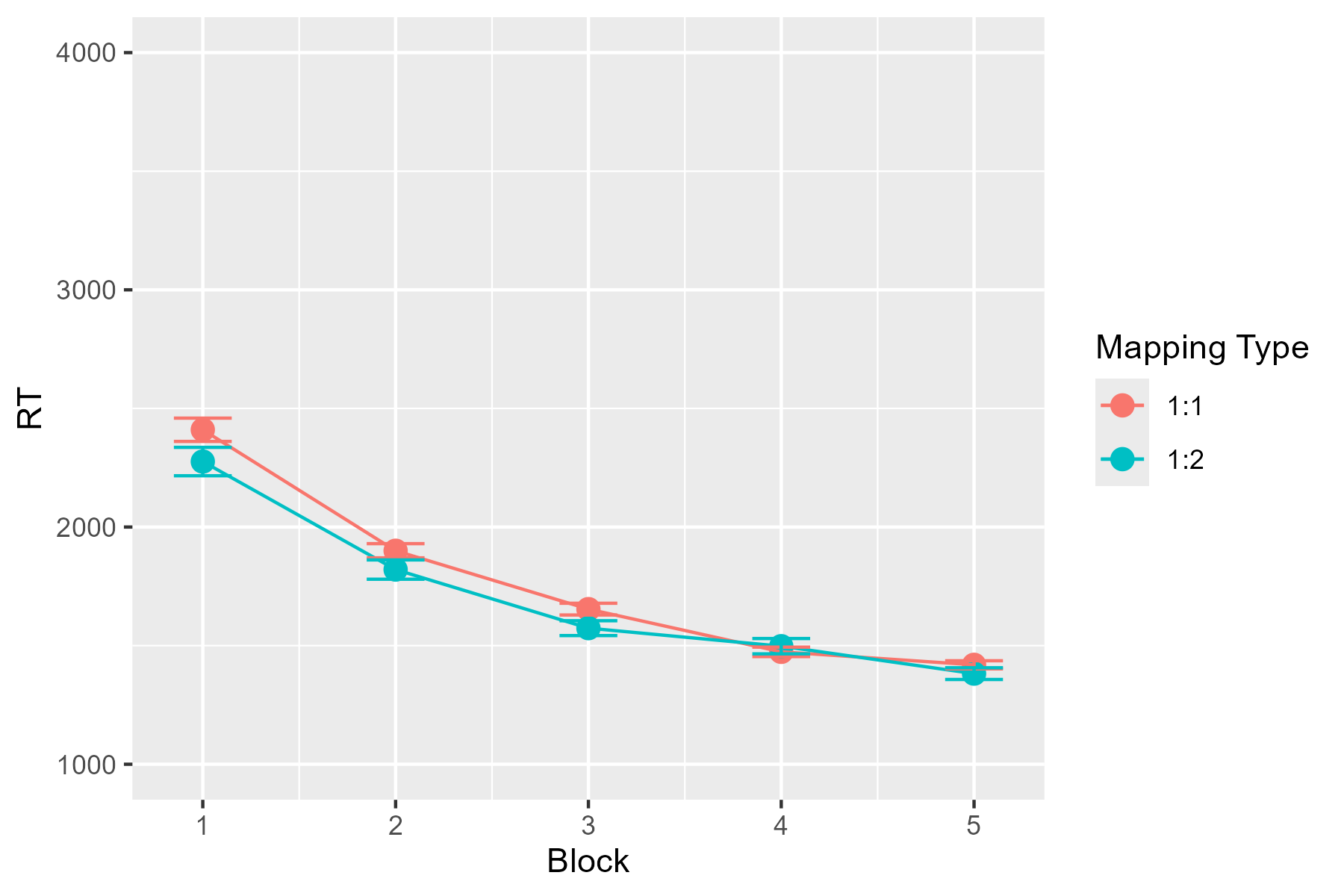
**

**Experiment 3**

**RESULTS**

**H1:**

Formula: accuracy ~ 1 + mapping_type_cc + block_s + mapping_type_cc:block_s +

(1 + block_s + mapping_type_cc | subject) + (1 + mapping_type_cc | target) +

(1 + mapping_type_cc + block_s | word)

Data: phase1

| AIC | BIC | logLik | deviance | df.resid |
| --- | --- | --- | --- | --- |
| 15874.8 | 16018.8 | -7918.4 | 15836.8 | 14381 |

Scaled residuals:

| Min | 1Q | Median | 3Q | Max |
| --- | --- | --- | --- | --- |
| -6.2617 | -0.8102 | 0.3144 | 0.7033 | 2.5077 |

Random effects:

| Groups | Name | Variance | Std.Dev. | Corr |  |
| --- | --- | --- | --- | --- | --- |
| subject | (Intercept) | 0.432457 | 0.65761 |  |  |
|  | block_s | 0.076072 | 0.27581 | 0.86 |  |
|  | mapping_type_cc | 0.446366 | 0.66811 | -0.73 | -0.58 |
| target | (Intercept) | 0.195098 | 0.4417 |  |  |
|  | mapping_type_cc | 0.361616 | 0.60135 | -0.44 |  |
| word | (Intercept) | 0.123468 | 0.35138 |  |  |
|  | mapping_type_cc | 0.273173 | 0.52266 | -0.29 |  |
|  | block_s | 0.004571 | 0.06761 | 0.18 | -0.23 |

Number of obs: 14400, groups: subject, 60; target, 29; word, 12

Fixed effects:

|  | Estimate | Std. Error | z value | Pr(>\|z\|) |  |
| --- | --- | --- | --- | --- | --- |
| (Intercept) | 0.86818 | 0.16187 | 5.363 | 8.17E-08 | *** |
| mapping_type_cc | -1.08337 | 0.22731 | -4.766 | 1.88E-06 | *** |
| block_s | 0.59208 | 0.04377 | 13.526 | < 2e-16 | *** |
| mapping_type_cc:block_s | -0.31754 | 0.03194 | -9.942 | < 2e-16 | *** |

---

Signif. codes: 0 ‘***’ 0.001 ‘**’ 0.01 ‘*’ 0.05 ‘.’ 0.1 ‘ ’ 1

Correlation of Fixed Effects:

|  | (Intr) | mppn__ | blck_s |
| --- | --- | --- | --- |
| mppng_typ_c | -0.441 |  |  |
| block_s | 0.44 | -0.264 |  |
| mppng_ty_:_ | -0.03 | 0.063 | -0.089 |

**Post – hoc (Mapping type * Block)**

**Block 1:**

Generalized linear mixed model fit by maximum likelihood (Laplace Approximation) ['glmerMod']

Family: binomial ( logit )

Formula: accuracy ~ 1 + mapping_type_cc + (1 + mapping_type_cc | target) +

(1 + mapping_type_cc | subject)

Data: get(lp_var)

| AIC | BIC | logLik | deviance | df.resid |
| --- | --- | --- | --- | --- |
| 3844 | 3891.8 | -1914 | 3828 | 2872 |

Scaled residuals:

| Min | 1Q | Median | 3Q | Max |
| --- | --- | --- | --- | --- |
| -1.3952 | -0.8169 | -0.6217 | 1.0474 | 1.8949 |

Random effects:

| Groups | Name | Variance | Std.Dev. | Corr |
| --- | --- | --- | --- | --- |
| subject | (Intercept) | 0.08401 | 0.2899 |  |
|  | mapping_type_cc | 0.19312 | 0.4395 | -0.28 |
| target | (Intercept) | 0.05278 | 0.2297 |  |
|  | mapping_type_cc | 0.48572 | 0.6969 | 0.06 |

Number of obs: 2880, groups: subject, 60; target, 29

Fixed effects:

|  | Estimate | Std. Error | z value | Pr(>\|z\|) |  |
| --- | --- | --- | --- | --- | --- |
| (Intercept) | -0.41634 | 0.07612 | -5.47 | 4.51E-08 | *** |
| mapping_type_cc | -0.25735 | 0.17122 | -1.503 | 0.133 |  |

---

Signif. codes: 0 ‘***’ 0.001 ‘**’ 0.01 ‘*’ 0.05 ‘.’ 0.1 ‘ ’ 1

Correlation of Fixed Effects:

(Intr)

mppng_typ_c -0.046

**Block 2:**

Generalized linear mixed model fit by maximum likelihood (Laplace Approximation) ['glmerMod']

Family: binomial ( logit )

Formula: accuracy ~ 1 + mapping_type_cc + (1 | target) + (1 + mapping_type_cc | subject) +

(1 | word)

Data: get(lp_var)

| AIC | BIC | logLik | deviance | df.resid |
| --- | --- | --- | --- | --- |
| 3753.7 | 3795.4 | -1869.8 | 3739.7 | 2873 |

Scaled residuals:

| Min | 1Q | Median | 3Q | Max |
| --- | --- | --- | --- | --- |
| -2.462 | -0.9281 | 0.502 | 0.8241 | 1.7466 |

Random effects:

| Groups | Name | Variance | Std.Dev. | Corr |
| --- | --- | --- | --- | --- |
| subject | (Intercept) | 0.20672 | 0.4547 |  |
|  | mapping_type_cc | 0.30034 | 0.548 | -0.65 |
| target | (Intercept) | 0.16766 | 0.4095 |  |
| word | (Intercept) | 0.07289 | 0.27 |  |

Number of obs: 2880, groups: subject, 60; target, 29; word, 12

Fixed effects:

|  | Estimate | Std. Error | z value | Pr(>\|z\|) |  |
| --- | --- | --- | --- | --- | --- |
| (Intercept) | 0.3083 | 0.1305 | 2.362 | 0.0182 | * |
| mapping_type_cc | -0.6628 | 0.1222 | -5.422 | 5.89E-08 | *** |

---

Signif. codes: 0 ‘***’ 0.001 ‘**’ 0.01 ‘*’ 0.05 ‘.’ 0.1 ‘ ’ 1

Correlation of Fixed Effects:

(Intr)

mppng_typ_c -0.194

**Block 3:**

Generalized linear mixed model fit by maximum likelihood (Laplace Approximation) ['glmerMod']

Family: binomial ( logit )

Formula: accuracy ~ 1 + mapping_type_cc + (1 + mapping_type_cc | subject) +

(1 + mapping_type_cc | target) + (1 + mapping_type_cc | word)

Data: get(lp_var)

| AIC | BIC | logLik | deviance | df.resid |
| --- | --- | --- | --- | --- |
| 3361.9 | 3427.5 | -1669.9 | 3339.9 | 2869 |

Scaled residuals:

| Min | 1Q | Median | 3Q | Max |
| --- | --- | --- | --- | --- |
| -7.825 | -0.88 | 0.3526 | 0.7402 | 2.5329 |

Random effects:

| Groups | Name | Variance | Std.Dev. | Corr |
| --- | --- | --- | --- | --- |
| subject | (Intercept) | 0.61884 | 0.7867 |  |
|  | mapping_type_cc | 0.98604 | 0.993 | -0.94 |
| target | (Intercept) | 0.22208 | 0.4713 |  |
|  | mapping_type_cc | 0.75069 | 0.8664 | -0.56 |
| word | (Intercept) | 0.07554 | 0.2748 |  |
|  | mapping_type_cc | 0.44419 | 0.6665 | -0.49 |

Number of obs: 2880, groups: subject, 60; target, 29; word, 12

Fixed effects:

|  | Estimate | Std. Error | z value | Pr(>\|z\|) |  |
| --- | --- | --- | --- | --- | --- |
| (Intercept) | 0.8958 | 0.1725 | 5.193 | 2.07E-07 | *** |
| mapping_type_cc | -1.3921 | 0.3192 | -4.362 | 1.29E-05 | *** |

---

Signif. codes: 0 ‘***’ 0.001 ‘**’ 0.01 ‘*’ 0.05 ‘.’ 0.1 ‘ ’ 1

Correlation of Fixed Effects:

(Intr)

mppng_typ_c -0.619

**Block 4:**

Generalized linear mixed model fit by maximum likelihood (Laplace Approximation) ['glmerMod']

Family: binomial ( logit )

Formula: accuracy ~ 1 + mapping_type_cc + (1 + mapping_type_cc | subject) +

(1 + mapping_type_cc | target) + (1 | word)

Data: get(lp_var)

| AIC | BIC | logLik | deviance | df.resid |
| --- | --- | --- | --- | --- |
| 2831.6 | 2885.3 | -1406.8 | 2813.6 | 2871 |

Scaled residuals:

| Min | 1Q | Median | 3Q | Max |
| --- | --- | --- | --- | --- |
| -6.8203 | 0.0844 | 0.3226 | 0.5931 | 1.429 |

Random effects:

| Groups | Name | Variance | Std.Dev. | Corr |
| --- | --- | --- | --- | --- |
| subject | (Intercept) | 1.02874 | 1.0143 |  |
|  | mapping_type_cc | 1.43818 | 1.1992 | -0.85 |
| target | (Intercept) | 0.29252 | 0.5409 |  |
|  | mapping_type_cc | 0.25041 | 0.5004 | -0.69 |
| word | (Intercept) | 0.05855 | 0.242 |  |

Number of obs: 2880, groups: subject, 60; target, 29; word, 12

Fixed effects:

|  | Estimate | Std. Error | z value | Pr(>\|z\|) |  |
| --- | --- | --- | --- | --- | --- |
| (Intercept) | 1.6363 | 0.1959 | 8.352 | < 2e-16 | *** |
| mapping_type_cc | -1.6746 | 0.2499 | -6.702 | 2.05E-11 | *** |

---

Signif. codes: 0 ‘***’ 0.001 ‘**’ 0.01 ‘*’ 0.05 ‘.’ 0.1 ‘ ’ 1

Correlation of Fixed Effects:

(Intr)

mppng_typ_c -0.663

**Block 5:**

Generalized linear mixed model fit by maximum likelihood (Laplace Approximation) ['glmerMod']

Family: binomial ( logit )

Formula: accuracy ~ 1 + mapping_type_cc + (1 + mapping_type_cc | subject) +

(1 + mapping_type_cc | target)

Data: get(lp_var)

| AIC | BIC | logLik | deviance | df.resid |
| --- | --- | --- | --- | --- |
| 2424 | 2471.7 | -1204 | 2408 | 2872 |

Scaled residuals:

| Min | 1Q | Median | 3Q | Max |
| --- | --- | --- | --- | --- |
| -6.0035 | 0.056 | 0.2546 | 0.518 | 1.6986 |

Random effects:

| Groups | Name | Variance | Std.Dev. | Corr |
| --- | --- | --- | --- | --- |
| subject | (Intercept) | 2.1327 | 1.4604 |  |
|  | mapping_type_cc | 2.2034 | 1.4844 | -0.96 |
| target | (Intercept) | 0.1848 | 0.4298 |  |
|  | mapping_type_cc | 1.3846 | 1.1767 | -0.47 |

Number of obs: 2880, groups: subject, 60; target, 29

Fixed effects:

|  | Estimate | Std. Error | z value | Pr(>\|z\|) |  | |
| --- | --- | --- | --- | --- | --- | --- |
| (Intercept) | 2.1774 | 0.2387 | 9.123 | < 2e-16 | *** |  |
| mapping_type_cc | -2.2456 | 0.3734 | -6.014 | 1.81E-09 | *** | |

---

Signif. codes: 0 ‘***’ 0.001 ‘**’ 0.01 ‘*’ 0.05 ‘.’ 0.1 ‘ ’ 1

Correlation of Fixed Effects:

(Intr)

mppng_typ_c -0.733

**H2a:**

Formula: accuracy ~ 1 + mapping_type_cc + block_s + mapping_type_cc:block_s +

(1 + block_s | subject) + (1 | word) + (1 | target)

Data: phase2

| AIC | BIC | logLik | deviance | df.resid |
| --- | --- | --- | --- | --- |
| 11005.6 | 11073.8 | -5493.8 | 10987.6 | 14391 |

Scaled residuals:

| Min | 1Q | Median | 3Q | Max |
| --- | --- | --- | --- | --- |
| -75.268 | 0.011 | 0.165 | 0.481 | 2.237 |

Random effects:

| Groups | Name | Variance | Std.Dev. | Corr |
| --- | --- | --- | --- | --- |
| subject | (Intercept) | 3.45381 | 1.8584 |  |
|  | block_s | 0.51608 | 0.7184 | 0.96 |
| target | (Intercept) | 0.0765 | 0.2766 |  |
| word | (Intercept) | 0.09663 | 0.3109 |  |

Number of obs: 14400, groups: subject, 60; target, 28; word, 12

Fixed effects:

|  | Estimate | Std. Error | z value | Pr(>\|z\|) |  |
| --- | --- | --- | --- | --- | --- |
| (Intercept) | 2.51392 | 0.27107 | 9.274 | < 2e-16 | *** |
| mapping_type_cc | 0.24175 | 0.06997 | 3.455 | 0.00055 | *** |
| block_s | 1.18466 | 0.101 | 11.729 | < 2e-16 | *** |
| mapping_type_cc:block_s | 0.04595 | 0.03682 | 1.248 | 0.21203 |  |

---

Signif. codes: 0 ‘***’ 0.001 ‘**’ 0.01 ‘*’ 0.05 ‘.’ 0.1 ‘ ’ 1

Correlation of Fixed Effects:

|  | (Intr) | mppn__ | blck_s |
| --- | --- | --- | --- |
| mppng_typ_c | 0.011 |  |  |
| block_s | 0.874 | 0.01 |  |
| mppng_ty_:_ | 0.005 | 0.369 | 0.01 |

**H2b:**

Formula: accuracy ~ 1 + mapping_type_cc + (1 + mapping_type_cc | subject) +

(1 | word) + (1 | target)

Data: phase2_firstblock

| AIC | BIC | logLik | deviance | df.resid |
| --- | --- | --- | --- | --- |
| 3838.5 | 3880.2 | -1912.2 | 3824.5 | 2873 |

Scaled residuals:

| Min | 1Q | Median | 3Q | Max |
| --- | --- | --- | --- | --- |
| -2.5415 | -0.9009 | 0.4973 | 0.8676 | 2.3481 |

Random effects:

| Groups | Name | Variance | Std.Dev. | Corr |
| --- | --- | --- | --- | --- |
| subject | (Intercept) | 0.33605 | 0.5797 |  |
|  | mapping_type_cc | 0.27569 | 0.5251 | -0.26 |
| target | (Intercept) | 0.09764 | 0.3125 |  |
| word | (Intercept) | 0.08228 | 0.2868 |  |

Number of obs: 2880, groups: subject, 60; target, 28; word, 12

Fixed effects:

|  | Estimate | Std. Error | z value | Pr(>\|z\|) |
| --- | --- | --- | --- | --- |
| (Intercept) | 0.1059 | 0.1336 | 0.792 | 0.428 |
| mapping_type_cc | 0.1508 | 0.1213 | 1.244 | 0.214 |

Correlation of Fixed Effects:

(Intr)

mppng_typ_c -0.075

**H2c:**

Formula: accuracy ~ 1 + mapping_type_cc + acc_1 + (1 + mapping_type_cc | subject) + (1 | word)

+ (1 | target)

Data: phase2_covariate

| AIC | BIC | logLik | deviance | df.resid |
| --- | --- | --- | --- | --- |
| 3838.9 | 3886.7 | -1911.5 | 3822.9 | 2872 |

Scaled residuals:

| Min | 1Q | Median | 3Q | Max |
| --- | --- | --- | --- | --- |
| -2.5578 | -0.9045 | 0.4994 | 0.8724 | 2.3281 |

Random effects:

| Groups | Name | Variance | Std.Dev. | Corr |
| --- | --- | --- | --- | --- |
| subject | (Intercept) | 0.33298 | 0.577 |  |
|  | mapping_type_cc | 0.27746 | 0.5267 | -0.28 |
| target | (Intercept) | 0.09945 | 0.3154 |  |
| word | (Intercept) | 0.08253 | 0.2873 |  |

Number of obs: 2880, groups: subject, 60; target, 28; word, 12

Fixed effects:

|  | Estimate | Std. Error | z value | Pr(>\|z\|) |
| --- | --- | --- | --- | --- |
| (Intercept) | 0.0014 | 0.1583 | 0.009 | 0.993 |
| mapping_type_cc | 0.1585 | 0.1217 | 1.302 | 0.193 |
| acc_1 | 0.1311 | 0.1063 | 1.234 | 0.217 |

Correlation of Fixed Effects:

|  | (Intr) | mppn__ |
| --- | --- | --- |
| mppng_typ_c | -0.094 |  |
| acc_1 | -0.535 | 0.052 |

**E1:**

Linear mixed model fit by REML. t-tests use Satterthwaite's method ['lmerModLmerTest']

Formula: log_rt ~ 1 + mapping_type_cc + block_s + mapping_type_cc:block_s +

(1 + mapping_type_cc + block_s | subject) + (1 + mapping_type_cc | target)

Data: phase1_correct

REML criterion at convergence: 10888.1

Scaled residuals:

| Min | 1Q | Median | 3Q | Max |
| --- | --- | --- | --- | --- |
| -3.2381 | -0.6659 | -0.1094 | 0.5714 | 4.3613 |

Random effects:

| Groups | Name | Variance | Std.Dev. | Corr |  |
| --- | --- | --- | --- | --- | --- |
| subject | (Intercept) | 0.056266 | 0.2372 |  |  |
|  | mapping_type_cc | 0.042661 | 0.20654 | 0.56 |  |
|  | block_s | 0.00342 | 0.05848 | 0.04 | -0.07 |
| target | (Intercept) | 0.005152 | 0.07178 |  |  |
|  | mapping_type_cc | 0.012147 | 0.11021 | -0.81 |  |
| Residual |  | 0.183318 | 0.42816 |  |  |

Number of obs: 9054, groups: subject, 60; target, 29

Fixed effects:

|  | Estimate | Std. Error | df | t value | Pr(>\|t\|) |  |
| --- | --- | --- | --- | --- | --- | --- |
| (Intercept) | 7.63E+00 | 3.42E-02 | 7.58E+01 | 223.175 | < 2e-16 | *** |
| mapping_type_cc | 2.83E-01 | 3.71E-02 | 5.60E+01 | 7.615 | 3.35E-10 | *** |
| block_s | -8.92E-02 | 8.27E-03 | 5.93E+01 | -10.783 | 1.32E-15 | *** |
| mapping_type_cc:block_s | 7.69E-02 | 6.73E-03 | 8.90E+03 | 11.433 | < 2e-16 | *** |

---

Signif. codes: 0 ‘***’ 0.001 ‘**’ 0.01 ‘*’ 0.05 ‘.’ 0.1 ‘ ’ 1

Correlation of Fixed Effects:

|  | (Intr) | mppn__ | blck_s |
| --- | --- | --- | --- |
| mppng_typ_c | 0.144 |  |  |
| block_s | 0.018 | -0.045 |  |
| mppng_ty_:_ | -0.001 | -0.058 | 0.038 |

**Post – hoc (Mapping type * Block)**

**Block 1:**

Linear mixed model fit by REML. t-tests use Satterthwaite's method ['lmerModLmerTest']

Formula: log_rt ~ 1 + mapping_type_cc + (1 | subject) + (1 | word)

Data: LP1_RT_1

REML criterion at convergence: 1260.7

Scaled residuals:

| Min | 1Q | Median | 3Q | Max |
| --- | --- | --- | --- | --- |
| -3.2262 | -0.6706 | -0.0506 | 0.5799 | 3.1483 |

Random effects:

| Groups | Name | Variance | Std.Dev. |
| --- | --- | --- | --- |
| subject | (Intercept) | 0.05684 | 0.2384 |
| word | (Intercept) | 0.00246 | 0.0496 |
| Residual |  | 0.15094 | 0.3885 |

Number of obs: 1178, groups: subject, 60; word, 12

Fixed effects:

|  | Estimate | Std. Error | df | t value | Pr(>\|t\|) |  |
| --- | --- | --- | --- | --- | --- | --- |
| (Intercept) | 7.84E+00 | 3.59E-02 | 5.51E+01 | 218.461 | <2e-16 | *** |
| mapping_type_cc | 6.00E-02 | 2.38E-02 | 1.05E+03 | 2.518 | 0.0119 | * |

---

Signif. codes: 0 ‘***’ 0.001 ‘**’ 0.01 ‘*’ 0.05 ‘.’ 0.1 ‘ ’ 1

Correlation of Fixed Effects:

(Intr)

mppng_typ_c 0.017

**Block 2:**

Linear mixed model fit by REML. t-tests use Satterthwaite's method ['lmerModLmerTest']

Formula: log_rt ~ 1 + mapping_type_cc + (1 + mapping_type_cc | subject) +

(1 + mapping_type_cc | target) + (1 | word)

Data: LP1_RT_2

REML criterion at convergence: 2054.9

Scaled residuals:

| Min | 1Q | Median | 3Q | Max |
| --- | --- | --- | --- | --- |
| -2.5871 | -0.7011 | -0.1002 | 0.6266 | 3.9257 |

Random effects:

| Groups | Name | Variance | Std.Dev. | Corr |
| --- | --- | --- | --- | --- |
| subject | (Intercept) | 0.075479 | 0.27473 |  |
|  | mapping_type_cc | 0.021262 | 0.14582 | 0.22 |
| target | (Intercept) | 0.004314 | 0.06568 |  |
|  | mapping_type_cc | 0.025178 | 0.15867 | -0.53 |
| word | (Intercept) | 0.000231 | 0.0152 |  |
| Residual |  | 0.180613 | 0.42499 |  |

Number of obs: 1618, groups: subject, 60; target, 29; word, 12

Fixed effects:

|  | Estimate | Std. Error | df | t value | Pr(>\|t\|) |  |
| --- | --- | --- | --- | --- | --- | --- |
| (Intercept) | 7.67821 | 0.04015 | 55.92759 | 191.262 | < 2e-16 | *** |
| mapping_type_cc | 0.20471 | 0.04381 | 24.97334 | 4.672 | 8.73E-05 | *** |

---

Signif. codes: 0 ‘***’ 0.001 ‘**’ 0.01 ‘*’ 0.05 ‘.’ 0.1 ‘ ’ 1

Correlation of Fixed Effects:

(Intr)

mppng_typ_c -0.041

**Block 3:**

Linear mixed model fit by REML. t-tests use Satterthwaite's method ['lmerModLmerTest']

Formula: log_rt ~ 1 + mapping_type_cc + (1 + mapping_type_cc | subject) +

(1 | word) + (1 + mapping_type_cc | target)

Data: LP1_RT_3

REML criterion at convergence: 2418

Scaled residuals:

| Min | 1Q | Median | 3Q | Max |
| --- | --- | --- | --- | --- |
| -2.8736 | -0.635 | -0.1025 | 0.5289 | 3.5088 |

Random effects:

| Groups | Name | Variance | Std.Dev. | Corr |
| --- | --- | --- | --- | --- |
| subject | (Intercept) | 0.084096 | 0.28999 |  |
|  | mapping_type_cc | 0.062255 | 0.24951 | 0.59 |
| target | (Intercept) | 0.006325 | 0.07953 |  |
|  | mapping_type_cc | 0.006142 | 0.07837 | -0.96 |
| word | (Intercept) | 0.004556 | 0.0675 |  |
| Residual |  | 0.189878 | 0.43575 |  |

Number of obs: 1828, groups: subject, 60; target, 29; word, 12

Fixed effects:

|  | Estimate | Std. Error | df | t value | Pr(>\|t\|) |  |
| --- | --- | --- | --- | --- | --- | --- |
| (Intercept) | 7.6178 | 0.04621 | 62.24471 | 164.857 | < 2e-16 | *** |
| mapping_type_cc | 0.33468 | 0.04439 | 44.99315 | 7.539 | 1.63E-09 | *** |

---

Signif. codes: 0 ‘***’ 0.001 ‘**’ 0.01 ‘*’ 0.05 ‘.’ 0.1 ‘ ’ 1

Correlation of Fixed Effects:

(Intr)

mppng_typ_c 0.232

**Block 4:**

Linear mixed model fit by REML. t-tests use Satterthwaite's method ['lmerModLmerTest']

Formula: log_rt ~ 1 + mapping_type_cc + (1 + mapping_type_cc | subject) +

(1 + mapping_type_cc | target)

Data: LP1_RT_4

REML criterion at convergence: 2597.4

Scaled residuals:

| Min | 1Q | Median | 3Q | Max |
| --- | --- | --- | --- | --- |
| -2.9627 | -0.6538 | -0.1235 | 0.5164 | 4.3742 |

Random effects:

| Groups | Name | Variance | Std.Dev. | Corr |
| --- | --- | --- | --- | --- |
| subject | (Intercept) | 0.06574 | 0.2564 |  |
|  | mapping_type_cc | 0.06392 | 0.25283 | 0.52 |
| target | (Intercept) | 0.00728 | 0.08532 |  |
|  | mapping_type_cc | 0.01297 | 0.11388 | -0.82 |
| Residual |  | 0.17133 | 0.41392 |  |

Number of obs: 2152, groups: subject, 60; target, 29

Fixed effects:

|  | Estimate | Std. Error | df | t value | Pr(>\|t\|) |  |
| --- | --- | --- | --- | --- | --- | --- |
| (Intercept) | 7.53279 | 0.03833 | 71.56463 | 196.51 | < 2e-16 | *** |
| mapping_type_cc | 0.40567 | 0.04568 | 47.04177 | 8.88 | 1.27E-11 | *** |

---

Signif. codes: 0 ‘***’ 0.001 ‘**’ 0.01 ‘*’ 0.05 ‘.’ 0.1 ‘ ’ 1

Correlation of Fixed Effects:

(Intr)

mppng_typ_c 0.135

**Block 5:**

Linear mixed model fit by REML. t-tests use Satterthwaite's method ['lmerModLmerTest']

Formula: log_rt ~ 1 + mapping_type_cc + (1 + mapping_type_cc | subject) +

(1 + mapping_type_cc | word) + (1 | target)

Data: LP1_RT_5

REML criterion at convergence: 2698.1

Scaled residuals:

| Min | 1Q | Median | 3Q | Max |
| --- | --- | --- | --- | --- |
| -2.8476 | -0.6272 | -0.1239 | 0.4789 | 4.0047 |

Random effects:

| Groups | Name | Variance | Std.Dev. | Corr |
| --- | --- | --- | --- | --- |
| subject | (Intercept) | 0.069073 | 0.26282 |  |
|  | mapping_type_cc | 0.043612 | 0.20883 | 0.58 |
| target | (Intercept) | 0.003292 | 0.05738 |  |
| word | (Intercept) | 0.00667 | 0.08167 |  |
|  | mapping_type_cc | 0.012365 | 0.1112 | -0.12 |
| Residual |  | 0.168554 | 0.41055 |  |

Number of obs: 2278, groups: subject, 60; target, 29; word, 12

Fixed effects:

|  | Estimate | Std. Error | df | t value | Pr(>\|t\|) |  |
| --- | --- | --- | --- | --- | --- | --- |
| (Intercept) | 7.47132 | 0.04371 | 50.38445 | 170.944 | < 2e-16 | *** |
| mapping_type_cc | 0.36657 | 0.04708 | 21.76511 | 7.786 | 9.94E-08 | *** |

---

Signif. codes: 0 ‘***’ 0.001 ‘**’ 0.01 ‘*’ 0.05 ‘.’ 0.1 ‘ ’ 1

Correlation of Fixed Effects:

(Intr)

mppng_typ_c 0.220

**E2a:**

Linear mixed model fit by REML. t-tests use Satterthwaite's method ['lmerModLmerTest']

Formula: log_rt ~ 1 + mapping_type_cc + block_s + mapping_type_cc:block_s +

(1 + block_s + mapping_type_cc | subject) + (1 | target) +

(1 | word)

Data: phase2_correct

REML criterion at convergence: 11358.2

Scaled residuals:

| Min | 1Q | Median | 3Q | Max |
| --- | --- | --- | --- | --- |
| -3.7858 | -0.6503 | -0.1306 | 0.518 | 5.3148 |

Random effects:

| Groups | Name | Variance | Std.Dev. | Corr |  |
| --- | --- | --- | --- | --- | --- |
| subject | (Intercept) | 0.096322 | 0.31036 |  |  |
|  | block_s | 0.004865 | 0.06975 | -0.04 |  |
|  | mapping_type_cc | 0.005768 | 0.07595 | -0.11 | 0.11 |
| target | (Intercept) | 0.006202 | 0.07875 |  |  |
| word | (Intercept) | 0.002518 | 0.05018 |  |  |
| Residual |  | 0.154075 | 0.39252 |  |  |

Number of obs: 11114, groups: subject, 60; target, 28; word, 12

Fixed effects:

|  | Estimate | Std. Error | df | t value | Pr(>\|t\|) |  |
| --- | --- | --- | --- | --- | --- | --- |
| (Intercept) | 7.32E+00 | 4.54E-02 | 7.92E+01 | 161.321 | < 2e-16 | *** |
| mapping_type_cc | -2.84E-02 | 1.51E-02 | 1.10E+02 | -1.879 | 0.0629 | . |
| block_s | -1.03E-01 | 9.44E-03 | 5.72E+01 | -10.912 | 1.35E-15 | *** |
| mapping_type_cc:block_s | -5.07E-03 | 5.56E-03 | 1.10E+04 | -0.912 | 0.3616 |  |

---

Signif. codes: 0 ‘***’ 0.001 ‘**’ 0.01 ‘*’ 0.05 ‘.’ 0.1 ‘ ’ 1

Correlation of Fixed Effects:

|  | (Intr) | mppn__ | blck_s |
| --- | --- | --- | --- |
| mppng_typ_c | -0.061 |  |  |
| block_s | -0.037 | 0.069 |  |
| mppng_ty_:_ | 0.001 | -0.095 | -0.007 |

**E2b:**

Linear mixed model fit by REML. t-tests use Satterthwaite's method ['lmerModLmerTest']

Formula: log_rt ~ 1 + mapping_type_cc + (1 | subject) + (1 | word)

Data: phase2_firstblock_correct

REML criterion at convergence: 2007.4

Scaled residuals:

| Min | 1Q | Median | 3Q | Max |
| --- | --- | --- | --- | --- |
| -2.9644 | -0.6802 | -0.0759 | 0.5948 | 3.5631 |

Random effects:

| Groups | Name | Variance | Std.Dev. |
| --- | --- | --- | --- |
| subject | (Intercept) | 0.126174 | 0.35521 |
| word | (Intercept) | 0.003127 | 0.05592 |
| Residual |  | 0.204596 | 0.45232 |

Number of obs: 1458, groups: subject, 60; word, 12

Fixed effects:

|  | Estimate | Std. Error | df | t value | Pr(>\|t\|) |  |
| --- | --- | --- | --- | --- | --- | --- |
| (Intercept) | 7.61E+00 | 5.02E-02 | 6.18E+01 | 151.597 | <2e-16 | *** |
| mapping_type_cc | 1.75E-03 | 2.47E-02 | 1.32E+03 | 0.071 | 0.943 |  |

---

Signif. codes: 0 ‘***’ 0.001 ‘**’ 0.01 ‘*’ 0.05 ‘.’ 0.1 ‘ ’ 1

Correlation of Fixed Effects:

(Intr)

mppng_typ_c -0.007

**E2c:**

Linear mixed model fit by REML. t-tests use Satterthwaite's method ['lmerModLmerTest']

Formula: log_rt ~ 1 + mapping_type_cc + acc_1 + (1 | subject) + (1 | word)

Data: phase2_covariateRT

REML criterion at convergence: 2010.9

Scaled residuals:

| Min | 1Q | Median | 3Q | Max |
| --- | --- | --- | --- | --- |
| -2.94 | -0.6845 | -0.0778 | 0.5894 | 3.5615 |

Random effects:

| Groups | Name | Variance | Std.Dev. |
| --- | --- | --- | --- |
| subject | (Intercept) | 0.126892 | 0.3562 |
| word | (Intercept) | 0.003227 | 0.0568 |
| Residual |  | 0.204461 | 0.4522 |

Number of obs: 1458, groups: subject, 60; word, 12

Fixed effects:

|  | Estimate | Std. Error | df | t value | Pr(>\|t\|) |  |
| --- | --- | --- | --- | --- | --- | --- |
| (Intercept) | 7.64E+00 | 5.71E-02 | 1.01E+02 | 133.71 | <2e-16 | *** |
| mapping_type_cc | -4.21E-04 | 2.47E-02 | 1.32E+03 | -0.017 | 0.986 |  |
| acc_1 | -3.91E-02 | 3.32E-02 | 1.42E+03 | -1.18 | 0.238 |  |

---

Signif. codes: 0 ‘***’ 0.001 ‘**’ 0.01 ‘*’ 0.05 ‘.’ 0.1 ‘ ’ 1

Correlation of Fixed Effects:

|  | (Intr) | mppn__ |
| --- | --- | --- |
| mppng_typ_c | -0.041 |  |
| acc_1 | -0.472 | 0.073 |

**Experiment 3**

**MEANS**

*Phase 1 Accuracy per mapping type*

| Mapping type | Mean accuracy | SD accuracy |
| --- | --- | --- |
| 1:1 | 0.71 | 0.45 |
| 1:2 | 0.57 | 0.50 |

*Phase 1 Accuracy per block*

| Block | Mean accuracy | SD accuracy |
| --- | --- | --- |
| 1 | 0.41 | 0.49 |
| 2 | 0.57 | 0.50 |
| 3 | 0.65 | 0.48 |
| 4 | 0.76 | 0.43 |
| 5 | 0.80 | 0.40 |

*Phase 1 Accuracy per mapping type and block*

| Mapping | 1:1 | | 1:2 | |
| --- | --- | --- | --- | --- |
| Block | Mean accuracy | SD accuracy | Mean accuracy | SD accuracy |
| 1 | 0.44 | 0.50 | 0.39 | 0.49 |
| 2 | 0.64 | 0.48 | 0.49 | 0.50 |
| 3 | 0.74 | 0.44 | 0.56 | 0.50 |
| 4 | 0.84 | 0.36 | 0.67 | 0.47 |
| 5 | 0.88 | 0.33 | 0.73 | 0.45 |

*Phase 2 Accuracy per mapping type*

| Mapping type | Mean accuracy | SD accuracy |
| --- | --- | --- |
| 1:1 | 0.76 | 0.43 |
| 1:2 | 0.79 | 0.41 |

*Phase 2 Accuracy per block*

| Block | Mean accuracy | SD accuracy |
| --- | --- | --- |
| 1 | 0.58 | 0.49 |
| 2 | 0.86 | 0.35 |
| 3 | 0.91 | 0.29 |
| 4 | 0.93 | 0.25 |
| 5 | 0.95 | 0.22 |

*Phase 2 Accuracy per mapping type and block*

| Mapping | 1:1 | | 1:2 | |
| --- | --- | --- | --- | --- |
| Block | Mean accuracy | SD accuracy | Mean accuracy | SD accuracy |
| 1 | 0.51 | 0.50 | 0.53 | 0.50 |
| 2 | 0.73 | 0.45 | 0.77 | 0.42 |
| 3 | 0.82 | 0.39 | 0.83 | 0.38 |
| 4 | 0.87 | 0.33 | 0.90 | 0.31 |
| 5 | 0.90 | 0.30 | 0.91 | 0.28 |

*Phase 1 RTs per mapping type*

| Mapping type | Mean accuracy (ms) | SD accuracy (ms) |
| --- | --- | --- |
| 1:1 | 1960 | 1267 |
| 1:2 | 2697 | 1653 |

*Phase 1 RTs per block*

| Block | Mean accuracy (ms) | SD accuracy (ms) |
| --- | --- | --- |
| 1 | 3303 | 1829 |
| 2 | 2481 | 1594 |
| 3 | 2041 | 1371 |
| 4 | 1904 | 1304 |
| 5 | 1774 | 1180 |

*Phase 1 RTs per mapping type and block*

| Mapping | 1:1 | | 1:2 | |
| --- | --- | --- | --- | --- |
| Block | Mean accuracy (ms) | SD accuracy (ms) | Mean accuracy (ms) | SD accuracy (ms) |
| 1 | 3179 | 1827 | 3605 | 1801 |
| 2 | 2238 | 1461 | 3114 | 1744 |
| 3 | 1784 | 1156 | 2689 | 1632 |
| 4 | 1611 | 1001 | 2597 | 1636 |
| 5 | 1512 | 917 | 2386 | 1464 |

*Phase 2 RTs per mapping type*

| Mapping type | Mean accuracy (ms) | SD accuracy (ms) |
| --- | --- | --- |
| 1:1 | 1676 | 1106 |
| 1:2 | 1715 | 1138 |

*Phase 2 RTs per block*

| Block | Mean accuracy (ms) | SD accuracy (ms) |
| --- | --- | --- |
| 1 | 2661 | 1573 |
| 2 | 1914 | 1237 |
| 3 | 1531 | 906 |
| 4 | 1410 | 748 |
| 5 | 1336 | 725 |

*Phase 2 RTs per mapping type and block*

| Mapping | 1:1 | | 1:2 | |
| --- | --- | --- | --- | --- |
| Block | Mean accuracy (ms) | SD accuracy (ms) | Mean accuracy (ms) | SD accuracy (ms) |
| 1 | 2626 | 2626 | 2733 | 1607 |
| 2 | 1898 | 1231 | 1948 | 1249 |
| 3 | 1505 | 865 | 1587 | 983 |
| 4 | 1411 | 758 | 1408 | 728 |
| 5 | 1339 | 750 | 1330 | 670 |

**GRAPHS:**

**RTs - LP1:**

**
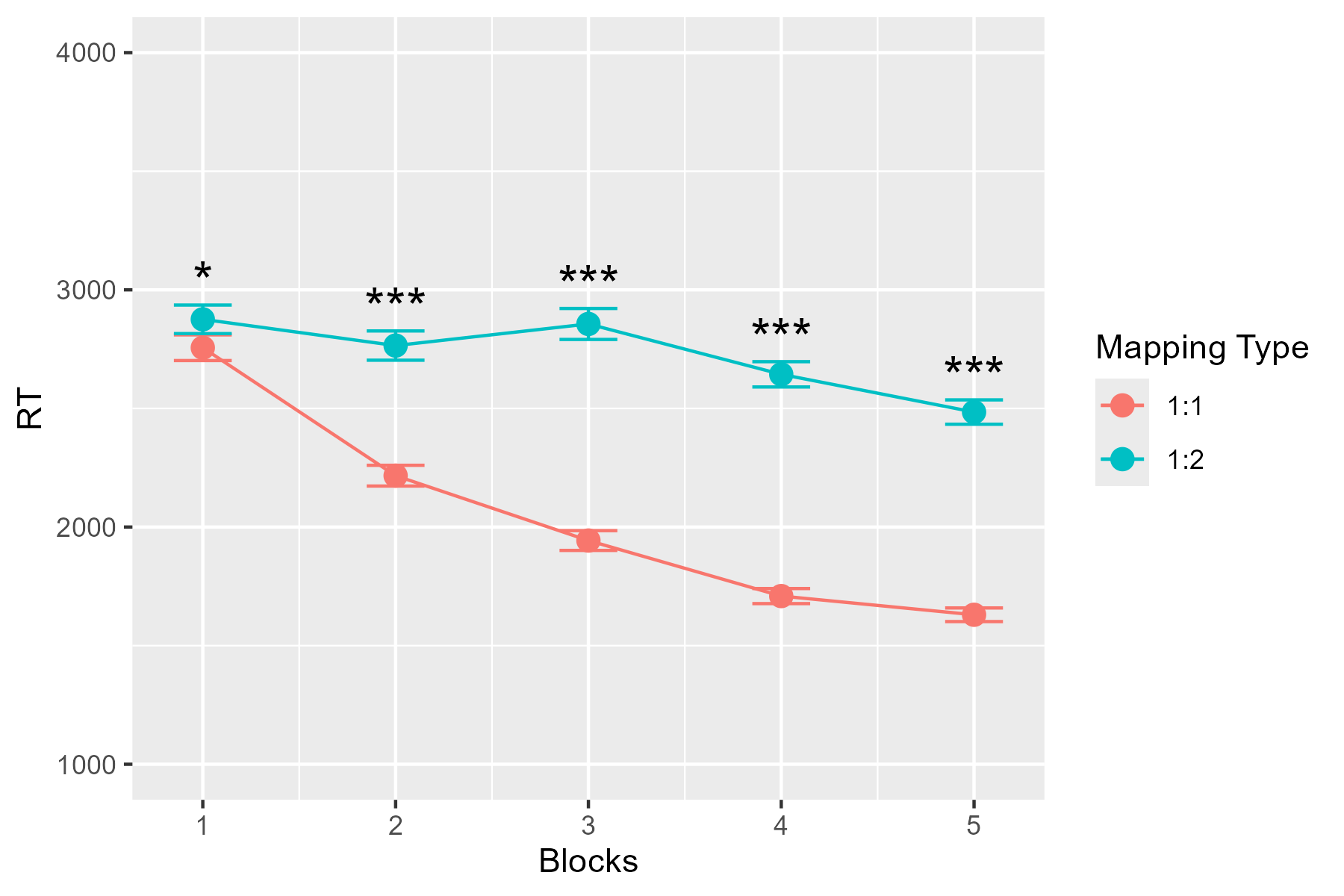
**

**RTs - LP2:**


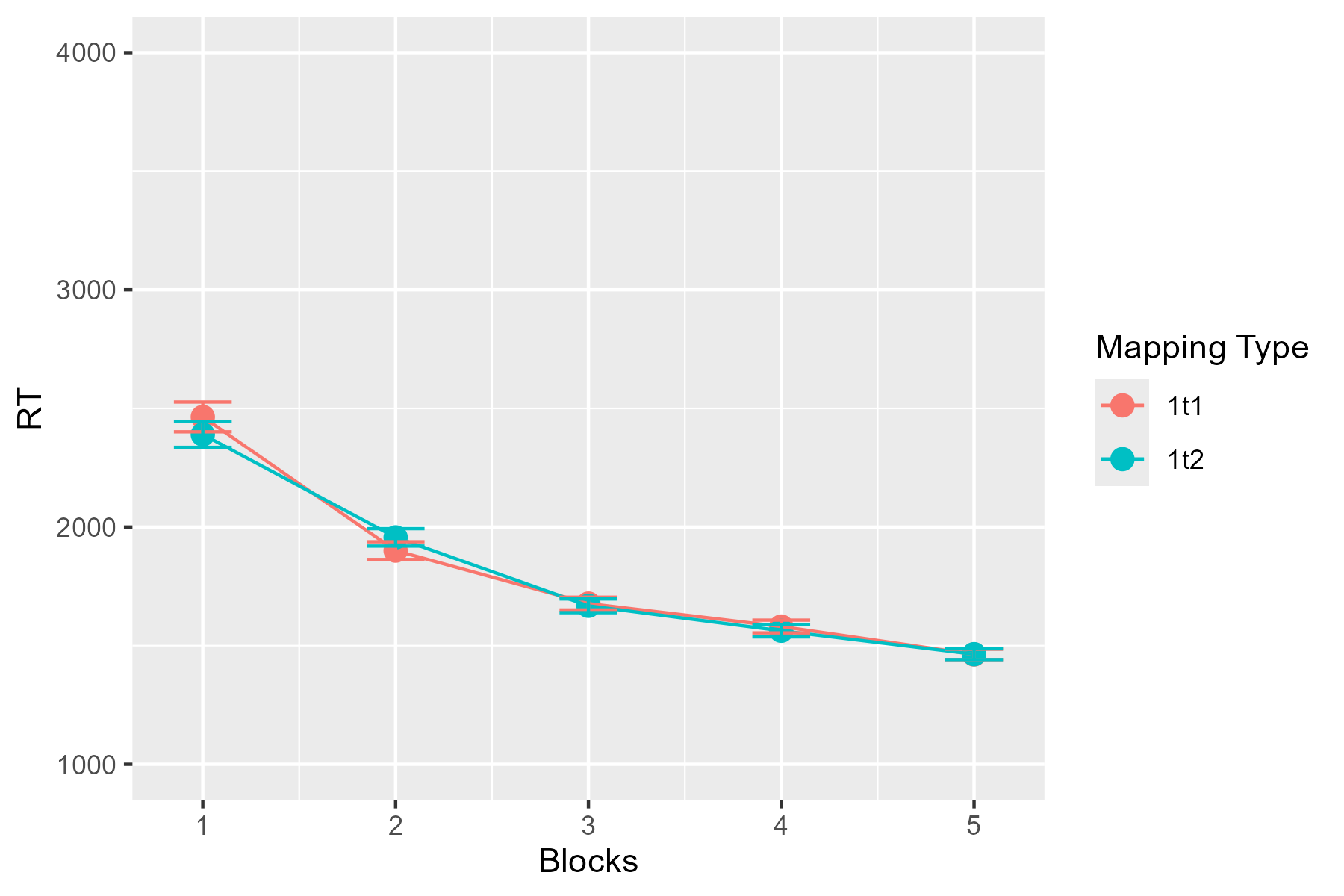


**Between 2 and 2 Analyses**

**Experiment 1 vs. Experiment 2**

**H1**

**Full results:**

Formula: accuracy ~ 1 + mapping_type_cc + block_s + mapping_type_cc:block_s +

exp_cc + mapping_type_cc:exp_cc + block_s:exp_cc + mapping_type_cc:block_s:exp_cc +

(1 + mapping_type_cc | subject) + (1 + mapping_type_cc + block_s | target) +

(1 + mapping_type_cc | word)

Data: phase1

| AIC | BIC | logLik | deviance | df.resid |
| --- | --- | --- | --- | --- |
| 28164.8 | 28331.5 | -14062.4 | 28124.8 | 30700 |

Scaled residuals:

| Min | 1Q | Median | 3Q | Max |
| --- | --- | --- | --- | --- |
| -11.3203 | -0.4039 | 0.3 | 0.5345 | 4.3529 |

Random effects:

| Groups | Name | Variance | Std.Dev. | Corr |
| --- | --- | --- | --- | --- |
| subject | (Intercept) | 0.565296 | 0.75186 |  |
|  | mapping_type_cc | 0.342077 | 0.58487 | 0.48 |
| target | (Intercept) | 0.038459 | 0.19611 |  |
|  | mapping_type_cc | 0.077603 | 0.27857 | 0.27 |
|  | block_s | 0.005124 | 0.07158 | -0.62 |
| word | (Intercept) | 0.017766 | 0.13329 |  |
|  | mapping_type_cc | 0.065408 | 0.25575 | 0.14 |

Number of obs: 30720, groups: subject, 128; target, 28; word, 12

Fixed effects:

|  | Estimate | Std. Error | z value | Pr(>\|z\|) |  |
| --- | --- | --- | --- | --- | --- |
| (Intercept) | 1.310448 | 0.087404 | 14.993 | < 2e-16 | *** |
| mapping_type_cc | 0.970881 | 0.111135 | 8.736 | < 2e-16 | *** |
| block_s | 0.654832 | 0.018338 | 35.709 | < 2e-16 | *** |
| exp_cc | -0.00996 | 0.138028 | -0.072 | 0.942455 |  |
| mapping_type_cc:block_s | 0.23325 | 0.024698 | 9.444 | < 2e-16 | *** |
| mapping_type_cc:exp_cc | 0.147059 | 0.127062 | 1.157 | 0.247117 |  |
| block_s:exp_cc | 0.01392 | 0.024086 | 0.578 | 0.563311 |  |
| mapping_type_cc:block_s:exp_cc | 0.184494 | 0.048574 | 3.798 | 0.000146 | *** |

---

Signif. codes: 0 ‘***’ 0.001 ‘**’ 0.01 ‘*’ 0.05 ‘.’ 0.1 ‘ ’ 1

Correlation of Fixed Effects:

|  | (Intr) | mppn__ | blck_s | exp_cc | mppng_typ_cc:b_ | mppng_typ_cc:x_ | blc_:_ |
| --- | --- | --- | --- | --- | --- | --- | --- |
| mppng_typ_c | 0.261 |  |  |  |  |  |  |
| block_s | -0.037 | -0.084 |  |  |  |  |  |
| exp_cc | -0.033 | 0.001 | -0.003 |  |  |  |  |
| mppng_typ_cc:b_ | 0.029 | 0.123 | -0.023 | 0.008 |  |  |  |
| mppng_typ_cc:x_ | 0.002 | -0.046 | 0.011 | 0.364 | -0.011 |  |  |
| blck_s:xp_c | -0.002 | 0.008 | -0.042 | 0.089 | 0.036 | 0.07 |  |
| mppng__:_:_ | 0.006 | -0.005 | 0.03 | 0.031 | -0.068 | 0.189 | -0.058 |

**Post – hoc (Mapping type * Block * Experiment)**

**Split for Block:**

**Block 1:**

Generalized linear mixed model fit by maximum likelihood (Laplace Approximation) ['glmerMod']

Family: binomial ( logit )

Formula: accuracy ~ 1 + mapping_type_cc + exp_cc + mapping_type_cc:exp_cc +

(1 + mapping_type_cc | subject) + (1 | target) + (1 + mapping_type_cc | word)

Data: get(lp_var)

| AIC | BIC | logLik | deviance | df.resid |
| --- | --- | --- | --- | --- |
| 8335.8 | 8409.7 | -4156.9 | 8313.8 | 6133 |

Scaled residuals:

| Min | 1Q | Median | 3Q | Max |
| --- | --- | --- | --- | --- |
| -1.6833 | -0.9011 | -0.6518 | 0.9827 | 1.8023 |

Random effects:

| Groups | Name | Variance | Std.Dev. | Corr |
| --- | --- | --- | --- | --- |
| subject | (Intercept) | 0.092122 | 0.30352 |  |
|  | mapping_type_cc | 0.154398 | 0.39293 | 0.54 |
| target | (Intercept) | 0.052172 | 0.22841 |  |
| word | (Intercept) | 0.009089 | 0.09534 |  |
|  | mapping_type_cc | 0.133439 | 0.36529 | -0.62 |

Number of obs: 6144, groups: subject, 128; target, 28; word, 12

Fixed effects:

|  | Estimate | Std. Error | z value | Pr(>\|z\|) |  |
| --- | --- | --- | --- | --- | --- |
| (Intercept) | -0.20782 | 0.06526 | -3.185 | 0.00145 | ** |
| mapping_type_cc | 0.46378 | 0.12596 | 3.682 | 0.000231 | *** |
| exp_cc | 0.01428 | 0.07987 | 0.179 | 0.858102 |  |
| mapping_type_cc:exp_cc | -0.29125 | 0.13793 | -2.112 | 0.034722 | * |

---

Signif. codes: 0 ‘***’ 0.001 ‘**’ 0.01 ‘*’ 0.05 ‘.’ 0.1 ‘ ’ 1

Correlation of Fixed Effects:

|  | (Intr) | mppn__ | exp_cc |
| --- | --- | --- | --- |
| mppng_typ_c | -0.24 |  |  |
| exp_cc | -0.055 | 0.021 |  |
| mppng_ty_:_ | 0.026 | -0.054 | -0.051 |

**Block 2:**

Generalized linear mixed model fit by maximum likelihood (Laplace Approximation) ['glmerMod']

Family: binomial ( logit )

Formula: accuracy ~ 1 + mapping_type_cc + exp_cc + mapping_type_cc:exp_cc +

(1 + mapping_type_cc | subject) + (1 + mapping_type_cc | target)

Data: get(lp_var)

| AIC | BIC | logLik | deviance | df.resid |
| --- | --- | --- | --- | --- |
| 6819.3 | 6886.6 | -3399.7 | 6799.3 | 6134 |

Scaled residuals:

| Min | 1Q | Median | 3Q | Max |
| --- | --- | --- | --- | --- |
| -5.3943 | -0.9105 | 0.3993 | 0.6655 | 2.0637 |

Random effects:

| Groups | Name | Variance | Std.Dev. | Corr |
| --- | --- | --- | --- | --- |
| subject | (Intercept) | 0.61763 | 0.7859 |  |
|  | mapping_type_cc | 0.43536 | 0.6598 | 0.9 |
| target | (Intercept) | 0.04262 | 0.2064 |  |
|  | mapping_type_cc | 0.09125 | 0.3021 | 0.66 |

Number of obs: 6144, groups: subject, 128; target, 28

Fixed effects:

|  | Estimate | Std. Error | z value | Pr(>\|z\|) |  |
| --- | --- | --- | --- | --- | --- |
| (Intercept) | 0.91756 | 0.08686 | 10.564 | <2e-16 | *** |
| mapping_type_cc | 0.99191 | 0.10625 | 9.336 | <2e-16 | *** |
| exp_cc | -0.09815 | 0.1539 | -0.638 | 0.524 |  |
| mapping_type_cc:exp_cc | -0.10676 | 0.17491 | -0.61 | 0.542 |  |

---

Signif. codes: 0 ‘***’ 0.001 ‘**’ 0.01 ‘*’ 0.05 ‘.’ 0.1 ‘ ’ 1

Correlation of Fixed Effects:

|  | (Intr) | mppn__ | exp_cc |
| --- | --- | --- | --- |
| mppng_typ_c | 0.56 |  |  |
| exp_cc | -0.044 | -0.026 |  |
| mppng_ty_:_ | -0.027 | -0.066 | 0.523 |

**Block 3:**

Generalized linear mixed model fit by maximum likelihood (Laplace Approximation) ['glmerMod']

Family: binomial ( logit )

Formula: accuracy ~ 1 + mapping_type_cc + exp_cc + mapping_type_cc:exp_cc +

(1 | subject) + (1 + mapping_type_cc | word) + (1 + mapping_type_cc | target)

Data: get(lp_var)

| AIC | BIC | logLik | deviance | df.resid |
| --- | --- | --- | --- | --- |
| 5325.4 | 5399.4 | -2651.7 | 5303.4 | 6133 |

Scaled residuals:

| Min | 1Q | Median | 3Q | Max |
| --- | --- | --- | --- | --- |
| -5.9453 | 0.1788 | 0.3178 | 0.4691 | 2.662 |

Random effects:

| Groups | Name | Variance | Std.Dev. | Corr |
| --- | --- | --- | --- | --- |
| subject | (Intercept) | 1.09073 | 1.0444 |  |
| target | (Intercept) | 0.03526 | 0.1878 |  |
|  | mapping_type_cc | 0.27054 | 0.5201 | -0.54 |
| word | (Intercept) | 0.03195 | 0.1787 |  |
|  | mapping_type_cc | 0.02108 | 0.1452 | 1 |

Number of obs: 6144, groups: subject, 128; target, 28; word, 12

Fixed effects:

|  | Estimate | Std. Error | z value | Pr(>\|z\|) |  |
| --- | --- | --- | --- | --- | --- |
| (Intercept) | 1.62607 | 0.12003 | 13.547 | <2e-16 | *** |
| mapping_type_cc | 1.08731 | 0.13215 | 8.228 | <2e-16 | *** |
| exp_cc | -0.04926 | 0.20216 | -0.244 | 0.807 |  |
| mapping_type_cc:exp_cc | 0.15976 | 0.15127 | 1.056 | 0.291 |  |

---

Signif. codes: 0 ‘***’ 0.001 ‘**’ 0.01 ‘*’ 0.05 ‘.’ 0.1 ‘ ’ 1

Correlation of Fixed Effects:

|  | (Intr) | mppn__ | exp_cc |
| --- | --- | --- | --- |
| mppng_typ_c | 0.014 |  |  |
| exp_cc | -0.037 | 0.009 |  |
| mppng_ty_:_ | 0.013 | -0.07 | -0.039 |

**Block 4:**

Generalized linear mixed model fit by maximum likelihood (Laplace Approximation) ['glmerMod']

Family: binomial ( logit )

Formula: accuracy ~ 1 + mapping_type_cc + exp_cc + mapping_type_cc:exp_cc +

(1 + mapping_type_cc | subject) + (1 | target)

Data: get(lp_var)

| AIC | BIC | logLik | deviance | df.resid |
| --- | --- | --- | --- | --- |
| 3993.5 | 4047.3 | -1988.8 | 3977.5 | 6136 |

Scaled residuals:

| Min | 1Q | Median | 3Q | Max |
| --- | --- | --- | --- | --- |
| -6.9454 | 0.0908 | 0.2171 | 0.367 | 1.7573 |

Random effects:

| Groups | Name | Variance | Std.Dev. | Corr |
| --- | --- | --- | --- | --- |
| subject | (Intercept) | 2.06963 | 1.4386 |  |
|  | mapping_type_cc | 1.00303 | 1.0015 | 0.73 |
| target | (Intercept) | 0.03824 | 0.1955 |  |

Number of obs: 6144, groups: subject, 128; target, 28

Fixed effects:

|  | Estimate | Std. Error | z value | Pr(>\|z\|) |  |
| --- | --- | --- | --- | --- | --- |
| (Intercept) | 2.4481 | 0.1491 | 16.422 | <2e-16 | *** |
| mapping_type_cc | 1.6789 | 0.1576 | 10.651 | <2e-16 | *** |
| exp_cc | 0.1341 | 0.2784 | 0.482 | 0.63 |  |
| mapping_type_cc:exp_cc | 0.3431 | 0.2673 | 1.284 | 0.199 |  |

---

Signif. codes: 0 ‘***’ 0.001 ‘**’ 0.01 ‘*’ 0.05 ‘.’ 0.1 ‘ ’ 1

Correlation of Fixed Effects:

|  | (Intr) | mppn__ | exp_cc |
| --- | --- | --- | --- |
| mppng_typ_c | 0.518 |  |  |
| exp_cc | -0.013 | 0.021 |  |
| mppng_ty_:_ | 0.021 | 0.02 | 0.532 |

**Block 5:**

Generalized linear mixed model fit by maximum likelihood (Laplace Approximation) ['glmerMod']

Family: binomial ( logit )

Formula: accuracy ~ 1 + mapping_type_cc + exp_cc + mapping_type_cc:exp_cc +

(1 + mapping_type_cc | subject) + (1 | word)

Data: get(lp_var)

| AIC | BIC | logLik | deviance | df.resid |
| --- | --- | --- | --- | --- |
| 3387.2 | 3441 | -1685.6 | 3371.2 | 6136 |

Scaled residuals:

| Min | 1Q | Median | 3Q | Max |
| --- | --- | --- | --- | --- |
| -10.2848 | 0.0738 | 0.1771 | 0.3094 | 1.9077 |

Random effects:

| Groups | Name | Variance | Std.Dev. | Corr |
| --- | --- | --- | --- | --- |
| subject | (Intercept) | 2.62575 | 1.6204 |  |
|  | mapping_type_cc | 0.61035 | 0.7812 | 1 |
| word | (Intercept) | 0.09254 | 0.3042 |  |

Number of obs: 6144, groups: subject, 128; word, 12

Fixed effects:

|  | Estimate | Std. Error | z value | Pr(>\|z\|) |  |
| --- | --- | --- | --- | --- | --- |
| (Intercept) | 2.89174 | 0.18815 | 15.369 | <2e-16 | *** |
| mapping_type_cc | 1.86071 | 0.16861 | 11.035 | <2e-16 | *** |
| exp_cc | -0.05963 | 0.3167 | -0.188 | 0.8507 |  |
| mapping_type_cc:exp_cc | 0.44834 | 0.25236 | 1.777 | 0.0756 | . |

---

Signif. codes: 0 ‘***’ 0.001 ‘**’ 0.01 ‘*’ 0.05 ‘.’ 0.1 ‘ ’ 1

Correlation of Fixed Effects:

|  | (Intr) | mppn__ | exp_cc |
| --- | --- | --- | --- |
| mppng_typ_c | 0.522 |  |  |
| exp_cc | -0.024 | 0.02 |  |
| mppng_ty_:_ | 0.009 | -0.015 | 0.622 |

**Split for Experiment:**

**Experiment 1:**

Generalized linear mixed model fit by maximum likelihood (Laplace Approximation) ['glmerMod']

Family: binomial ( logit )

Formula: accuracy ~ 1 + mapping_type_cc + block_s + mapping_type_cc:block_s +

(1 + block_s + mapping_type_cc | subject) + (1 | target)

Data: get(lp_var)

| AIC | BIC | logLik | deviance | df.resid |
| --- | --- | --- | --- | --- |
| 14145.7 | 14230.1 | -7061.8 | 14123.7 | 15829 |

Scaled residuals:

| Min | 1Q | Median | 3Q | Max |
| --- | --- | --- | --- | --- |
| -15.7488 | -0.6268 | 0.2412 | 0.5554 | 1.9444 |

Random effects:

| Groups | Name | Variance | Std.Dev. | Corr |  |
| --- | --- | --- | --- | --- | --- |
| subject | (Intercept) | 0.99807 | 0.999 |  |  |
|  | block_s | 0.18666 | 0.432 | 0.92 |  |
|  | mapping_type_cc | 0.48213 | 0.6944 | 0.5 | 0.38 |
| target | (Intercept) | 0.04164 | 0.2041 |  |  |

Number of obs: 15840, groups: subject, 66; target, 28

Fixed effects:

|  | Estimate | Std. Error | z value | Pr(>\|z\|) |  |
| --- | --- | --- | --- | --- | --- |
| (Intercept) | 1.53936 | 0.13287 | 11.59 | <2e-16 | *** |
| mapping_type_cc | 1.16355 | 0.10075 | 11.55 | <2e-16 | *** |
| block_s | 0.82302 | 0.0573 | 14.36 | <2e-16 | *** |
| mapping_type_cc:block_s | 0.40545 | 0.03528 | 11.49 | <2e-16 | *** |

---

Signif. codes: 0 ‘***’ 0.001 ‘**’ 0.01 ‘*’ 0.05 ‘.’ 0.1 ‘ ’ 1

Correlation of Fixed Effects:

|  | (Intr) | mppn__ | blck_s |
| --- | --- | --- | --- |
| mppng_typ_c | 0.413 |  |  |
| block_s | 0.852 | 0.343 |  |
| mppng_ty_:_ | 0.043 | 0.256 | 0.015 |

**Experiment 2:**

Generalized linear mixed model fit by maximum likelihood (Laplace Approximation) ['glmerMod']

Family: binomial ( logit )

Formula: accuracy ~ 1 + mapping_type_cc + block_s + mapping_type_cc:block_s +

(1 + block_s + mapping_type_cc | subject) + (1 | target)

Data: get(lp_var)

| AIC | BIC | logLik | deviance | df.resid |
| --- | --- | --- | --- | --- |
| 13153.3 | 13237 | -6565.6 | 13131.3 | 14869 |

Scaled residuals:

| Min | 1Q | Median | 3Q | Max |
| --- | --- | --- | --- | --- |
| -42.109 | -0.545 | 0.245 | 0.555 | 2.07 |

Random effects:

| Groups | Name | Variance | Std.Dev. | Corr |  |
| --- | --- | --- | --- | --- | --- |
| subject | (Intercept) | 1.1304 | 1.0632 |  |  |
|  | block_s | 0.2048 | 0.4526 | 0.96 |  |
|  | mapping_type_cc | 0.2777 | 0.527 | 0.61 | 0.54 |
| target | (Intercept) | 0.0762 | 0.276 |  |  |

Number of obs: 14880, groups: subject, 62; target, 27

Fixed effects:

|  | Estimate | Std. Error | z value | Pr(>\|z\|) |  |
| --- | --- | --- | --- | --- | --- |
| (Intercept) | 1.56589 | 0.14936 | 10.484 | < 2e-16 | *** |
| mapping_type_cc | 1.06226 | 0.09037 | 11.754 | < 2e-16 | *** |
| block_s | 0.83044 | 0.06178 | 13.442 | < 2e-16 | *** |
| mapping_type_cc:block_s | 0.23414 | 0.03643 | 6.428 | 1.29E-10 | *** |

---

Signif. codes: 0 ‘***’ 0.001 ‘**’ 0.01 ‘*’ 0.05 ‘.’ 0.1 ‘ ’ 1

Correlation of Fixed Effects:

|  | (Intr) | mppn__ | blck_s |
| --- | --- | --- | --- |
| mppng_typ_c | 0.419 |  |  |
| block_s | 0.863 | 0.406 |  |
| mppng_ty_:_ | 0.029 | 0.297 | -0.007 |

**Split for Mapping:**

**1:1**

Generalized linear mixed model fit by maximum likelihood (Laplace Approximation) ['glmerMod']

Family: binomial ( logit )

Formula: accuracy ~ 1 + exp_cc + block_s + exp_cc:block_s + (1 + block_s | subject) + (1 | target)

Data: get(lp_var)

| AIC | BIC | logLik | deviance | df.resid |
| --- | --- | --- | --- | --- |
| 16027.7 | 16091.1 | -8005.8 | 16011.7 | 20472 |

Scaled residuals:

| Min | 1Q | Median | 3Q | Max |
| --- | --- | --- | --- | --- |
| -29.4946 | 0.033 | 0.2004 | 0.4869 | 2.0738 |

Random effects:

| Groups | Name | Variance | Std.Dev. | Corr |
| --- | --- | --- | --- | --- |
| subject | (Intercept) | 2.05952 | 1.4351 |  |
|  | block_s | 0.34064 | 0.5836 | 0.94 |
| target | (Intercept) | 0.09531 | 0.3087 |  |

Number of obs: 20480, groups: subject, 128; target, 28

Fixed effects:

|  | Estimate | Std. Error | z value | Pr(>\|z\|) |  |
| --- | --- | --- | --- | --- | --- |
| (Intercept) | 2.31563 | 0.14669 | 15.786 | <2e-16 | *** |
| exp_cc | 0.03136 | 0.26545 | 0.118 | 0.906 |  |
| block_s | 1.09563 | 0.05807 | 18.869 | <2e-16 | *** |
| exp_cc:block_s | 0.1072 | 0.11329 | 0.946 | 0.344 |  |

---

Signif. codes: 0 ‘***’ 0.001 ‘**’ 0.01 ‘*’ 0.05 ‘.’ 0.1 ‘ ’ 1

Correlation of Fixed Effects:

|  | (Intr) | exp_cc | blck_s |
| --- | --- | --- | --- |
| exp_cc | -0.031 |  |  |
| block_s | 0.838 | -0.027 |  |
| exp_cc:blc_ | -0.025 | 0.912 | -0.029 |

**1:2**

Generalized linear mixed model fit by maximum likelihood (Laplace Approximation) ['glmerMod']

Family: binomial ( logit )

Formula: accuracy ~ 1 + exp_cc + block_s + exp_cc:block_s + (1 + block_s | subject) +

(1 + block_s | target) + (1 | word)

Data: get(lp_var)

| AIC | BIC | logLik | deviance | df.resid |
| --- | --- | --- | --- | --- |
| 11274.9 | 11354.5 | -5626.5 | 11252.9 | 10229 |

Scaled residuals:

| Min | 1Q | Median | 3Q | Max |
| --- | --- | --- | --- | --- |
| -7.0387 | -0.8311 | 0.3301 | 0.6975 | 1.7431 |

Random effects:

| Groups | Name | Variance | Std.Dev. | Corr |
| --- | --- | --- | --- | --- |
| subject | (Intercept) | 0.726261 | 0.85221 |  |
|  | block_s | 0.124173 | 0.35238 | 0.95 |
| target | (Intercept) | 0.054412 | 0.23326 |  |
|  | block_s | 0.005833 | 0.07638 | 0.74 |
| word | (Intercept) | 0.027792 | 0.16671 |  |

Number of obs: 10240, groups: subject, 128; target, 28; word, 12

Fixed effects:

|  | Estimate | Std. Error | z value | Pr(>\|z\|) |  |
| --- | --- | --- | --- | --- | --- |
| (Intercept) | 0.98072 | 0.10473 | 9.364 | <2e-16 | *** |
| exp_cc | -0.11374 | 0.16233 | -0.701 | 0.484 |  |
| block_s | 0.64059 | 0.04021 | 15.93 | <2e-16 | *** |
| exp_cc:block_s | -0.08845 | 0.07365 | -1.201 | 0.23 |  |

---

Signif. codes: 0 ‘***’ 0.001 ‘**’ 0.01 ‘*’ 0.05 ‘.’ 0.1 ‘ ’ 1

Correlation of Fixed Effects:

|  | (Intr) | exp_cc | blck_s |
| --- | --- | --- | --- |
| exp_cc | -0.046 |  |  |
| block_s | 0.715 | -0.037 |  |
| exp_cc:blc_ | -0.031 | 0.825 | -0.055 |

**Post – hoc (Mapping type * Block)**

**Split for Block:**

**Block 1:**

Generalized linear mixed model fit by maximum likelihood (Laplace Approximation) ['glmerMod']

Family: binomial ( logit )

Formula: accuracy ~ 1 + mapping_type_cc + (1 + mapping_type_cc | subject) +

(1 | target) + (1 + mapping_type_cc | word)

Data: get(lp_var)

| AIC | BIC | logLik | deviance | df.resid |
| --- | --- | --- | --- | --- |
| 8336.2 | 8396.7 | -4159.1 | 8318.2 | 6135 |

Scaled residuals:

| Min | 1Q | Median | 3Q | Max |
| --- | --- | --- | --- | --- |
| -1.7102 | -0.9008 | -0.6598 | 0.9801 | 1.8484 |

Random effects:

| Groups | Name | Variance | Std.Dev. | Corr |
| --- | --- | --- | --- | --- |
| subject | (Intercept) | 0.092255 | 0.30374 |  |
|  | mapping_type_cc | 0.172368 | 0.41517 | 0.5 |
| target | (Intercept) | 0.052625 | 0.2294 |  |
| word | (Intercept) | 0.008852 | 0.09409 |  |
|  | mapping_type_cc | 0.134877 | 0.36726 | -0.62 |

Number of obs: 6144, groups: subject, 128; target, 28; word, 12

Fixed effects:

|  | Estimate | Std. Error | z value | Pr(>\|z\|) |  |
| --- | --- | --- | --- | --- | --- |
| (Intercept) | -0.20479 | 0.06513 | -3.144 | 0.001665 | ** |
| mapping_type_cc | 0.45071 | 0.12679 | 3.555 | 0.000378 | *** |

---

Signif. codes: 0 ‘***’ 0.001 ‘**’ 0.01 ‘*’ 0.05 ‘.’ 0.1 ‘ ’ 1

Correlation of Fixed Effects:

(Intr)

mppng_typ_c -0.233

**Block 2:**

Generalized linear mixed model fit by maximum likelihood (Laplace Approximation) ['glmerMod']

Family: binomial ( logit )

Formula: accuracy ~ 1 + mapping_type_cc + (1 + mapping_type_cc | subject) +

(1 + mapping_type_cc | target)

Data: get(lp_var)

| AIC | BIC | logLik | deviance | df.resid |
| --- | --- | --- | --- | --- |
| 6815.8 | 6869.6 | -3399.9 | 6799.8 | 6136 |

Scaled residuals:

| Min | 1Q | Median | 3Q | Max |
| --- | --- | --- | --- | --- |
| -5.4507 | -0.9104 | 0.4001 | 0.6668 | 2.0544 |

Random effects:

| Groups | Name | Variance | Std.Dev. | Corr |
| --- | --- | --- | --- | --- |
| subject | (Intercept) | 0.61907 | 0.7868 |  |
|  | mapping_type_cc | 0.43727 | 0.6613 | 0.9 |
| target | (Intercept) | 0.04261 | 0.2064 |  |
|  | mapping_type_cc | 0.08958 | 0.2993 | 0.66 |

Number of obs: 6144, groups: subject, 128; target, 28

Fixed effects:

|  | Estimate | Std. Error | z value | Pr(>\|z\|) |  |
| --- | --- | --- | --- | --- | --- |
| (Intercept) | 0.91527 | 0.08684 | 10.54 | <2e-16 | *** |
| mapping_type_cc | 0.9886 | 0.10582 | 9.342 | <2e-16 | *** |

---

Signif. codes: 0 ‘***’ 0.001 ‘**’ 0.01 ‘*’ 0.05 ‘.’ 0.1 ‘ ’ 1

Correlation of Fixed Effects:

(Intr)

mppng_typ_c 0.561

**Block 3:**

Generalized linear mixed model fit by maximum likelihood (Laplace Approximation) ['glmerMod']

Family: binomial ( logit )

Formula: accuracy ~ 1 + mapping_type_cc + (1 | subject) + (1 | word) +

(1 + mapping_type_cc | target)

Data: get(lp_var)

| AIC | BIC | logLik | deviance | df.resid |
| --- | --- | --- | --- | --- |
| 5321.1 | 5368.1 | -2653.5 | 5307.1 | 6137 |

Scaled residuals:

| Min | 1Q | Median | 3Q | Max |
| --- | --- | --- | --- | --- |
| -5.721 | 0.1761 | 0.3185 | 0.4634 | 2.7775 |

Random effects:

| Groups | Name | Variance | Std.Dev. | Corr |
| --- | --- | --- | --- | --- |
| subject | (Intercept) | 1.09797 | 1.0478 |  |
| target | (Intercept) | 0.03759 | 0.1939 |  |
|  | mapping_type_cc | 0.26952 | 0.5192 | -0.56 |
| word | (Intercept) | 0.03613 | 0.1901 |  |

Number of obs: 6144, groups: subject, 128; target, 28; word, 12

Fixed effects:

|  | Estimate | Std. Error | z value | Pr(>\|z\|) |  |
| --- | --- | --- | --- | --- | --- |
| (Intercept) | 1.6208 | 0.122 | 13.29 | <2e-16 | *** |
| mapping_type_cc | 1.0889 | 0.1251 | 8.705 | <2e-16 | *** |

---

Signif. codes: 0 ‘***’ 0.001 ‘**’ 0.01 ‘*’ 0.05 ‘.’ 0.1 ‘ ’ 1

Correlation of Fixed Effects:

(Intr)

mppng_typ_c -0.137

**Block 4:**Generalized linear mixed model fit by maximum likelihood (Laplace Approximation) ['glmerMod']

Family: binomial ( logit )

Formula: accuracy ~ 1 + mapping_type_cc + (1 + mapping_type_cc | subject) + (1 | word) +

(1 | target)

Data: get(lp_var)

| AIC | BIC | logLik | deviance | df.resid |
| --- | --- | --- | --- | --- |
| 3964.9 | 4012 | -1975.5 | 3950.9 | 6137 |

Scaled residuals:

| Min | 1Q | Median | 3Q | Max |
| --- | --- | --- | --- | --- |
| -7.1564 | 0.0926 | 0.2104 | 0.3642 | 1.7976 |

Random effects:

| Groups | Name | Variance | Std.Dev. | Corr |
| --- | --- | --- | --- | --- |
| subject | (Intercept) | 2.12094 | 1.4563 |  |
|  | mapping_type_cc | 0.99358 | 0.9968 | 0.73 |
| target | (Intercept) | 0.03336 | 0.1827 |  |
| word | (Intercept) | 0.08482 | 0.2912 |  |

Number of obs: 6144, groups: subject, 128; target, 28; word, 12

Fixed effects:

|  | Estimate | Std. Error | z value | Pr(>\|z\|) |  |
| --- | --- | --- | --- | --- | --- |
| (Intercept) | 2.4794 | 0.172 | 14.42 | <2e-16 | *** |
| mapping_type_cc | 1.6701 | 0.1581 | 10.56 | <2e-16 | *** |

---

Signif. codes: 0 ‘***’ 0.001 ‘**’ 0.01 ‘*’ 0.05 ‘.’ 0.1 ‘ ’ 1

Correlation of Fixed Effects:

(Intr)

mppng_typ_c 0.444

**Block 5:**

Generalized linear mixed model fit by maximum likelihood (Laplace Approximation) ['glmerMod']

Family: binomial ( logit )

Formula: accuracy ~ 1 + mapping_type_cc + (1 | subject) + (1 | word) + (1 | target)

Data: get(lp_var)

| AIC | BIC | logLik | deviance | df.resid |
| --- | --- | --- | --- | --- |
| 3416 | 3449.6 | -1703 | 3406 | 6139 |

Scaled residuals:

| Min | 1Q | Median | 3Q | Max |
| --- | --- | --- | --- | --- |
| -10.006 | 0.1069 | 0.1871 | 0.2993 | 3.133 |

Random effects:

| Groups | Name | Variance | Std.Dev. |
| --- | --- | --- | --- |
| subject | (Intercept) | 2.4337 | 1.56 |
| target | (Intercept) | 0.05123 | 0.2263 |
| word | (Intercept) | 0.09044 | 0.3007 |

Number of obs: 6144, groups: subject, 128; target, 28; word, 12

Fixed effects:

|  | Estimate | Std. Error | z value | Pr(>\|z\|) |  |
| --- | --- | --- | --- | --- | --- |
| (Intercept) | 2.75408 | 0.18339 | 15.02 | <2e-16 | *** |
| mapping_type_cc | 1.24641 | 0.09767 | 12.76 | <2e-16 | *** |

---

Signif. codes: 0 ‘***’ 0.001 ‘**’ 0.01 ‘*’ 0.05 ‘.’ 0.1 ‘ ’ 1

Correlation of Fixed Effects:

(Intr)

mppng_typ_c 0.035

**H2a:**

Formula: accuracy ~ 1 + mapping_type_cc + block_s + mapping_type_cc:block_s +

exp_cc + mapping_type_cc:exp_cc + block_s:exp_cc + mapping_type_cc:block_s:exp_cc +

(1 + mapping_type_cc | subject) + (1 + mapping_type_cc | word) + (1 + block_s | target)

Data: phase2

| AIC | BIC | logLik | deviance | df.resid |
| --- | --- | --- | --- | --- |
| 22360.8 | 22502.5 | -11163.4 | 22326.8 | 30703 |

Scaled residuals:

| Min | 1Q | Median | 3Q | Max |
| --- | --- | --- | --- | --- |
| -12.2005 | 0.1072 | 0.2266 | 0.4114 | 4.9843 |

Random effects:

| Groups | Name | Variance | Std.Dev. | Corr |
| --- | --- | --- | --- | --- |
| subject | (Intercept) | 1.308667 | 1.144 |  |
|  | mapping_type_cc | 0.207987 | 0.4561 | -0.17 |
| target | (Intercept) | 0.014 | 0.1183 |  |
|  | block_s | 0.008409 | 0.0917 | 0.41 |
| word | (Intercept) | 0.023889 | 0.1546 |  |
|  | mapping_type_cc | 0.041806 | 0.2045 | -0.1 |

Number of obs: 30720, groups: subject, 128; target, 28; word, 12

Fixed effects:

|  | Estimate | Std. Error | z value | Pr(>\|z\|) |  |
| --- | --- | --- | --- | --- | --- |
| (Intercept) | 2.030425 | 0.115636 | 17.559 | < 2e-16 | *** |
| mapping_type_cc | 0.008668 | 0.087563 | 0.099 | 0.92115 |  |
| block_s | 0.819434 | 0.023707 | 34.565 | < 2e-16 | *** |
| exp_cc | 0.217896 | 0.208149 | 1.047 | 0.29518 |  |
| mapping_type_cc:block_s | 0.065694 | 0.032235 | 2.038 | 0.04155 | * |
| mapping_type_cc:exp_cc | 0.350839 | 0.123263 | 2.846 | 0.00442 | ** |
| block_s:exp_cc | -0.07375 | 0.031377 | -2.35 | 0.01875 | * |
| mapping_type_cc:block_s:exp_cc | 0.006495 | 0.061639 | 0.105 | 0.91608 |  |

---

Signif. codes: 0 ‘***’ 0.001 ‘**’ 0.01 ‘*’ 0.05 ‘.’ 0.1 ‘ ’ 1

Correlation of Fixed Effects:

|  | (Intr) | mppn__ | blck_s | exp_cc | mppng_typ_cc:b_ | mppng_typ_cc:x_ | blc_:_ |
| --- | --- | --- | --- | --- | --- | --- | --- |
| mppng_typ_c | -0.137 |  |  |  |  |  |  |
| block_s | 0.137 | -0.056 |  |  |  |  |  |
| exp_cc | -0.031 | 0.017 | -0.002 |  |  |  |  |
| mppng_typ_cc:b_ | -0.032 | 0.303 | -0.2 | 0.01 |  |  |  |
| mppng_typ_cc:x_ | 0.02 | -0.051 | 0.019 | -0.166 | -0.017 |  |  |
| blck_s:xp_c | -0.004 | 0.025 | -0.039 | 0.119 | 0.06 | -0.103 |  |
| mppng__:_:_ | 0.008 | -0.015 | 0.036 | -0.03 | -0.067 | 0.369 | -0.306 |

**Post – hoc (Mapping type * Experiment)**

**Split for Experiment:**

**Experiment 1:**

Generalized linear mixed model fit by maximum likelihood (Laplace Approximation) ['glmerMod']

Family: binomial ( logit )

Formula: accuracy ~ 1 + mapping_type_cc + (1 + mapping_type_cc | subject) +

(1 + mapping_type_cc | word)

Data: get(lp_var)

| AIC | BIC | logLik | deviance | df.resid |
| --- | --- | --- | --- | --- |
| 12888.1 | 12949.5 | -6436.1 | 12872.1 | 15832 |

Scaled residuals:

| Min | 1Q | Median | 3Q | Max |
| --- | --- | --- | --- | --- |
| -5.0864 | 0.2509 | 0.3284 | 0.4265 | 1.6748 |

Random effects:

| Groups | Name | Variance | Std.Dev. | Corr |
| --- | --- | --- | --- | --- |
| subject | (Intercept) | 0.86411 | 0.9296 |  |
|  | mapping_type_cc | 0.21727 | 0.4661 | -0.14 |
| word | (Intercept) | 0.01298 | 0.114 |  |
|  | mapping_type_cc | 0.07904 | 0.2811 | -0.61 |

Number of obs: 15840, groups: subject, 66; word, 12

Fixed effects:

|  | Estimate | Std. Error | z value | Pr(>\|z\|) |  |
| --- | --- | --- | --- | --- | --- |
| (Intercept) | 1.7692 | 0.12209 | 14.491 | <2e-16 | *** |
| mapping_type_cc | 0.09714 | 0.11303 | 0.859 | 0.39 |  |

---

Signif. codes: 0 ‘***’ 0.001 ‘**’ 0.01 ‘*’ 0.05 ‘.’ 0.1 ‘ ’ 1

Correlation of Fixed Effects:

(Intr)

mppng_typ_c -0.219

**Experiment 2:**

Generalized linear mixed model fit by maximum likelihood (Laplace Approximation) ['glmerMod']

Family: binomial ( logit )

Formula: accuracy ~ 1 + mapping_type_cc + (1 + mapping_type_cc | subject) +

(1 | target) + (1 + mapping_type_cc | word)

Data: get(lp_var)

| AIC | BIC | logLik | deviance | df.resid |
| --- | --- | --- | --- | --- |
| 13577.7 | 13646.2 | -6779.9 | 13559.7 | 14871 |

Scaled residuals:

| Min | 1Q | Median | 3Q | Max |
| --- | --- | --- | --- | --- |
| -4.8251 | 0.2417 | 0.3535 | 0.4906 | 1.8268 |

Random effects:

| Groups | Name | Variance | Std.Dev. | Corr |
| --- | --- | --- | --- | --- |
| subject | (Intercept) | 0.96214 | 0.9809 |  |
|  | mapping_type_cc | 0.05945 | 0.2438 | -0.45 |
| target | (Intercept) | 0.02756 | 0.166 |  |
| word | (Intercept) | 0.02051 | 0.1432 |  |
|  | mapping_type_cc | 0.04859 | 0.2204 | 0.76 |

Number of obs: 14880, groups: subject, 62; target, 26; word, 12

Fixed effects:

|  | Estimate | Std. Error | z value | Pr(>\|z\|) |  |
| --- | --- | --- | --- | --- | --- |
| (Intercept) | 1.53191 | 0.13855 | 11.057 | <2e-16 | *** |
| mapping_type_cc | -0.1976 | 0.09389 | -2.105 | 0.0353 | * |

---

Signif. codes: 0 ‘***’ 0.001 ‘**’ 0.01 ‘*’ 0.05 ‘.’ 0.1 ‘ ’ 1

Correlation of Fixed Effects:

(Intr)

mppng_typ_c -0.041

**Post – hoc (Block * Experiment)**

**Split for Block:**

**Block 1:**

Generalized linear mixed model fit by maximum likelihood (Laplace Approximation) ['glmerMod']

Family: binomial ( logit )

Formula: accuracy ~ 1 + exp_cc + (1 | subject) + (1 | target)

Data: get(lp_var)

| AIC | BIC | logLik | deviance | df.resid |
| --- | --- | --- | --- | --- |
| 8262.9 | 8289.8 | -4127.4 | 8254.9 | 6140 |

Scaled residuals:

| Min | 1Q | Median | 3Q | Max |
| --- | --- | --- | --- | --- |
| -1.9454 | -0.9459 | 0.579 | 0.8872 | 1.7666 |

Random effects:

| Groups | Name | Variance | Std.Dev. |  |
| --- | --- | --- | --- | --- |
|  | subject | (Intercept) | 0.27884 | 0.5281 |
|  | target | (Intercept) | 0.02835 | 0.1684 |

Number of obs: 6144, groups: subject, 128; target, 28

Fixed effects:

|  | Estimate | Std. Error | z value | Pr(>\|z\|) |  |
| --- | --- | --- | --- | --- | --- |
| (Intercept) | 0.11163 | 0.06284 | 1.776 | 0.07567 | . |
| exp_cc | 0.31039 | 0.10794 | 2.875 | 0.00403 | ** |

---

Signif. codes: 0 ‘***’ 0.001 ‘**’ 0.01 ‘*’ 0.05 ‘.’ 0.1 ‘ ’ 1

Correlation of Fixed Effects:

(Intr)

exp_cc -0.035

**Block 2:**

Generalized linear mixed model fit by maximum likelihood (Laplace Approximation) ['glmerMod']

Family: binomial ( logit )

Formula: accuracy ~ 1 + exp_cc + (1 | subject) + (1 | word)

Data: get(lp_var)

| AIC | BIC | logLik | deviance | df.resid |
| --- | --- | --- | --- | --- |
| 5111.1 | 5138 | -2551.6 | 5103.1 | 6140 |

Scaled residuals:

| Min | 1Q | Median | 3Q | Max |
| --- | --- | --- | --- | --- |
| -6.1705 | 0.1384 | 0.2555 | 0.4614 | 2.0338 |

Random effects:

| Groups | Name | Variance | Std.Dev. |
| --- | --- | --- | --- |
| subject | (Intercept) | 2.21388 | 1.488 |
| word | (Intercept) | 0.06658 | 0.258 |

Number of obs: 6144, groups: subject, 128; word, 12

Fixed effects:

|  | Estimate | Std. Error | z value | Pr(>\|z\|) |  |
| --- | --- | --- | --- | --- | --- |
| (Intercept) | 1.8732 | 0.1595 | 11.743 | <2e-16 | *** |
| exp_cc | 0.5679 | 0.2791 | 2.035 | 0.0419 | * |

---

Signif. codes: 0 ‘***’ 0.001 ‘**’ 0.01 ‘*’ 0.05 ‘.’ 0.1 ‘ ’ 1

Correlation of Fixed Effects:

(Intr)

exp_cc -0.014

**Block 3:**

Generalized linear mixed model fit by maximum likelihood (Laplace Approximation) ['glmerMod']

Family: binomial ( logit )

Formula: accuracy ~ 1 + exp_cc + (1 | subject) + (1 | word)

Data: get(lp_var)

| AIC | BIC | logLik | deviance | df.resid |
| --- | --- | --- | --- | --- |
| 3221.2 | 3248.1 | -1606.6 | 3213.2 | 6140 |

Scaled residuals:

| Min | 1Q | Median | 3Q | Max |
| --- | --- | --- | --- | --- |
| -7.4415 | 0.0823 | 0.1485 | 0.2495 | 1.6851 |

Random effects:

| Groups | Name | Variance | Std.Dev. |
| --- | --- | --- | --- |
| subject | (Intercept) | 4.63416 | 2.1527 |
| word | (Intercept) | 0.04274 | 0.2067 |

Number of obs: 6144, groups: subject, 128; word, 12

Fixed effects:

|  | Estimate | Std. Error | z value | Pr(>\|z\|) |  |
| --- | --- | --- | --- | --- | --- |
| (Intercept) | 3.3238 | 0.2361 | 14.077 | <2e-16 | *** |
| exp_cc | 0.3358 | 0.4268 | 0.787 | 0.431 |  |

---

Signif. codes: 0 ‘***’ 0.001 ‘**’ 0.01 ‘*’ 0.05 ‘.’ 0.1 ‘ ’ 1

Correlation of Fixed Effects:

(Intr)

exp_cc 0.001

**Block 4:**

Generalized linear mixed model fit by maximum likelihood (Laplace Approximation) ['glmerMod']

Family: binomial ( logit )

Formula: accuracy ~ 1 + exp_cc + (1 | subject) + (1 | word) + (1 | target)

Data: get(lp_var)

| AIC | BIC | logLik | deviance | df.resid |
| --- | --- | --- | --- | --- |
| 2672.4 | 2706 | -1331.2 | 2662.4 | 6139 |

Scaled residuals:

| Min | 1Q | Median | 3Q | Max |
| --- | --- | --- | --- | --- |
| -7.9247 | 0.0724 | 0.1294 | 0.2137 | 1.9325 |

Random effects:

| Groups | Name | Variance | Std.Dev. |
| --- | --- | --- | --- |
| subject | (Intercept) | 4.92304 | 2.2188 |
| target | (Intercept) | 0.04619 | 0.2149 |
| word | (Intercept) | 0.03235 | 0.1799 |

Number of obs: 6144, groups: subject, 128; target, 28; word, 12

Fixed effects:

|  | Estimate | Std. Error | z value | Pr(>\|z\|) |  |
| --- | --- | --- | --- | --- | --- |
| (Intercept) | 3.8154 | 0.2593 | 14.713 | <2e-16 | *** |
| exp_cc | 0.5524 | 0.453 | 1.219 | 0.223 |  |

---

Signif. codes: 0 ‘***’ 0.001 ‘**’ 0.01 ‘*’ 0.05 ‘.’ 0.1 ‘ ’ 1

Correlation of Fixed Effects:

(Intr)

exp_cc 0.046

**Block 5:**

Generalized linear mixed model fit by maximum likelihood (Laplace Approximation) ['glmerMod']

Family: binomial ( logit )

Formula: accuracy ~ 1 + exp_cc + (1 | subject)

Data: get(lp_var)

| AIC | BIC | logLik | deviance | df.resid |
| --- | --- | --- | --- | --- |
| 2093.1 | 2113.3 | -1043.6 | 2087.1 | 6141 |

Scaled residuals:

| Min | 1Q | Median | 3Q | Max |
| --- | --- | --- | --- | --- |
| -7.3586 | 0.0555 | 0.0622 | 0.1407 | 1.573 |

Random effects:

| Groups | Name | Variance | Std.Dev. |
| --- | --- | --- | --- |
| subject | (Intercept) | 6.455 | 2.541 |

Number of obs: 6144, groups: subject, 128

Fixed effects:

|  | Estimate | Std. Error | z value | Pr(>\|z\|) |  |
| --- | --- | --- | --- | --- | --- |
| (Intercept) | 4.5935 | 0.3226 | 14.24 | <2e-16 | *** |
| exp_cc | 0.47 | 0.5383 | 0.873 | 0.383 |  |

---

Signif. codes: 0 ‘***’ 0.001 ‘**’ 0.01 ‘*’ 0.05 ‘.’ 0.1 ‘ ’ 1

Correlation of Fixed Effects:

(Intr)

exp_cc 0.066

**Post Hoc (Block * Mapping type):**

**Block 1:**

Generalized linear mixed model fit by maximum likelihood (Laplace Approximation) ['glmerMod']

Family: binomial ( logit )

Formula: accuracy ~ 1 + mapping_type_cc + (1 + mapping_type_cc | subject) +

(1 + mapping_type_cc | target)

Data: get(lp_var)

| AIC | BIC | logLik | deviance | df.resid |
| --- | --- | --- | --- | --- |
| 8245.9 | 8299.7 | -4115 | 8229.9 | 6136 |

Scaled residuals:

| Min | 1Q | Median | 3Q | Max |
| --- | --- | --- | --- | --- |
| -2.0558 | -0.9367 | 0.5661 | 0.8795 | 1.83 |

Random effects:

| Groups | Name | Variance | Std.Dev. | Corr |
| --- | --- | --- | --- | --- |
| subject | (Intercept) | 0.32081 | 0.5664 |  |
|  | mapping_type_cc | 0.27707 | 0.5264 | -0.16 |
| target | (Intercept) | 0.03638 | 0.1907 |  |
|  | mapping_type_cc | 0.07084 | 0.2662 | -0.9 |

Number of obs: 6144, groups: subject, 128; target, 28

Fixed effects:

|  | Estimate | Std. Error | z value | Pr(>\|z\|) |  |
| --- | --- | --- | --- | --- | --- |
| (Intercept) | 0.13207 | 0.06904 | 1.913 | 0.0557 | . |
| mapping_type_cc | -0.12876 | 0.09236 | -1.394 | 0.1633 |  |

---

Signif. codes: 0 ‘***’ 0.001 ‘**’ 0.01 ‘*’ 0.05 ‘.’ 0.1 ‘ ’ 1

Correlation of Fixed Effects:

(Intr)

mppng_typ_c -0.441

**Block 2:**

Generalized linear mixed model fit by maximum likelihood (Laplace Approximation) ['glmerMod']

Family: binomial ( logit )

Formula: accuracy ~ 1 + mapping_type_cc + (1 | subject) + (1 + mapping_type_cc | word)

Data: get(lp_var)

| AIC | BIC | logLik | deviance | df.resid |
| --- | --- | --- | --- | --- |
| 5112 | 5152.4 | -2550 | 5100 | 6138 |

Scaled residuals:

| Min | 1Q | Median | 3Q | Max |
| --- | --- | --- | --- | --- |
| -6.1777 | 0.1354 | 0.2605 | 0.4648 | 2.0686 |

Random effects:

| Groups | Name | Variance | Std.Dev. | Corr |
| --- | --- | --- | --- | --- |
| subject | (Intercept) | 2.31802 | 1.5225 |  |
| word | (Intercept) | 0.0542 | 0.2328 |  |
|  | mapping_type_cc | 0.06065 | 0.2463 | 0.91 |

Number of obs: 6144, groups: subject, 128; word, 12

Fixed effects:

|  | Estimate | Std. Error | z value | Pr(>\|z\|) |  |
| --- | --- | --- | --- | --- | --- |
| (Intercept) | 1.88169 | 0.15945 | 11.801 | <2e-16 | *** |
| mapping_type_cc | 0.05767 | 0.10509 | 0.549 | 0.583 |  |

---

Signif. codes: 0 ‘***’ 0.001 ‘**’ 0.01 ‘*’ 0.05 ‘.’ 0.1 ‘ ’ 1

Correlation of Fixed Effects:

(Intr)

mppng_typ_c 0.204

**Block 3:**

Generalized linear mixed model fit by maximum likelihood (Laplace Approximation) ['glmerMod']

Family: binomial ( logit )

Formula: accuracy ~ 1 + mapping_type_cc + (1 | subject) + (1 | word)

Data: get(lp_var)

| AIC | BIC | logLik | deviance | df.resid |
| --- | --- | --- | --- | --- |
| 3213.4 | 3240.2 | -1602.7 | 3205.4 | 6140 |

Scaled residuals:

| Min | 1Q | Median | 3Q | Max |
| --- | --- | --- | --- | --- |
| -8.0895 | 0.0807 | 0.1439 | 0.2547 | 1.8463 |

Random effects:

| Groups | Name | Variance | Std.Dev. |
| --- | --- | --- | --- |
| subject | (Intercept) | 4.67859 | 2.163 |
| word | (Intercept) | 0.04562 | 0.2136 |

Number of obs: 6144, groups: subject, 128; word, 12

Fixed effects:

|  | Estimate | Std. Error | z value | Pr(>\|z\|) |  |
| --- | --- | --- | --- | --- | --- |
| (Intercept) | 3.2876 | 0.2377 | 13.829 | < 2e-16 | *** |
| mapping_type_cc | 0.299 | 0.1026 | 2.915 | 0.00356 | ** |

---

Signif. codes: 0 ‘***’ 0.001 ‘**’ 0.01 ‘*’ 0.05 ‘.’ 0.1 ‘ ’ 1

Correlation of Fixed Effects:

(Intr)

mppng_typ_c -0.045

**Block 4:**

Generalized linear mixed model fit by maximum likelihood (Laplace Approximation) ['glmerMod']

Family: binomial ( logit )

Formula: accuracy ~ 1 + mapping_type_cc + (1 | subject) + (1 | target) + (1 | word)

Data: get(lp_var)

| AIC | BIC | logLik | deviance | df.resid |
| --- | --- | --- | --- | --- |
| 2673.8 | 2707.4 | -1331.9 | 2663.8 | 6139 |

Scaled residuals:

| Min | 1Q | Median | 3Q | Max |
| --- | --- | --- | --- | --- |
| -7.7788 | 0.0738 | 0.13 | 0.211 | 1.9523 |

Random effects:

| Groups | Name | Variance | Std.Dev. |
| --- | --- | --- | --- |
| subject | (Intercept) | 4.9495 | 2.2247 |
| target | (Intercept) | 0.0465 | 0.2156 |
| word | (Intercept) | 0.03219 | 0.1794 |

Number of obs: 6144, groups: subject, 128; target, 28; word, 12

Fixed effects:

|  | Estimate | Std. Error | z value | Pr(>\|z\|) |  |
| --- | --- | --- | --- | --- | --- |
| (Intercept) | 3.8111 | 0.2603 | 14.639 | <2e-16 | *** |
| mapping_type_cc | 0.034 | 0.1195 | 0.284 | 0.776 |  |

---

Signif. codes: 0 ‘***’ 0.001 ‘**’ 0.01 ‘*’ 0.05 ‘.’ 0.1 ‘ ’ 1

Correlation of Fixed Effects:

(Intr)

mppng_typ_c -0.070

**Block 5:**

Generalized linear mixed model fit by maximum likelihood (Laplace Approximation) ['glmerMod']

Family: binomial ( logit )

Formula: accuracy ~ 1 + mapping_type_cc + (1 | subject)

Data: get(lp_var)

| AIC | BIC | logLik | deviance | df.resid |
| --- | --- | --- | --- | --- |
| 2093.5 | 2113.7 | -1043.8 | 2087.5 | 6141 |

Scaled residuals:

| Min | 1Q | Median | 3Q | Max |
| --- | --- | --- | --- | --- |
| -7.3238 | 0.0583 | 0.0605 | 0.1419 | 1.611 |

Random effects:

Groups Name Variance Std.Dev.

subject (Intercept) 6.426 2.535

Number of obs: 6144, groups: subject, 128

Fixed effects:

|  | Estimate | Std. Error | z value | Pr(>\|z\|) |  |
| --- | --- | --- | --- | --- | --- |
| (Intercept) | 4.57662 | 0.32206 | 14.21 | <2e-16 | *** |
| mapping_type_cc | 0.07708 | 0.12825 | 0.601 | 0.548 |  |

---

Signif. codes: 0 ‘***’ 0.001 ‘**’ 0.01 ‘*’ 0.05 ‘.’ 0.1 ‘ ’ 1

Correlation of Fixed Effects:

(Intr)

mppng_typ_c -0.058

**H2b:**

Formula: accuracy ~ 1 + mapping_type_cc + exp_cc + mapping_type_cc:exp_cc +

(1 + mapping_type_cc | subject) + (1 + mapping_type_cc | target)

Data: phase2_firstblock

| AIC | BIC | logLik | deviance | df.resid |
| --- | --- | --- | --- | --- |
| 8231.6 | 8298.9 | -4105.8 | 8211.6 | 6134 |

Scaled residuals:

| Min | 1Q | Median | 3Q | Max |
| --- | --- | --- | --- | --- |
| -2.0248 | -0.9337 | 0.5591 | 0.8821 | 1.8024 |

Random effects:

| Groups | Name | Variance | Std.Dev. | Corr |
| --- | --- | --- | --- | --- |
| subject | (Intercept) | 0.30501 | 0.5523 |  |
|  | mapping_type_cc | 0.23328 | 0.483 | -0.29 |
| target | (Intercept) | 0.0357 | 0.1889 |  |
|  | mapping_type_cc | 0.06888 | 0.2624 | -0.94 |

Number of obs: 6144, groups: subject, 128; target, 28

Fixed effects:

|  | Estimate | Std. Error | z value | Pr(>\|z\|) |  |
| --- | --- | --- | --- | --- | --- |
| (Intercept) | 0.13133 | 0.068 | 1.931 | 0.0535 | . |
| mapping_type_cc | -0.14634 | 0.09015 | -1.623 | 0.1045 |  |
| exp_cc | 0.24475 | 0.11472 | 2.133 | 0.0329 | * |
| mapping_type_cc:exp_cc | 0.44417 | 0.14751 | 3.011 | 0.0026 | ** |

---

Signif. codes: 0 ‘***’ 0.001 ‘**’ 0.01 ‘*’ 0.05 ‘.’ 0.1 ‘ ’ 1

Correlation of Fixed Effects:

|  | (Intr) | mppn__ | exp_cc |
| --- | --- | --- | --- |
| mppng_typ_c | -0.506 |  |  |
| exp_cc | -0.051 | 0.043 |  |
| mppng_ty_:_ | 0.044 | -0.083 | -0.314 |

**Post-Hoc (Mapping * Experiment)**

**Split for Mapping Type**

**1:1**

Generalized linear mixed model fit by maximum likelihood (Laplace Approximation) ['glmerMod']

Family: binomial ( logit )

Formula: accuracy ~ 1 + exp_cc + (1 | subject)

Data: get(lp_var)

| AIC | BIC | logLik | deviance | df.resid |
| --- | --- | --- | --- | --- |
| 5512.5 | 5531.5 | -2753.3 | 5506.5 | 4093 |

Scaled residuals:

| Min | 1Q | Median | 3Q | Max |
| --- | --- | --- | --- | --- |
| -1.7476 | -0.9316 | 0.5722 | 0.9019 | 1.7119 |

Random effects:

| Groups | Name | Variance | Std.Dev. |
| --- | --- | --- | --- |
| subject | (Intercept) | 0.2851 | 0.534 |

Number of obs: 4096, groups: subject, 128

Fixed effects:

|  | Estimate | Std. Error | z value | Pr(>\|z\|) |  |
| --- | --- | --- | --- | --- | --- |
| (Intercept) | 0.05604 | 0.05736 | 0.977 | 0.329 |  |
| exp_cc | 0.47095 | 0.11476 | 4.104 | 4.06E-05 | *** |

---

Signif. codes: 0 ‘***’ 0.001 ‘**’ 0.01 ‘*’ 0.05 ‘.’ 0.1 ‘ ’ 1

Correlation of Fixed Effects:

(Intr)

exp_cc -0.028

**1:2**

Generalized linear mixed model fit by maximum likelihood (Laplace Approximation) ['glmerMod']

Family: binomial ( logit )

Formula: accuracy ~ 1 + exp_cc + (1 | subject) + (1 | target)

Data: get(lp_var)

| AIC | BIC | logLik | deviance | df.resid |
| --- | --- | --- | --- | --- |
| 2743 | 2765.5 | -1367.5 | 2735 | 2044 |

Scaled residuals:

| Min | 1Q | Median | 3Q | Max |
| --- | --- | --- | --- | --- |
| -1.8091 | -0.9484 | 0.5652 | 0.8349 | 1.7344 |

Random effects:

| Groups | Name | Variance | Std.Dev. |
| --- | --- | --- | --- |
| subject | (Intercept) | 0.43303 | 0.658 |
| target | (Intercept) | 0.09911 | 0.3148 |

Number of obs: 2048, groups: subject, 128; target, 28

Fixed effects:

|  | Estimate | Std. Error | z value | Pr(>\|z\|) |  |
| --- | --- | --- | --- | --- | --- |
| (Intercept) | 0.20003 | 0.09846 | 2.031 | 0.0422 | * |
| exp_cc | 0.03025 | 0.15404 | 0.196 | 0.8443 |  |

---

Signif. codes: 0 ‘***’ 0.001 ‘**’ 0.01 ‘*’ 0.05 ‘.’ 0.1 ‘ ’ 1

Correlation of Fixed Effects:

(Intr)

exp_cc -0.074

**H2c:**

Generalized linear mixed model fit by maximum likelihood (Laplace Approximation) ['glmerMod']

Family: binomial ( logit )

Formula: accuracy ~ 1 + mapping_type_cc + exp_cc + mapping_type_cc:exp_cc +

acc_1 + (1 + mapping_type_cc | subject) + (1 + mapping_type_cc | target)

Data: phase2_covariate

| AIC | BIC | logLik | deviance | df.resid |
| --- | --- | --- | --- | --- |
| 8232.9 | 8306.8 | -4105.4 | 8210.9 | 6133 |

Scaled residuals:

| Min | 1Q | Median | 3Q | Max |
| --- | --- | --- | --- | --- |
| -2.0239 | -0.9347 | 0.5591 | 0.8815 | 1.8542 |

Random effects:

| Groups | Name | Variance | Std.Dev. | Corr |
| --- | --- | --- | --- | --- |
| subject | (Intercept) | 0.30281 | 0.5503 |  |
|  | mapping_type_cc | 0.23318 | 0.4829 | -0.29 |
| target | (Intercept) | 0.0356 | 0.1887 |  |
|  | mapping_type_cc | 0.06902 | 0.2627 | -0.94 |

Number of obs: 6144, groups: subject, 128; target, 28

Fixed effects:

|  | Estimate | Std. Error | z value | Pr(>\|z\|) |  |
| --- | --- | --- | --- | --- | --- |
| (Intercept) | 0.06058 | 0.10673 | 0.568 | 0.57032 |  |
| mapping_type_cc | -0.14541 | 0.09019 | -1.612 | 0.10689 |  |
| exp_cc | 0.24526 | 0.11443 | 2.143 | 0.03209 | * |
| acc_1 | 0.07964 | 0.09274 | 0.859 | 0.39049 |  |
| mapping_type_cc:exp_cc | 0.44323 | 0.14751 | 3.005 | 0.00266 | ** |

---

Signif. codes: 0 ‘***’ 0.001 ‘**’ 0.01 ‘*’ 0.05 ‘.’ 0.1 ‘ ’ 1

Correlation of Fixed Effects:

|  | (Intr) | mppn__ | exp_cc | acc_1 |
| --- | --- | --- | --- | --- |
| mppng_typ_c | -0.332 |  |  |  |
| exp_cc | -0.037 | 0.043 |  |  |
| acc_1 | -0.772 | 0.012 | 0.005 |  |
| mppng_ty_:_ | 0.034 | -0.083 | -0.315 | -0.007 |

**E1:**

Linear mixed model fit by REML. t-tests use Satterthwaite's method ['lmerModLmerTest']

Formula: log_rt ~ 1 + mapping_type_cc + block_s + mapping_type_cc:block_s +

exp_cc + mapping_type_cc:exp_cc + block_s:exp_cc + mapping_type_cc:block_s:exp_cc +

(1 + mapping_type_cc + block_s | subject) + (1 | word)

Data: phase1_correct

REML criterion at convergence: 27761.3

Scaled residuals:

| Min | 1Q | Median | 3Q | Max |
| --- | --- | --- | --- | --- |
| -3.4992 | -0.6806 | -0.1303 | 0.5916 | 5.0914 |

Random effects:

| Groups | Name | Variance | Std.Dev. | Corr |  |
| --- | --- | --- | --- | --- | --- |
| subject | (Intercept) | 0.059122 | 0.24315 |  |  |
|  | mapping_type_cc | 0.035627 | 0.18875 | -0.57 |  |
|  | block_s | 0.003419 | 0.05848 | 0.01 | 0.25 |
| word | (Intercept) | 0.003728 | 0.06106 |  |  |
| Residual |  | 0.189798 | 0.43566 |  |  |

Number of obs: 22712, groups: subject, 128; word, 12

Fixed effects:

|  | Estimate | Std. Error | df | t value | Pr(>\|t\|) |  |
| --- | --- | --- | --- | --- | --- | --- |
| (Intercept) | 7.59E+00 | 2.80E-02 | 5.53E+01 | 270.872 | < 2e-16 | *** |
| mapping_type_cc | -3.51E-01 | 1.80E-02 | 1.26E+02 | -19.443 | < 2e-16 | *** |
| block_s | -1.20E-01 | 5.70E-03 | 1.31E+02 | -21.101 | < 2e-16 | *** |
| exp_cc | 7.68E-02 | 4.35E-02 | 1.25E+02 | 1.764 | 0.08024 | . |
| mapping_type_cc:block_s | -5.07E-02 | 4.78E-03 | 2.24E+04 | -10.602 | < 2e-16 | *** |
| mapping_type_cc:exp_cc | -1.77E-02 | 3.61E-02 | 1.26E+02 | -0.491 | 0.624166 |  |
| block_s:exp_cc | -4.08E-02 | 1.14E-02 | 1.31E+02 | -3.577 | 0.000488 | *** |
| mapping_type_cc:block_s:exp_cc | 5.31E-03 | 9.55E-03 | 2.24E+04 | 0.556 | 0.578172 |  |

---

Signif. codes: 0 ‘***’ 0.001 ‘**’ 0.01 ‘*’ 0.05 ‘.’ 0.1 ‘ ’ 1

Correlation of Fixed Effects:

|  | (Intr) | mppn__ | blck_s | exp_cc | mppng_typ_cc:b_ | mppng_typ_cc:x_ | blc_:_ |
| --- | --- | --- | --- | --- | --- | --- | --- |
| mppng_typ_c | -0.424 |  |  |  |  |  |  |
| block_s | -0.004 | 0.222 |  |  |  |  |  |
| exp_cc | -0.024 | 0.018 | 0 |  |  |  |  |
| mppng_typ_cc:b_ | 0.013 | -0.079 | -0.175 | -0.002 |  |  |  |
| mppng_typ_cc:x_ | 0.014 | -0.037 | -0.008 | -0.546 | 0.001 |  |  |
| blck_s:xp_c | 0 | -0.008 | -0.03 | -0.006 | 0.007 | 0.222 |  |
| mppng__:_:_ | -0.001 | 0.001 | 0.007 | 0.016 | -0.023 | -0.078 | -0.175 |

**E2a:**

Linear mixed model fit by REML. t-tests use Satterthwaite's method ['lmerModLmerTest']

Formula: f_e2a

Data: phase2_correct

REML criterion at convergence: 26990.1

Scaled residuals:

Min 1Q Median 3Q Max

-4.4314 -0.6567 -0.1267 0.5402 4.7950

Random effects:

Groups Name Variance Std.Dev. Corr

subject (Intercept) 0.102334 0.31990

block_s 0.007026 0.08382 -0.02

word (Intercept) 0.002691 0.05187

Residual 0.168663 0.41069

Number of obs: 24528, groups: subject, 128; word, 12

Fixed effects:

Estimate Std. Error df t value Pr(>|t|)

(Intercept) 7.266e+00 3.214e-02 1.054e+02 226.052 < 2e-16 ***

mapping_type_cc 9.654e-04 5.837e-03 2.414e+04 0.165 0.86864

block_s -1.240e-01 7.712e-03 1.240e+02 -16.077 < 2e-16 ***

exp_cc 4.305e-02 5.688e-02 1.244e+02 0.757 0.45060

mapping_type_cc:block_s 2.104e-03 4.146e-03 2.427e+04 0.507 0.61182

mapping_type_cc:exp_cc -4.742e-02 1.165e-02 2.416e+04 -4.071 4.69e-05 ***

block_s:exp_cc -4.046e-02 1.542e-02 1.240e+02 -2.623 0.00981 **

mapping_type_cc:block_s:exp_cc 1.230e-02 8.291e-03 2.427e+04 1.484 0.13785

---

Signif. codes: 0 ‘***’ 0.001 ‘**’ 0.01 ‘*’ 0.05 ‘.’ 0.1 ‘ ’ 1

Correlation of Fixed Effects:

(Intr) mppn__ blck_s exp_cc mppng_typ_cc:b_ mppng_typ_cc:x_ blc_:_

mppng_typ_c -0.030

block_s -0.019 0.012

exp_cc -0.028 0.002 0.001

mppng_typ_cc:b_ 0.004 -0.164 -0.086 0.000

mppng_typ_cc:x_ 0.002 -0.100 0.001 -0.034 0.022

blck_s:xp_c 0.001 0.000 -0.034 -0.021 -0.001 0.012

mppng__:_:_ 0.000 0.023 -0.001 0.004 -0.051 -0.164 -0.086

**E2b:**

Linear mixed model fit by REML. t-tests use Satterthwaite's method ['lmerModLmerTest']

Formula: f_e2b

Data: phase2_firstblock_correct

REML criterion at convergence: 4514.9

Scaled residuals:

Min 1Q Median 3Q Max

-3.1550 -0.6881 -0.0594 0.6557 3.6856

Random effects:

Groups Name Variance Std.Dev.

subject (Intercept) 0.144889 0.38064

target (Intercept) 0.001257 0.03545

word (Intercept) 0.001129 0.03360

Residual 0.215136 0.46383

Number of obs: 3162, groups: subject, 128; target, 28; word, 12

Fixed effects:

Estimate Std. Error df t value Pr(>|t|)

(Intercept) 7.61957 0.03684 119.69210 206.854 < 2e-16 ***

mapping_type_cc 0.02894 0.01838 1816.54539 1.575 0.11553

exp_cc 0.18378 0.06975 122.23038 2.635 0.00951 **

mapping_type_cc:exp_cc -0.08105 0.03646 2203.94417 -2.223 0.02632 *

---

Signif. codes: 0 ‘***’ 0.001 ‘**’ 0.01 ‘*’ 0.05 ‘.’ 0.1 ‘ ’ 1

Correlation of Fixed Effects:

(Intr) mppn__ exp_cc

mppng_typ_c -0.074

exp_cc -0.034 0.000

mppng_ty_:_ -0.001 -0.122 -0.077

**E2c: Error in h(simpleError(msg, call)) :**

**error in evaluating the argument 'object' in selecting a method for function 'summary': object 'lmm_e2c' not found**

**Between Experiment 1 and Experiment 2**

**MEANS**

*Phase 1 Accuracy per mapping type, experiment and block*

|  |  | Experiment 1 | | Experiment 2 | |
| --- | --- | --- | --- | --- | --- |
| Block | Mapping | Mean accuracy | SD accuracy | Mean accuracy | SD accuracy |
| 1 | 1:1 | 0.49 | 0.50 | 0.53 | 0.50 |
| 1 | 1:2 | 0.42 | 0.49 | 0.39 | 0.49 |
| 2 | 1:1 | 0.75 | 0.43 | 0.76 | 0.43 |
| 2 | 1:2 | 0.59 | 0.49 | 0.61 | 0.49 |
| 3 | 1:1 | 0.86 | 0.35 | 0.85 | 0.36 |
| 3 | 1:2 | 0.69 | 0.46 | 0.72 | 0.45 |
| 4 | 1:1 | 0.91 | 0.29 | 0.89 | 0.31 |
| 4 | 1:2 | 0.78 | 0.41 | 0.79 | 0.41 |
| 5 | 1:1 | 0.92 | 0.27 | 0.92 | 0.28 |
| 5 | 1:2 | 0.80 | 0.40 | 0.83 | 0.38 |

*Phase 2 Means for Experiment and Mapping type*

| Mapping type | Experiment | Mean accuracy | SD accuracy |
| --- | --- | --- | --- |
| 1:1 | 1 | 0.83 | 0.38 |
| 1:2 | 1 | 0.81 | 0.39 |
| 1:1 | 2 | 0.77 | 0.42 |
| 1:2 | 2 | 0.79 | 0.40 |

*Phase 2 Means for Experiment and Block*

|  | Experiment 1 | | Experiment 2 | |
| --- | --- | --- | --- | --- |
| Block | Mean accuracy | SD accuracy | Mean accuracy | SD accuracy |
| 1 | 0.56 | 0.50 | 0.49 | 0.50 |
| 2 | 0.83 | 0.38 | 0.75 | 0.43 |
| 3 | 0.89 | 0.32 | 0.86 | 0.35 |
| 4 | 0.91 | 0.29 | 0.89 | 0.31 |
| 5 | 0.92 | 0.27 | 0.91 | 0.28 |

*Phase 2 Means*

| Mapping | 1:1 | | 1:2 | |
| --- | --- | --- | --- | --- |
| Block | Mean accuracy | SD accuracy | Mean accuracy | SD accuracy |
| 1 | 0.51 | 0.50 | 0.55 | 0.50 |
| 2 | 0.79 | 0.41 | 0.79 | 0.41 |
| 3 | 0.88 | 0.32 | 0.86 | 0.35 |
| 4 | 0.90 | 0.30 | 0.90 | 0.30 |
| 5 | 0.92 | 0.27 | 0.92 | 0.28 |

*Phase 2 first Block*

| Mapping type | Mean accuracy | SD accuracy |
| --- | --- | --- |
| 1:1 | 0.51 | 0.50 |
| 1:2 | 0.55 | 0.50 |

*Phase 1 correct -* *Means interaction block and mapping type*

| Mapping | 1:1 | | 1:2 | |
| --- | --- | --- | --- | --- |
| Block | Mean accuracy (ms) | SD accuracy (ms) | Mean accuracy (ms) | SD accuracy (ms) |
| 1 | 2870 | 1633 | 3264 | 1733 |
| 2 | 2164 | 1382 | 2941 | 1659 |
| 3 | 1748 | 1112 | 2617 | 1564 |
| 4 | 1563 | 913 | 2547 | 1609 |
| 5 | 1490 | 829 | 2326 | 1443 |

*Phase 1 correct -* *Means interaction Block and Experiment*

|  | Experiment 1 | | Experiment 2 | |
| --- | --- | --- | --- | --- |
| Block | Mean accuracy (ms) | SD accuracy (ms) | Mean accuracy (ms) | SD accuracy (ms) |
| 1 | 3317 | 1836 | 2642 | 1407 |
| 2 | 2492 | 1612 | 2272 | 1380 |
| 3 | 2043 | 1382 | 1954 | 1250 |
| 4 | 1899 | 1318 | 1817 | 1174 |
| 5 | 1765 | 1186 | 1725 | 1053 |

*Phase 1 correct - Means Block*

| Block | Mean accuracy (ms) | SD accuracy (ms) |
| --- | --- | --- |
| 1 | 2981 | 1671 |
| 2 | 2384 | 1506 |
| 3 | 2000 | 1320 |
| 4 | 1859 | 1251 |
| 5 | 1746 | 1123 |

*Phase 2 correct -* *Means interaction Block and Experiment*

|  | Experiment 1 | | Experiment 2 | |
| --- | --- | --- | --- | --- |
| Block | Mean accuracy (ms) | SD accuracy (ms) | Mean accuracy (ms) | SD accuracy (ms) |
| 1 | 2628 | 1567 | 2368 | 1456 |
| 2 | 1911 | 1257 | 1877 | 1141 |
| 3 | 1529 | 933 | 1636 | 1009 |
| 4 | 1397 | 744 | 1482 | 880 |
| 5 | 1327 | 724 | 1408 | 730 |

*Phase 2 correct - Means interaction Block and Experiment*

| Mapping type | Experiment | Mean accuracy (ms) | SD accuracy (ms) |
| --- | --- | --- | --- |
| 1:1 | 1 | 1667 | 1111 |
| 1:2 | 1 | 1698 | 1073 |
| 1:1 | 2 | 1701 | 1143 |
| 1:2 | 2 | 1657 | 1045 |

*Phase 2 first Block correct*

| Mapping type | Experiment | Mean accuracy (ms) | SD accuracy (ms) |
| --- | --- | --- | --- |
| 1:1 | 1 | 2598 | 1550 |
| 1:2 | 1 | 2425 | 1498 |
| 1:1 | 2 | 2689 | 1600 |
| 1:2 | 2 | 2271 | 1379 |

**Plots**

**
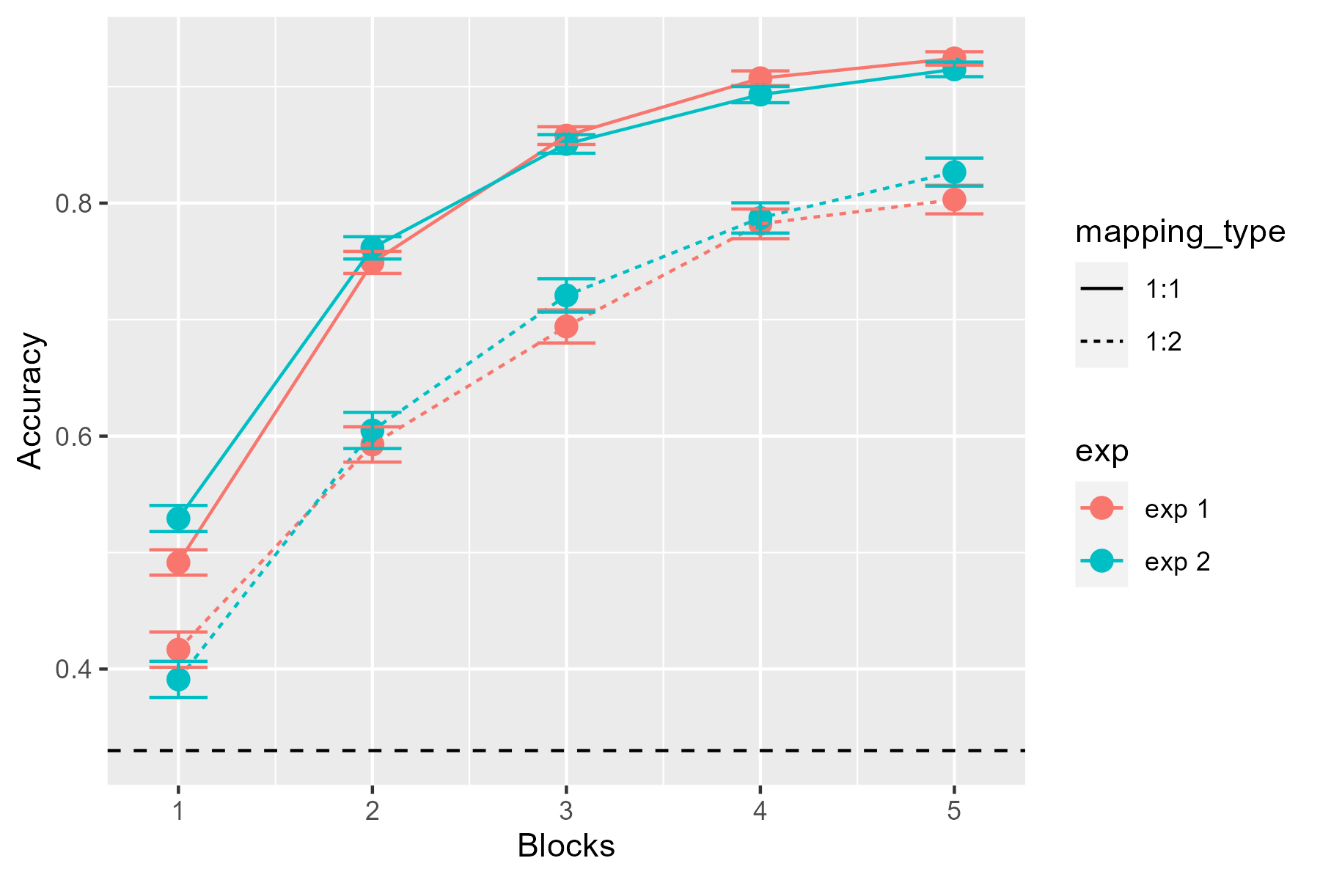
**

**
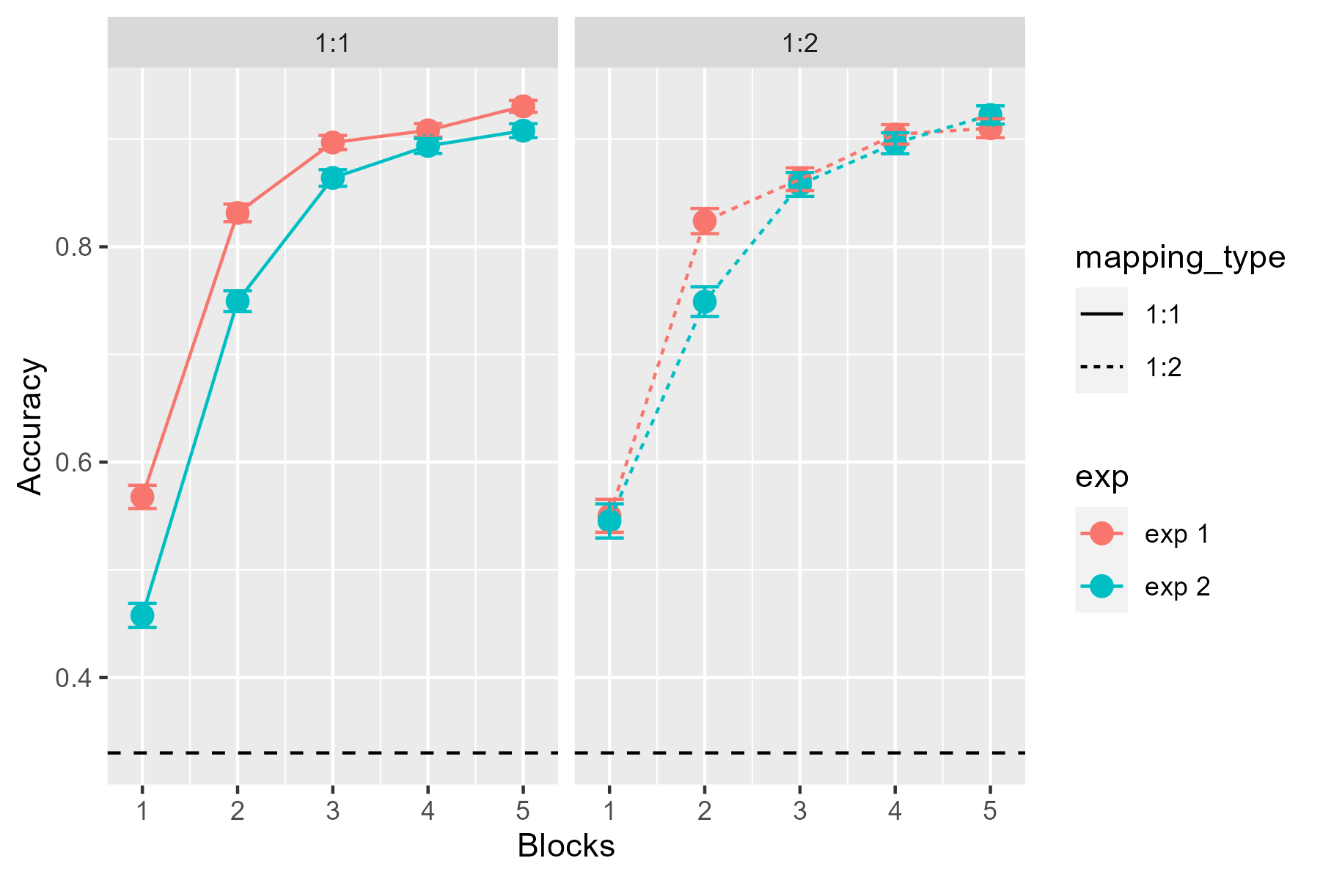

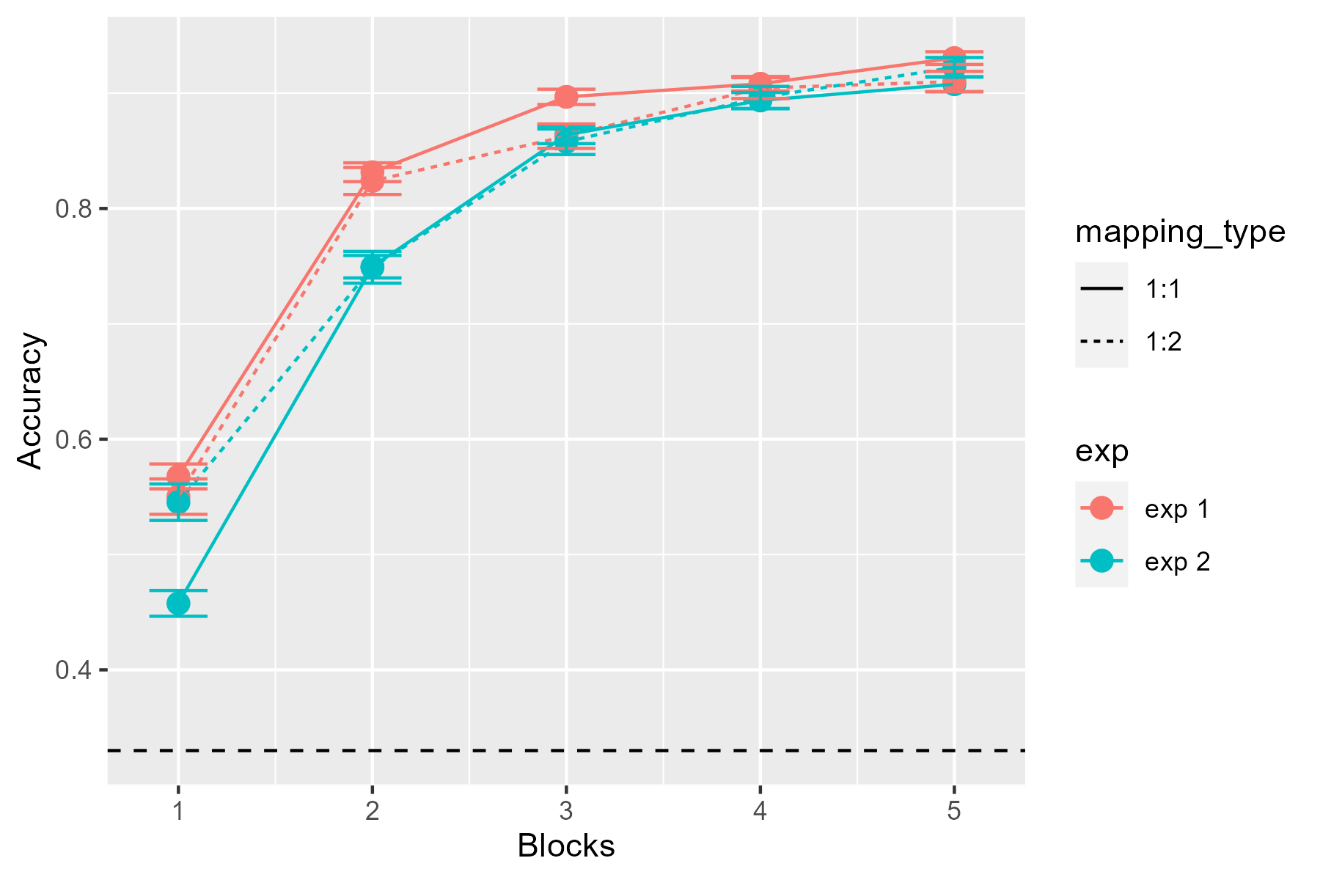
**

**
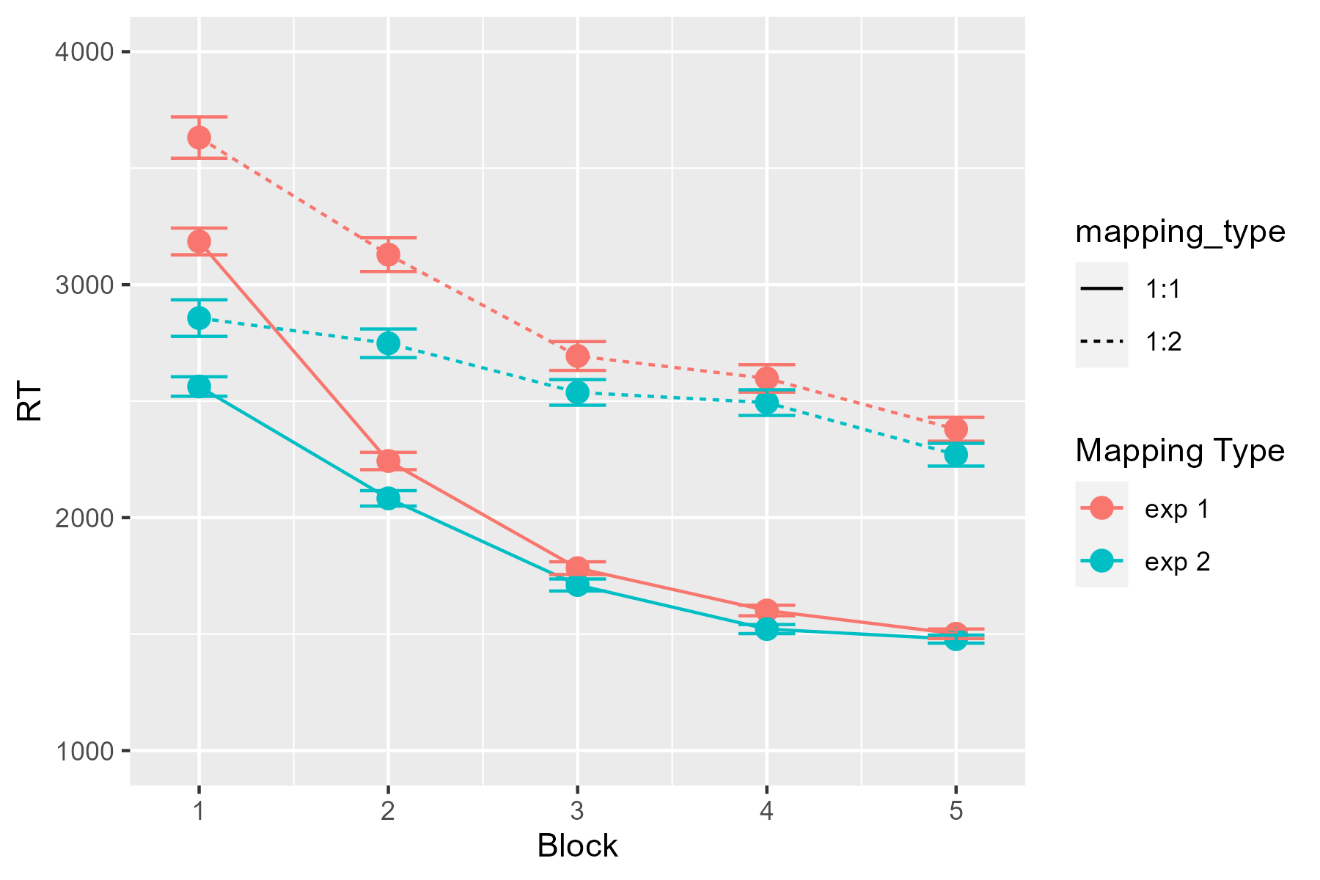
**

**
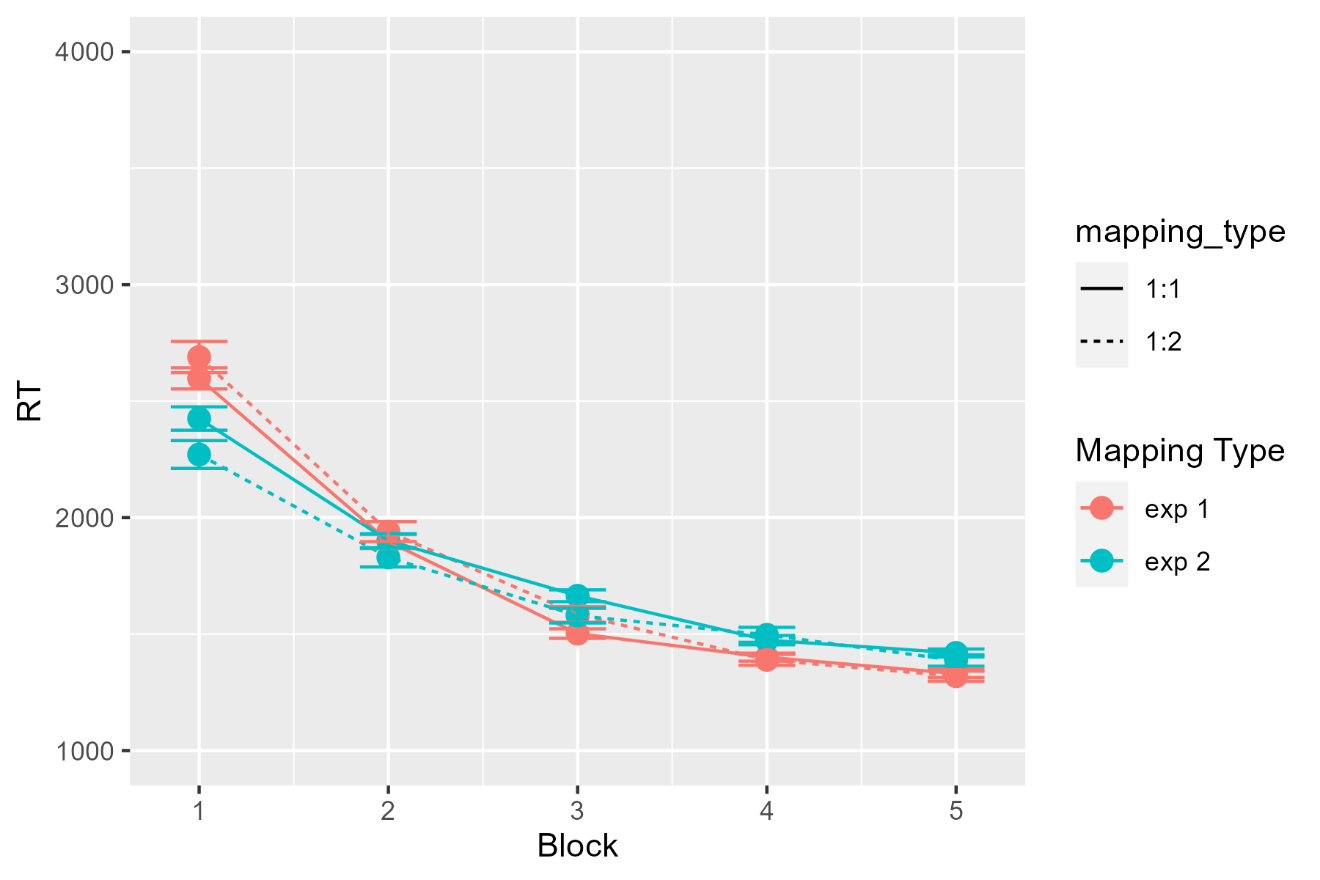

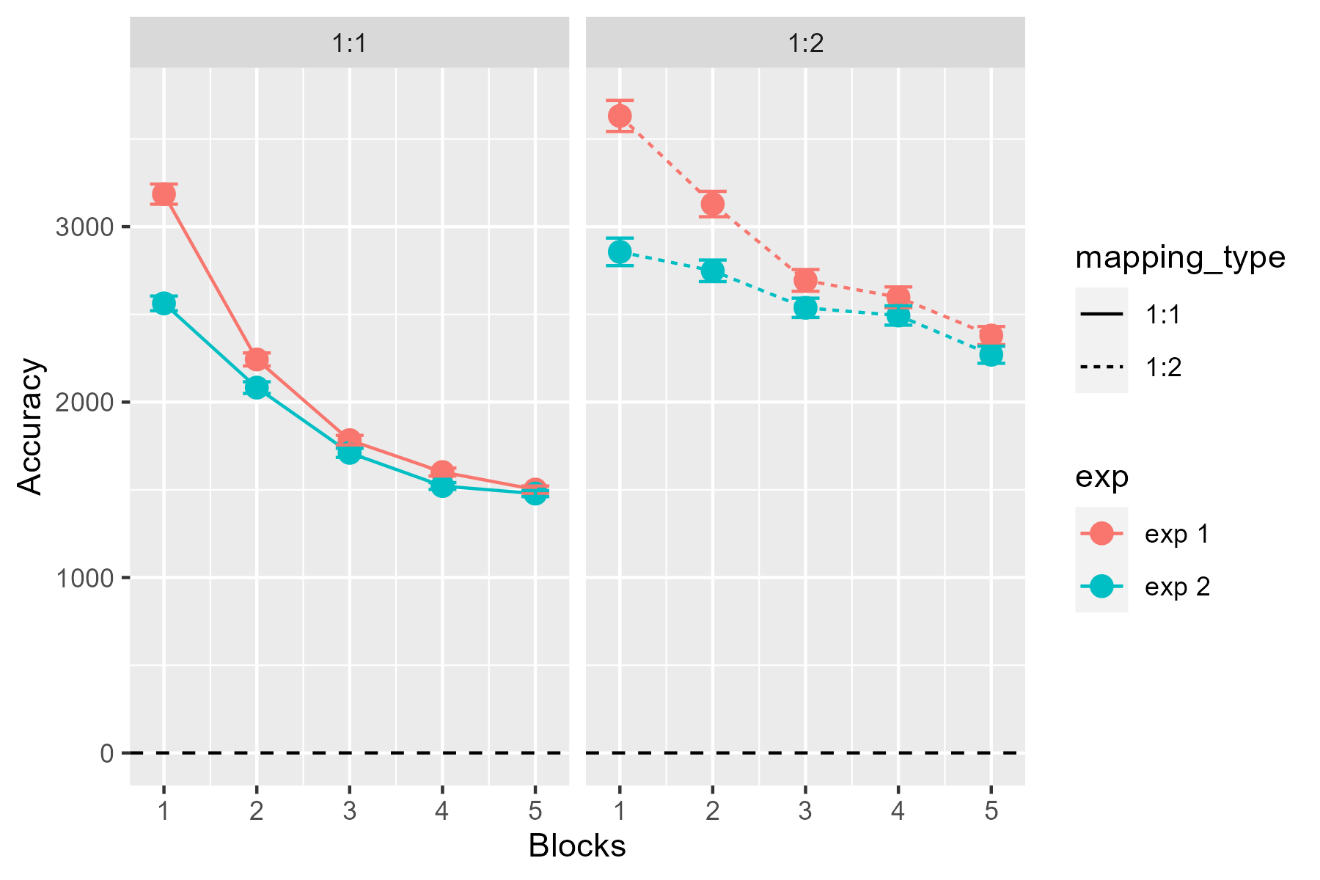

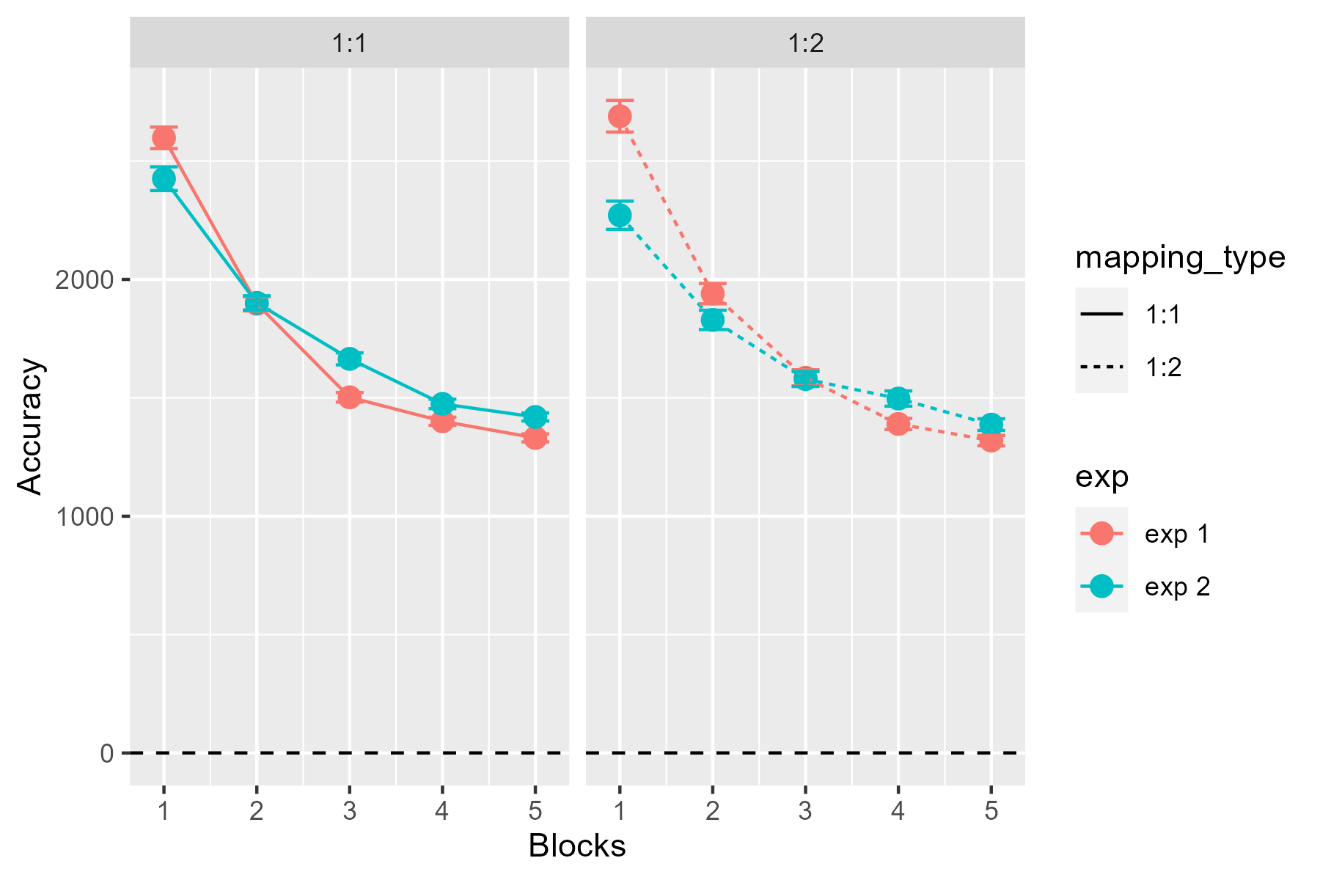
**

**Experiment 2 vs. Experiment 3**

**RESULTS**

**H1:**

Generalized linear mixed model fit by maximum likelihood (Laplace Approximation) ['glmerMod']

Family: binomial ( logit )

Formula: accuracy ~ 1 + mapping_type_cc + block_s + mapping_type_cc:block_s +

exp_cc + mapping_type_cc:exp_cc + block_s:exp_cc + mapping_type_cc:block_s:exp_cc +

(1 + mapping_type_cc | subject) + (1 + mapping_type_cc | target) + (1 + block_s + mapping_type_cc | word)

Data: phase1_34

AIC BIC logLik deviance df.resid

32477.8 32644.7 -16218.9 32437.8 31180

Scaled residuals:

Min 1Q Median 3Q Max

-10.2435 -0.7691 0.3324 0.6233 3.8317

Random effects:

Groups Name Variance Std.Dev. Corr

subject (Intercept) 0.495482 0.70390

mapping_type_cc 0.316249 0.56236 0.58

target (Intercept) 0.048402 0.22000

mapping_type_cc 0.232537 0.48222 0.34

word (Intercept) 0.029245 0.17101

block_s 0.002939 0.05421 0.08

mapping_type_cc 0.023833 0.15438 0.32 0.79

Number of obs: 31200, groups: subject, 130; target, 29; word, 12

Fixed effects:

Estimate Std. Error z value Pr(>|z|)

(Intercept) 0.94009 0.09099 10.332 < 2e-16 ***

mapping_type_cc 0.85863 0.11779 7.289 3.11e-13 ***

block_s 0.55354 0.01910 28.986 < 2e-16 ***

exp_cc -0.75668 0.12912 -5.860 4.62e-09 ***

mapping_type_cc:block_s 0.17046 0.02232 7.638 2.20e-14 ***

mapping_type_cc:exp_cc -0.18582 0.12419 -1.496 0.135

block_s:exp_cc -0.17447 0.02172 -8.035 9.39e-16 ***

mapping_type_cc:block_s:exp_cc 0.06413 0.04413 1.453 0.146

---

Signif. codes: 0 ‘***’ 0.001 ‘**’ 0.01 ‘*’ 0.05 ‘.’ 0.1 ‘ ’ 1

Correlation of Fixed Effects:

(Intr) mppn__ blck_s exp_cc mppng_typ_cc:b_ mppng_typ_cc:x_ blc_:_

mppng_typ_c 0.356

block_s 0.062 0.260

exp_cc -0.045 -0.004 -0.022

mppng_typ_cc:b_ 0.023 0.076 -0.005 -0.005

mppng_typ_cc:x_ -0.005 -0.065 -0.001 0.460 -0.078

blck_s:xp_c -0.025 0.000 -0.137 0.067 0.138 0.052

mppng__:_:_ -0.003 -0.039 0.078 0.030 -0.251 0.139 -0.006

**H2a:**

Generalized linear mixed model fit by maximum likelihood (Laplace Approximation) ['glmerMod']

Family: binomial ( logit )

Formula: accuracy ~ 1 + mapping_type_cc + block_s + mapping_type_cc:block_s +

exp_cc + mapping_type_cc:exp_cc + block_s:exp_cc + mapping_type_cc:block_s:exp_cc +

(1 + mapping_type_cc | subject) + (1 + mapping_type_cc + block_s + mapping_type_cc:block_s | target) + (1 + mapping_type_cc |

word)

Data: phase2_34

AIC BIC logLik deviance df.resid

25152.2 25352.5 -12552.1 25104.2 31176

Scaled residuals:

Min 1Q Median 3Q Max

-12.3207 0.0848 0.2451 0.4415 6.5406

Random effects:

Groups Name Variance Std.Dev. Corr

subject (Intercept) 1.55162 1.2456

mapping_type_cc 0.25854 0.5085 -0.11

target (Intercept) 0.02304 0.1518

mapping_type_cc 0.09925 0.3150 -0.25

block_s 0.01112 0.1055 0.15 -0.03

mapping_type_cc:block_s 0.05862 0.2421 -0.41 -0.12 -0.45

word (Intercept) 0.02541 0.1594

mapping_type_cc 0.04115 0.2029 0.03

Number of obs: 31200, groups: subject, 130; target, 30; word, 12

Fixed effects:

Estimate Std. Error z value Pr(>|z|)

(Intercept) 1.713938 0.124658 13.749 < 2e-16 ***

mapping_type_cc -0.147384 0.107442 -1.372 0.170

block_s 0.750657 0.026042 28.825 < 2e-16 ***

exp_cc -0.405064 0.224326 -1.806 0.071 .

mapping_type_cc:block_s 0.004904 0.056643 0.087 0.931

mapping_type_cc:exp_cc -0.080984 0.131147 -0.618 0.537

block_s:exp_cc -0.228868 0.031593 -7.244 4.34e-13 ***

mapping_type_cc:block_s:exp_cc -0.075968 0.062142 -1.223 0.222

---

Signif. codes: 0 ‘***’ 0.001 ‘**’ 0.01 ‘*’ 0.05 ‘.’ 0.1 ‘ ’ 1

Correlation of Fixed Effects:

(Intr) mppn__ blck_s exp_cc mppng_typ_cc:b_ mppng_typ_cc:x_ blc_:_

mppng_typ_c -0.100

block_s 0.077 -0.072

exp_cc -0.048 0.020 -0.021

mppng_typ_cc:b_ -0.091 0.075 -0.413 0.009

mppng_typ_cc:x_ 0.029 -0.080 0.028 -0.123 -0.056

blck_s:xp_c -0.027 0.029 -0.119 0.085 0.097 -0.092

mppng__:_:_ 0.013 -0.056 0.098 -0.027 -0.098 0.267 -0.284

**H2b:**

Generalized linear mixed model fit by maximum likelihood (Laplace Approximation) ['glmerMod']

Family: binomial ( logit )

Formula: accuracy ~ 1 + mapping_type_cc + exp_cc + mapping_type_cc:exp_cc + (1 + mapping_type_cc | subject) + (1 + mapping_type_cc |

target)

Data: phase2_firstblock_34

AIC BIC logLik deviance df.resid

8320.5 8387.9 -4150.3 8300.5 6230

Scaled residuals:

Min 1Q Median 3Q Max

-2.0984 -0.8742 -0.5157 0.9020 2.0184

Random effects:

Groups Name Variance Std.Dev. Corr

subject (Intercept) 0.35509 0.5959

mapping_type_cc 0.16281 0.4035 0.06

target (Intercept) 0.05384 0.2320

mapping_type_cc 0.19007 0.4360 -0.46

Number of obs: 6240, groups: subject, 130; target, 30

Fixed effects:

Estimate Std. Error z value Pr(>|z|)

(Intercept) 0.0197850 0.0755721 0.262 0.7935

mapping_type_cc -0.2254758 0.1112320 -2.027 0.0427 *

exp_cc 0.0006264 0.1228944 0.005 0.9959

mapping_type_cc:exp_cc 0.2933421 0.1476504 1.987 0.0470 *

---

Signif. codes: 0 ‘***’ 0.001 ‘**’ 0.01 ‘*’ 0.05 ‘.’ 0.1 ‘ ’ 1

Correlation of Fixed Effects:

(Intr) mppn__ exp_cc

mppng_typ_c -0.266

exp_cc -0.045 0.050

mppng_ty_:_ 0.060 -0.039 -0.096

**H2c:**

**Not in script … “error in evaluating the argument 'x' in selecting a method for function 'Summary': object 'lmm_hp2c_34' not found”**

**E1:**

Linear mixed model fit by REML. t-tests use Satterthwaite's method ['lmerModLmerTest']

Formula: f_e1_34

Data: phase1_correct_34

REML criterion at convergence: 25019

Scaled residuals:

Min 1Q Median 3Q Max

-4.4749 -0.6783 -0.1235 0.5882 5.3987

Random effects:

Groups Name Variance Std.Dev. Corr

subject (Intercept) 0.08008 0.28298

mapping_type_cc 0.03655 0.19118 -0.62

block_s 0.00368 0.06066 -0.03 0.15

Residual 0.18349 0.42836

Number of obs: 20975, groups: subject, 130

Fixed effects:

Estimate Std. Error df t value Pr(>|t|)

(Intercept) 7.560e+00 2.506e-02 1.268e+02 301.683 <2e-16 ***

mapping_type_cc -2.939e-01 1.801e-02 1.265e+02 -16.320 <2e-16 ***

block_s -9.493e-02 5.820e-03 1.297e+02 -16.311 <2e-16 ***

exp_cc 3.529e-02 5.012e-02 1.268e+02 0.704 0.4826

mapping_type_cc:block_s -6.094e-02 4.638e-03 2.070e+04 -13.137 <2e-16 ***

mapping_type_cc:exp_cc 5.373e-02 3.601e-02 1.265e+02 1.492 0.1382

block_s:exp_cc 1.053e-02 1.164e-02 1.297e+02 0.904 0.3675

mapping_type_cc:block_s:exp_cc -1.767e-02 9.277e-03 2.070e+04 -1.905 0.0569 .

---

Signif. codes: 0 ‘***’ 0.001 ‘**’ 0.01 ‘*’ 0.05 ‘.’ 0.1 ‘ ’ 1

Correlation of Fixed Effects:

(Intr) mppn__ blck_s exp_cc mppng_typ_cc:b_ mppng_typ_cc:x_ blc_:_

mppng_typ_c -0.589

block_s -0.039 0.135

exp_cc -0.046 0.035 0.002

mppng_typ_cc:b_ 0.009 -0.075 -0.105 -0.007

mppng_typ_cc:x_ 0.035 -0.044 -0.013 -0.589 0.005

blck_s:xp_c 0.002 -0.013 -0.046 -0.039 0.072 0.135

mppng__:_:_ -0.007 0.005 0.072 0.009 -0.048 -0.075 -0.105

**E2a:**

Linear mixed model fit by REML. t-tests use Satterthwaite's method ['lmerModLmerTest']

Formula: f_e2a_34

Data: phase2_correct_34

REML criterion at convergence: 24367.2

Scaled residuals:

Min 1Q Median 3Q Max

-3.5092 -0.6596 -0.1392 0.5319 5.3762

Random effects:

Groups Name Variance Std.Dev. Corr

subject (Intercept) 0.145492 0.38143

block_s 0.006971 0.08349 0.02

Residual 0.156985 0.39621

Number of obs: 23630, groups: subject, 130

Fixed effects:

Estimate Std. Error df t value Pr(>|t|)

(Intercept) 7.252e+00 3.361e-02 1.269e+02 215.756 < 2e-16 ***

mapping_type_cc 1.071e-02 5.405e-03 2.337e+04 1.982 0.04747 *

block_s -1.016e-01 7.612e-03 1.258e+02 -13.347 < 2e-16 ***

exp_cc 1.851e-02 6.722e-02 1.269e+02 0.275 0.78345

mapping_type_cc:block_s 1.700e-03 3.945e-03 2.338e+04 0.431 0.66663

mapping_type_cc:exp_cc -3.301e-02 1.081e-02 2.337e+04 -3.053 0.00226 **

block_s:exp_cc 3.341e-03 1.522e-02 1.258e+02 0.219 0.82663

mapping_type_cc:block_s:exp_cc 1.019e-02 7.890e-03 2.338e+04 1.292 0.19652

---

Signif. codes: 0 ‘***’ 0.001 ‘**’ 0.01 ‘*’ 0.05 ‘.’ 0.1 ‘ ’ 1

Correlation of Fixed Effects:

(Intr) mppn__ blck_s exp_cc mppng_typ_cc:b_ mppng_typ_cc:x_ blc_:_

mppng_typ_c -0.013

block_s 0.015 0.004

exp_cc -0.046 0.014 0.000

mppng_typ_cc:b_ 0.001 -0.177 -0.040 -0.002

mppng_typ_cc:x_ 0.014 -0.074 -0.007 -0.013 0.020

blck_s:xp_c 0.000 -0.007 -0.048 0.015 0.044 0.004

mppng__:_:_ -0.002 0.020 0.044 0.001 -0.080 -0.177 -0.040

**E2b:**

Linear mixed model fit by REML. t-tests use Satterthwaite's method ['lmerModLmerTest']

Formula: f_e2b_34

Data: phase2_firstblock_correct_34

REML criterion at convergence: 4176.9

Scaled residuals:

Min 1Q Median 3Q Max

-3.0136 -0.6861 -0.0842 0.6199 3.8641

Random effects:

Groups Name Variance Std.Dev.

subject (Intercept) 0.181092 0.42555

target (Intercept) 0.001934 0.04398

Residual 0.199527 0.44668

Number of obs: 3055, groups: subject, 130; target, 30

Fixed effects:

Estimate Std. Error df t value Pr(>|t|)

(Intercept) 7.53874 0.03927 133.06248 191.976 <2e-16 ***

mapping_type_cc 0.02798 0.01774 1218.98728 1.577 0.1149

exp_cc 0.01843 0.07683 126.09945 0.240 0.8108

mapping_type_cc:exp_cc -0.06454 0.03560 1168.29402 -1.813 0.0702 .

---

Signif. codes: 0 ‘***’ 0.001 ‘**’ 0.01 ‘*’ 0.05 ‘.’ 0.1 ‘ ’ 1

Correlation of Fixed Effects:

(Intr) mppn__ exp_cc

mppng_typ_c -0.028

exp_cc -0.048 0.032

mppng_ty_:_ 0.038 -0.074 -0.029

**E2c:**

**Not in script…**

**Experiment 2 vs. Experiment 3**

**MEANS**

*Means accuracy per Experiment*

| Experiment | Mean accuracy | SD accuracy |
| --- | --- | --- |
| 2 | 0.75 | 0.43 |
| 3 | 0.62 | 0.49 |

**OR**

*Means accuracy per Experiment*

| Experiment 2 | | Experiment 3 | |
| --- | --- | --- | --- |
| Mean accuracy | SD accuracy | Mean accuracy | SD accuracy |
| 0.75 | 0.43 | 0.62 | 0.49 |

*Phase 1 - Means accuracy Experiment * Block*

|  | Experiment 2 | | Experiment 3 | |
| --- | --- | --- | --- | --- |
| Block | Mean accuracy | SD accuracy | Mean accuracy | SD accuracy |
| 1 | 0.48 | 0.50 | 0.41 | 0.49 |
| 2 | 0.71 | 0.45 | 0.55 | 0.50 |
| 3 | 0.81 | 0.39 | 0.62 | 0.49 |
| 4 | 0.86 | 0.35 | 0.73 | 0.44 |
| 5 | 0.86 | 0.32 | 0.77 | 0.42 |

*Phase 2 - Means accuracy Experiment * Block*

|  | Experiment 2 | | Experiment 3 | |
| --- | --- | --- | --- | --- |
| Block | Mean accuracy | SD accuracy | Mean accuracy | SD accuracy |
| 1 | 0.49 | 0.50 | 0.51 | 0.50 |
| 2 | 0.75 | 0.43 | 0.72 | 0.45 |
| 3 | 0.86 | 0.35 | 0.78 | 0.41 |
| 4 | 0.89 | 0.31 | 0.84 | 0.37 |
| 5 | 0.91 | 0.28 | 0.86 | 0.35 |

*Main Mapping Phase 2 first Block*

| Mapping type | Experiment | Mean accuracy | SD accuracy |
| --- | --- | --- | --- |
| 1:1 | 2 | 0.46 | 0.50 |
| 1:2 | 2 | 0.55 | 0.50 |
| 1:1 | 3 | 0.50 | 0.50 |
| 1:2 | 3 | 0.52 | 0.50 |

*Phase 2 correct - interaction between Mapping and Experiment*

| Mapping type | Experiment | Mean accuracy (ms) | SD accuracy (ms) |
| --- | --- | --- | --- |
| 1:1 | 2 | 1698 | 1073 |
| 1:2 | 2 | 1657 | 1045 |
| 1:1 | 3 | 1706 | 1116 |
| 1:2 | 3 | 1710 | 1106 |

**Three-way interaction:**

- Split for block
